# Creation of an asynchronous faculty development curriculum on well-written narrative assessments that avoid bias

**DOI:** 10.1186/s12909-023-04237-w

**Published:** 2023-04-14

**Authors:** Alison S. Clay, Kathryn M. Andolsek, Kira Niederhoffer, Apoorva Kandakatla, Gloria Zhang, Meghan Price, Priya Alagesan, Sydney Jeffs, Isabel DeLaura, C. Phifer Nicholson, Saumil M. Chudgar, Aditee P. Narayan, Nancy W. Knudsen, Melinda Blazar, Pamela Edwards, Edward G. Buckley

**Affiliations:** 1 Department of Medical Education, School of Medicine, Duke University, 8 Searle Center Drive, TSCHE 1074, Durham, NC 27710 USA; 2 Department of Family Medicine and Community Health, School of Medicine, Duke University, Durham, NC USA; 3 School of Medicine, Duke University, Durham, NC USA; 4 Department of Internal Medicine, Washington University in St. Louis, St. Louis, MO USA; 5 Department of Internal Medicine, Johns Hopkins University, Baltimore, MD USA; 6 Medical Scientist Training Program, School of Medicine, Duke University, Durham, NC USA; 7 Divinity School, Duke University, Durham, NC USA; 8 Department of Medicine, School of Medicine, Duke University, Durham, NC USA; 9 Department of Pediatrics, School of Medicine, Duke University, Durham, NC USA; 10 Department of Anesthesiology, School of Medicine, Duke University, Durham, NC USA; 11 Duke Physician Assistant Program, School of Medicine, Duke University, Durham, NC USA; 12 School of Nursing, Duke University, Durham, NC USA; 13 Department of Ophthalmology, School of Medicine, Duke University, Durham, NC USA

**Keywords:** Assessment, Evaluation, Narratives, Racial bias, Undergraduate medical education, Clinical education, Faculty development

## Abstract

**Background:**

The COVID-19 pandemic in parallel with concerns about bias in grading resulted in many medical schools adopting pass/fail clinical grading and relying solely on narrative assessments. However, narratives often contain bias and lack specificity. The purpose of this project was to develop asynchronous faculty development to rapidly educate/re-educate > 2000 clinical faculty spread across geographic sites and clinical disciplines on components of a well-written narrative and methods to minimize bias in the assessment of students.

**Methods:**

We describe creation, implementation, and pilot data outcomes for an asynchronous faculty development curriculum created by a committee of volunteer learners and faculty. After reviewing the literature on the presence and impact of bias in clinical rotations and ways to mitigate bias in written narrative assessments, the committee developed a web-based curriculum using multimedia learning theory and principles of adult learning. Just-in-time supplemental materials accompanied the curriculum. The Dean added completion of the module by 90% of clinical faculty to the department chairperson’s annual education metric.

Module completion was tracked in a learning management system, including time spent in the module and the answer to a single text entry question about intended changes in behavior. Thematic analysis of the text entry question with grounded theory and inductive processing was used to define themes of how faculty anticipate future teaching and assessment as a result of this curricula.

**Outcomes:**

Between January 1, 2021, and December 1, 2021, 2166 individuals completed the online module; 1820 spent between 5 and 90 min on the module, with a median time of 17 min and an average time of 20.2 min. 15/16 clinical departments achieved completion by 90% or more faculty. Major themes included: changing the wording of future narratives, changing content in future narratives, and focusing on efforts to change how faculty teach and lead teams, including efforts to minimize bias.

**Conclusions:**

We developed a faculty development curriculum on mitigating bias in written narratives with high rates of faculty participation. Inclusion of this module as part of the chair’s education performance metric likely impacted participation. Nevertheless, time spent in the module suggests that faculty engaged with the material. Other institutions could easily adapt this curriculum with provided materials.

**Supplementary Information:**

The online version contains supplementary material available at 10.1186/s12909-023-04237-w.

## Background

The limitations of medical school grades are increasingly recognized. Grades may not be based on observation of learner skills, are difficult when learners are not supervised by the same faculty for long periods of time and often demonstrate bias against introverts, first generation medical learners and individuals historically under-represented in the health professions [1]. This bias trickles downstream, impacting selection into medical school honor societies, residency placement and career opportunities [1, 2].

Social injustice in the United States also triggers concerns about grades. The murder of George Floyd by law enforcement [3] and bullying against Asian Americans/Pacific Islanders during the coronavirus pandemic [4], increased awareness of the macro- and microaggressions from peers, teammates, supervisors and patients against our learners [5]. The pandemic also caused training interruptions and fragmented supervision [6]. Collectively, these factors heightened concerns about student evaluations [7].

Some medical schools switched to a pass/fail grading system to acknowledge presence of bias, to address the challenges of drawing summary distinctions between learners during shortened supervisory periods, and to improve student well-being [8]. Some schools terminated their honor societies [9]. Others have decried reliance on standardized subject exams and/or increased the provision of formative feedback by utilizing workplace-based assessment [10, 11]. Learners describe these interventions as increasing transparency, fairness, and overall well-being [10, 11].

However, eliminating grades increases reliance on narratives written by supervising residents, faculty and clerkship directors. Written narratives can correlate with exam performance [12], but also may contain bias. For example, certain personality descriptions are more common in narratives of learners who are women or from groups historically underrepresented in medicine (URiM) [13]. Summary descriptors in narratives (outstanding, excellent, very good, good) are also unequally distributed, unfairly biasing learners who are URiM [14].

Faculty are concerned about evaluating learners and writing narratives in the absence of grades [15]. Yearly faculty development sessions focused on evaluations and grading can improve faculty assessments [16]. Logistics of providing this faculty development are difficult when considering how to rapidly train a large number of clinical faculty spread geographically across clinical sites and intellectually across departments with different models of supervision and training. Given the ongoing pandemic in 2021 and a decision to extend pass/fail grading indefinitely in our clerkships, we sought to develop a timely and asynchronous web-based faculty development curriculum to teach faculty components of a well-written narrative and methods to minimize bias in the assessment of students.

## Methods

### Curriculum development

#### Content creation

A committee of volunteer learners and faculty from the Curriculum Committee and the Clinical Training convened. Members of affinity groups within the School of Medicine were invited to participate to further enhance the diversity in the working group. Together, the group reviewed the literature on the presence and impact of bias in clinical rotation and ways to mitigate bias in written narrative assessments. Content experts, including authors of key articles, were consulted by faculty of the committee [2, 13, 17]. After reviewing the literature, the group developed consensus around best practices for narrative assessment and strategies to reduce bias in summary evaluations for clerkships. These recommendations were developed into a storyboard for an online faculty development curriculum that could be completed asynchronously by all clinical faculty.

To engage faculty in the session and to try to motivate behavior change, the session included three objectives:

- 1) Acknowledge the presence of bias in clinical education and the assessment of clinical education.
- 2) Describe methods to set a positive learning environment where learners can succeed by setting explicit expectations for evaluation and responding in real-time to microaggressions.
- 3) Teach faculty to write a detailed narrative assessment that minimizes bias while identifying learners’ strengths and areas of growth.

#### Online module creation

We sought to create an online session that took between 15–20 min to complete, embraced multiple modalities of learning, and required learner engagement. The content was created with principles of adult learning [18], best practices for video creation, and multimedia learning theory [19–23]. Specifically, we intended for the module to be self-paced, allowing the viewer to expand the module when interested or move ahead when less interested using hot-spotting. For example, participants could click on an animated character to hear a story about bias experienced within our health system based on race, ethnicity, religion, gender, or profession (physician assistant, nurse practitioner, physical therapist, etc.). Specific areas of the module required interactivity. After learning about what contributes to making a narrative well-written, participants were asked to engage with the material by reading a narrative and deciding if the sample narrative was 1) well-written as is, 2) should be modified, or 3) was unacceptable. Answers weren’t considered right or wrong. Instead, faculty were given immediate feedback about how the sample could be modified to make the sample better. To be scored as “completed” for the Chair’s metric, participants were asked to reflect on how the session impacted them by answering a free text question: “What is one way you will change your teaching practices as a result of this module?”.

In addition to these principles of adult learning, the group considered best practices for video creation and use of multimedia learning theory [19–23] for session creation. For example, we created short discrete sections, altered delivery of content between sections (speed drawing in one section versus audio recordings of students’ experiences with bias in another section activated through hotspotting). Throughout, the group utilized both the auditory and visual channels of processing to present the maximum amount of information in the shortest period.

Once the session content and methods were outlined, the working group created a storyboard and script for the session. Visual and audio materials (including development of infographics) were delegated to individual members of the committee and then collated until the storyboard was completed. Specialists from the learning management system then placed the content into a module format and all clinical faculty were enrolled in the module.

### Participation requirements

To encourage completion of the faculty development session by faculty, the Dean of the School of Medicine added completion of the curriculum by 90% of regular rank clinical faculty to the yearly metrics by which each clinical department chairperson’s performance is measured. Our School of Medicine has four to five performance metrics each year. Chairpersons are eligible for a bonus of up to 5% of their total compensation, depending on which performance metrics are met.

Regular rank clinical faculty were chosen because they are the faculty who most often teach our students. Adjunct and consulting faculty have a wide variety of roles, responsibilities and are under less direction by the chairpersons. Completion of the module by *90%* of clinical faculty in each department was set as the target for several reasons. This was the first education metric of this kind and we wanted an achievable goal. We wanted there to be room for inherent technology failures such as a PIN station logging a person off or failing to record the person completing the module. We expected personal failures such as a faculty member not advancing to the very last slide. Furthermore, not all departmental faculty are teaching or clinical faculty (some are researchers) and some faculty might be away on leave, such as a maternity leave or a sabbatical.

### Implementation and pilot data collection

The faculty development session was deployed through the health system learning management system. Clinical department chairs were informed of the new chair’s metric and provided with an email that could be sent to their department’s clinical physician faculty, including a link to the session. Completion rates and sample reminder emails were sent to each clinical department monthly.

The learning management system automatically recorded the name of the person completing the session, their department, the date the module was opened, completion of the text-entry question, and total duration. Time spent in the module was considered a marker for “engagement” or “reaction”, a Kirkpatrick’s level one assessment of a program [24].

At the completion of 11 months, the total number of respondents were counted, and duplicate responses were removed. If an individual completed the module more than once, the first module completed was used for analysis. Using unique completions, the distribution of time spent in the module was plotted. To better understand how many individuals engaged actively with the curriculum and for how long, we eliminated those who fast forwarded through the session (spent < 5 min) or potentially walked away from computer at the health system (those who spent > 90 min in the module), and calculated average and median time spent participating in the session.

The text-entry responses indicating how a participant would change their teaching practice, was considered reflective of behavior change, a Kirkpatrick’s level 3 assessment of a program [24]. The open-entry question responses were collated from the learning management system. Using grounded thematic analysis, responses were reviewed and coded for themes, twenty responses at a time. Using constant comparison, themes were extracted until saturation was met (no additional themes were identified). Through induction, the themes were organized into categories. Open entry text responses were reviewed and counts from each response were made for each category. Though responses were short, they could include more than one category.

We avoided a pre/posttest for this faculty development module as it is prevalent in other required health system modules and we wished to distinguish this module from others. Modules with pre/posttests are commonly completed by participants skipping to the pre/post tests and simply retaking the tests until they successfully pass the test without interacting with any of the module content.

### Participants and ethical considerations

Responses from the learning management system were sent to the School of Medicine to determine faculty completion for each department for the Dean. At the time completion reports were made for the Dean, the report was redacted of personal information and the data set provided to the authors for descriptive statistics and qualitative analysis. This data was reviewed retrospectively, and data was not provided to the department chairpersons. The Duke University Institutional Review Board reviewed this project and determined it exempt from further review, including the need to obtain informed consent. This study was carried out in accordance with all with relevant guidelines and regulations from the Duke Institutional Review Board.

## Outcomes

### Curriculum

The online curriculum may be reviewed here. Just-in-time learning materials accompanied the module, including, two 5 × 7 back-to-back reference accompanied the session. One card included an infographic on how to set a positive learning climate and specific ways to respond to witnessed microaggressions (Additional file 1-created with a subscription to VennGage). A second card included specific “formulas” for writing structured feedback, do’s and don’ts when writing narratives, generalizable clinical skills to consider, and recommendations by local program directors and faculty for success within a given specialty. Twelve cards were made, including our eight required clerkships and frequently chosen electives. The back of the card included sample narratives, one that was well written, one with modest areas for improvement, and one that needed significant improvement (Additional files 2, 3, 4, 5, 6, 7, 8, 9, 10, 11, 12 and 13). These narrative examples were provided to the committee by faculty from the University of California at San Francisco and used with their permission.

The curriculum/module was developed by the student-faculty volunteer committee over a ten-week period of time, with once weekly meetings and “assigned” homework for specific individuals. Examples of homework included developing infographics, editing the script for the module, recording voiceovers, collecting information from program directors, etc. We estimate a total of 100 -130 h to review the literature, build the module, and develop the supporting documents. All individuals volunteered or completed these responsibilities in a School of Medicine role. Faculty checked in with the students to assure that the time commitment did not overwhelm them, especially as this work was completed during the pandemic and with ongoing social injustices in the United States which were upsetting and which prompted calls from many different groups for student involvement in School of Medicine committees. It took approximately two weeks for experts from the learning management system to incorporate our storyboard (as a powerpoint presentation with voiceover) into the learning management system and to build navigation and interactivity. The only additional expense was a formal voice actor who was hired by the School of Medicine clinical skills lab to provide consistent and professional narration, and to remove the likelihood that anyone might try to identify any of the students’ stories of bias.

### Completion of module

From 1/1/2021 to 11/1/2021, there were 2166 non-duplicate responses from faculty. Fifteen of 16 clinical departments met 90% completion rate for regular-rank faculty. All 15 of these departments achieved greater than a 95% completion rate, with 100% of faculty completing the module in 10 of the 15 departments.

### Time spent in session

**Fig. 1:**
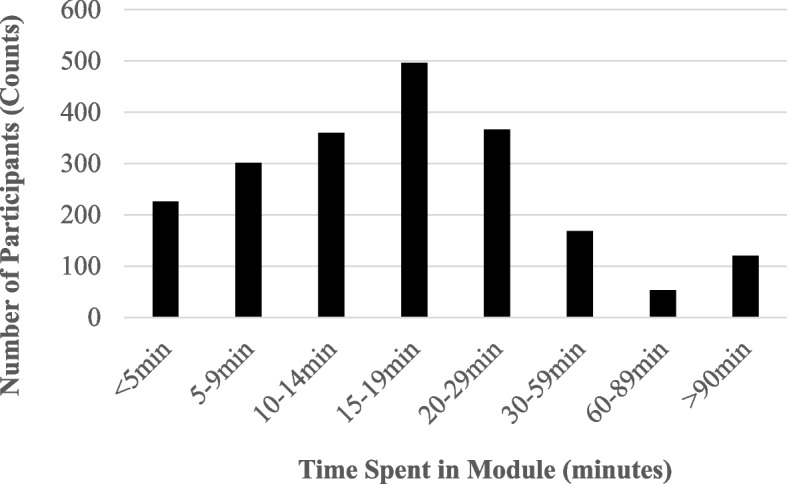
Time spent by participants in the learning module

The distribution of time spent in the session is shown in 5-min increments for 2066 participants in Fig. 1.

One thousand eight hundred twenty participants (89.6%) spent between 5 and 90 min in the curriculum, with a median time of 17 min and an average time of 20.2 min on the module. Because data was deidentified, it was not possible to determine if some of those spending less time on the module were individuals for whom the material was not relevant (e.g. for examples researchers in clinical departments who received the email requests from department chairs to complete the curriculum).

### Text-entry responses

Of the 2166 participants, 2060 (95.6%) left answers which could be analyzed for themes about how they would change their future teaching/assessment practices. Five categories of themes were developed from the text-entry responses (Table 1). The average themes/response was 1.2. The most common themes were changing specific words used in narratives (to avoid personality traits, hyperbole, etc.) and changing what was included in narratives, for example specific student skills important to that field and growth during the time of supervision.

**Table 1:** Categories of themes from text-entry responses

**Category**	**Samples**	**Frequency of Category**
Change *wording* of narrative	Add details/specifics Increase length Avoid hyperbole Avoid personality traits Think about wording Have others review wording Add structure to evaluation General statement of wanting to improve narrative	57.2% (*N* = 1239)
Change *content* of narrative	Comment on skills Comment on observations Add distinguishing characteristics Add constructive feedback Add growth Add more on the context of my experience with the learner	36.1% (*N* = 783)
Change my leadership/teaching	Include introductions Include expectations Think about use of humor Be more inclusive to the team Invite more student engagement/ownership Be vulnerable Ask learners to give me feedback Debrief at end of day Be positive Avoid politics	14.8% (*N* = 320)
Reduce/address bias	Address implicit bias Respond to microaggressions	7.5% (*N* = 164)
Other	Empty Put N/A Wrote nonsensical answer	5.4% (*N* = 117)

### Unsolicited feedback

The working group also received unsolicited feedback via email and/or through some of the text entry responses. Within the required text entry, four participants expressed cynicism or frustration at having to complete the session and 8 people included comments specifically praising the online session. The group also received a handful of emails about the module. Two specifically mentioned, “This was the best module I have ever completed.” One individual emailed the committee to request revision to the accompanying reference cards to acknowledge that some of the suggested icebreakers could contain language that is triggering (such as “what do you binge watch” which might trigger someone who had an history of/eating disorder or “tell me a story about your name” which could be triggering to someone who has experienced bias because of their name or background). The committee met to discuss these concerns and revised the card; asking supervisors to always offer more than one icebreaker question so that learners could choose one that was not triggering.

Students on the working group suggested we share the content of the module and the resources with all the learners in the Doctor of Medicine Program. Afterwards, learners asked the SOM to incorporate these materials into our curriculum, including: 1) “how to have a conversation about expectations and feedback” with supervisors using the reference cards created for the session and 2) potential opportunity to practice a simulated conversation with an actor or volunteer faculty in small peer groups.

## Discussion

We successfully implemented an asynchronous online faculty development curriculum on bias in health professions education and practical methods to reduce bias in written narratives used for student evaluation. The curriculum had high rates of completion and time spent in the mandatory module suggests that participants interacted with the material and spent time digesting the information.

We believe the success of our curriculum was attributable to several factors. Creation of timely, specific, and personalized content contributed to engagement. For example, collecting real quotes from learners about their experiences of bias at our institution, helps faculty to buy-in on the need for this training. The buy-in of the Dean was essential; both in their recognition of the need to create this training and to require the session by faculty and to hold Department Chairpersons accountable. Systems get the results they are built to achieve- including this metric as part of a Chair’s reimbursement package helps to prioritize this effort among other competing interests. As evidence of this effect, despite setting our goal at only 90% of regular rank clinical faculty completing the curriculum, 15/16 departments achieved 95% and 10/15 achieved 100%.

We believe incorporating principles of learning theory into content creation strengthened the session. Specifically by altering how material was delivered in specific sections of the session (speed drawing versus traditional slides versus student voices), adding interactivity with the material through hotspotting, and ability to expand or contract the module. We also believe that allowing faculty to evaluate sample narratives without being “right or wrong” and demonstrating how inadequate narratives could be quickly improved added to faculty engagement. Using actual narratives from our own clerkships added authenticity to this exercise.

Anecdotally, inclusion of just-in-time learning materials that specified clinical skills desired by each specialty strengthened our effort. In text entry responses, faculty specified clinical skills they might pay attention to when working with students directly from the just-in-time learning materials. Several faculty requested physical copies of the cards from the School of Medicine. Having these resources in a white coat pocket through the year makes it easier to recall content later and allows individuals to reference the cards every time they interact with a new learner.

Sharing the resources with students and involving the learners themselves improved this project. The resource cards allow students to compare expectations across specialties easily. Expectations from clerkship directors, faculty and supervising residents are often shared in different and contrasting means (e.g. verbally and on the fly or formally in lengthy orientation packet, etc.) We shared expectations in the same way, in the same location, with the same brevity for all the specialties. Students demonstrated the value of this material when they asked to practice conversations about expectations on clinical rotations as part of their required curriculum using peer-to-peer role play or with standardized actors acting as faculty. Having students use the same reference cards that faculty have seen to guide these discussions helps with clarity between both groups.

We also believe the success of this project can be attributed to involvement of our learners who have insight, energy, creativity and technical skills. Our learners were adept at creating visually stimulating infographics and organizing material in different ways than we may have chosen. The powerful stories provided by our courageous learners, although disheartening, acted as a powerful hook in the introduction of the module. Adapting this module to include the stories of learners from others’ institutions would likely strengthen the module’s impact at other schools. This is a minor modification that can easily be made by other institutions using the supplemental materials provide with this submission (see Supplemental content for a powerpoint with content that can be modified). We would recommend using voice actors to record these stories, however, to maintain learner anonymity for these sensitive stories.

### Limitations

There are limitations to our study. The most significant limitation is that we are not able to assess changes to the actual narratives which have been written for students. One of the reasons this is difficult is that the supervisory relationships within our clerkships have changed preventing a true “before” and “after”. The two classes that preceded this training and followed this training had vastly different clerkships. The “pre” class had clinical rotations disrupted by COVID-19, completing half their clerkships with a “didactic” online component followed weeks later by an abbreviated clinical experience. The “after” class had “normal” set of clinical rotations. Comparisons between narratives written more than two years apart would be confounded by other changes such as different clerkship directors, or increased use of work-place based formative feedback which might be expected to impact overall evaluation of students. For the same reason, we would expect differences in students’ reported satisfaction with the amount and quality of formative feedback and summative evaluations on their end of course evaluations. We could look at changes to learner reports of harassment and mistreatment submitted centrally, before and after faculty were required to complete this curriculum, but the pandemic and increasing awareness/acknowledgement of microaggressions were expected to and did change these reports nationally. We could determine if our students are experiencing fewer microaggressions. However, the reporting system for microaggressions was created after this module was implemented. This module could introduce new biases, which could not be detected with this pilot data and outcome evaluation.

Finally, our inclusion criteria likely missed some educators (adjunct faculty who do teach) and may have recruited some non-clinical faculty, such as researchers. This could have occurred as chairpersons, or their designees, sent blast emails to the whole department without targeting those who are clinical and who are placed on services with students. Researchers may have moved through the material quickly as it didn’t relate to them and have answered “not applicable” for the text-entry responses, but we would not have been able to remove these comments because the comments were not associated with names.

## Next steps

Our next steps will be to implement peer-to-peer coaching on summative evaluations from clerkship directors to their faculty. Clerkship directors will give feedback to evaluators about the potential presence of bias or lack of specificity in evaluations. Our clerkship directors have also agreed to review a sample of narrative assessments written by another clerkship director biannually to provide feedback to one another. Annually, the advisory deans (who write the Dean’s letters) will meet with clerkship directors in a large group to review examples of best practices and opportunities for improvement in narrative assessments.

## Conclusions

We created an asynchronous faculty development module with high rates of completion writing well-written narratives without bias. Inclusion module completion by > 90% of faculty as part of a chair’s annual performance metric likely contributed to high rates of faculty participation. However, time spent in the module suggests that faculty engaged with the material, instead of rapidly passing through the module for purposes of documenting completion. The most frequently anticipated change to teaching by faculty was changing the specific wording of narratives to include less hyperbole and more specific clinical skills. This curricula could be easily adapted to other institutions with materials provided.

## Supplementary Information

**Additional file 1.** Reference card for setting a positive climate and responding to microaggressions that accompanies the module.

**Additional file 2.** Reference card (Internal Medicine) for how to construct a well-written narrative as a supervisor for medical learners.

**Additional file 3.** Reference card (Surgery) for how to construct a well-written narrative as a supervisor for medical learners.

**Additional file 4.** Reference card (Emergency Medicine) for how to construct a well-written narrative as a supervisor for medical learners.

**Additional file 5.** Reference card (Psychiatry) for how to construct a well-written narrative as a supervisor for medical learners.

**Additional file 6.** Reference card (Pediatrics) for how to construct a well-written narrative as a supervisor for medical learners.

**Additional file 7.** Reference card (Radiology) for how to construct a well-written narrative as a supervisor for medical learners.

**Additional file 8.** Reference card (Obstetrics and Gynecology) for how to construct a well-written narrative as a supervisor for medical learners.

**Additional file 9.** Reference card (Community and Family Medicine) for how to construct a well-written narrative as a supervisor for medical learners.

**Additional file 10.** Reference card (Anesthesiology) for how to construct a well-written narrative as a supervisor for medical learners.

**Additional file 11.** Reference card (Neurology) for how to construct a well-written narrative as a supervisor for medical learners.

**Additional file 12.** Reference card (Otolaryngology) for how to construct a well-written narrative as a supervisor for medical learners.

**Additional file 13.** Reference card (Urology) for how to construct a well-written narrative as a supervisor for medical learners.

**Additional file 14.**

## Data Availability

The datasets used and/or analyzed during the current study are available from the corresponding author on reasonable request.
