# Supplementary material for: Creation of an asynchronous faculty development curriculum on well-written narrative assessments that avoid bias
Source: BMC Med Educ. 2023 Apr 14;23:244. doi: 10.1186/s12909-023-04237-w (PMC10103041; doi:10.1186/s12909-023-04237-w)
Supplement: Supplementary file 1 — Additional file 1. Reference card for setting a positive climate and responding to microaggressions that accompanies the module. [file 12909_2023_4237_MOESM1_ESM.pdf]

QUICK TIPS

For Creating a Safe and Positive Learning Environment

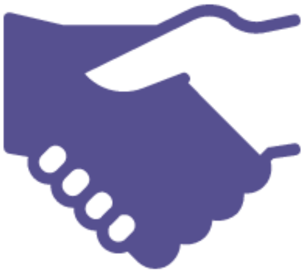

Introductions

Name/Pronouns/Program for each person
Icebreakers to find commonalities
- Last show you binged or book you read
- Favorite Superhero/Superhero Power
- Hobbies outside of healthcare
- The story behind your name

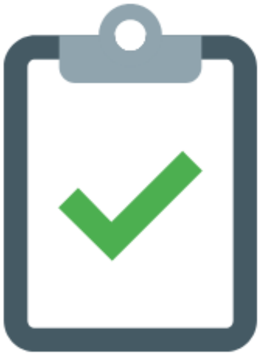

Expectations

Roles and Responsibilities for Each Person
What to Include/Length of Presentations
What to Include/when Documentation due
Conflicting Responsibilities for Team
(class, clinic, etc.)
How/When Feedback is Provided

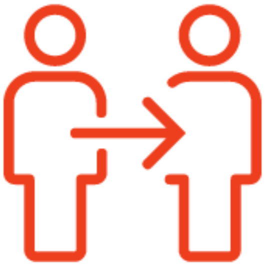

Professionalism

Be Humble/Vulnerable: "I don't know"
Acknowledge Explicit/Implicit bias
Care with Humor--it can be misinterpreted
(often based on shared or prior
experiences that students don't have)
Encourage Bystander Intervention

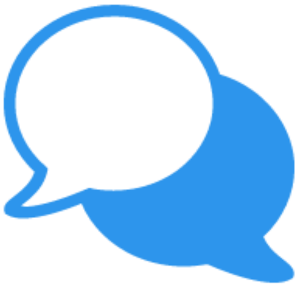

Debrief

What did we learn today?
How did we treat each other?
What went well?
What should we do differently tomorrow?
How could we be more patient-centered?
What final questions do you have?

Responding to Bias/Racism

Prepare yourself: Take a Breath, Suspend Judgement

Begin with Empathy

- "It's tough to be a patient"
- "I know you have had a lot going on recently."
- "It's hard to lose control in your life..."

State the Goals and Values Clearly:

- Every member of our team has an important role and we treat everyone, our patients and team members with respect

Inquire: Ask a curious question

- So help me understand...
- Are there questions I can answer?"

Engage toward a common goal

- "So let's get back to taking care of your"
- "How can we be sure this doesn't happen again?

Debrief: Check-in with the target of the biased behavior and Offer support/resources

1. Challenge/Disagree

What I hear you saying is that all ___are ___

I don't get it, that's not funny to me

What does that mean?

2. Emotional Response

I'm really.....
Uncomfortable
Disappointed
Frustrated
...by this conversation

3. Appeal to Sense of Self

That doesn't sound like you, are you having a bad day?

I think of you as fair-minded, so it surprises me to hear you say that...

4. Distract/Interrupt

I'm sorry, could you repeat that?

Hey, ___, I need your help with...

Anyone interested in grabbing lunch?

5. Educate/Advocate

Did you know that......

We value all our team members, we don't say that at Duke

Have you met........

6. Physical Reactions

"Gasp"
Ouch!
Slap your cheeks
Facial expression of surprise, confusion, anger, etc.
