# Supplementary material for: Creation of an asynchronous faculty development curriculum on well-written narrative assessments that avoid bias
Source: BMC Med Educ. 2023 Apr 14;23:244. doi: 10.1186/s12909-023-04237-w (PMC10103041; doi:10.1186/s12909-023-04237-w)
Supplement: Supplementary file 7 — Additional file 7. Reference card (Radiology) for how to construct a well-written narrative as a supervisor for medical learners. [file 12909_2023_4237_MOESM7_ESM.pdf]

Narrative Comments: Preceptor Guide

Radiology

Adapted from work by UCSF

Recommended Framework

I have worked with (name) for_______ shifts/days/weeks during this time

I have directly observed _________

Overall (name) functions:

- ☐ **below the level expected of a student in this clerkship**
- ☐ **at the level expected of a student in this clerkship**
- ☐ **above the expected level for a student in this clerkship**
- ☐ **at the level of a sub-intern**
- ☐ **at the level of an intern**

BECAUSE (describe the performance using **specific examples** that describe student's skills i.e.: patient care skills, medical knowledge and reasoning, Ddx ability, communication/ professionalism when possible add **observations** about student's **progress** or **growth** throughout the clerkship

General Skills

- **Patient care skills:** history and physical exam, oral presentation, written notes, procedures, follow up on patients
- **Medical knowledge base, clinical reasoning**
- **Practice-based learning:** use of technology and evidence based medicine, responds well to feedback (ability for self-reflection and improvement)
- **Interpersonal and communication skills:** interactions with team, patients and families
- **Professionalism:** set boundaries, demonstrate accountability and dependability, act ethically
- **Systems-based practice:** can navigate healthcare system
- **Inter-professional collaboration:** with nurses, tech, social work, PT

Specialty Specific Skills

- **Professionalism:** engaged and self-motivated. Proactive with getting involved with procedures and cases. Polite, courteous, and respectful. Genuine
- **Critical thinking:** asked excellent questions, interested in understanding the indication and rationale for imaging, as well as the understanding the imaging findings
- **Passion:** "showed up early and stayed late every day and even on days not scheduled to be on service". Strong interest and desire to learn as much about radiology as possible
- **Team player:** works well with others, well-liked by ancillary staff, no job is too small to help the patient.
- **Diversity:** active listener. Connects with patients of all backgrounds. Strong compassion and empathy. Examples of when the student went above and beyond in caring for a patient.

Do's

1. Focus on **competencies** and **skill**
2. Provide **specific information** about student's clinical skills (history taking, physical exam, oral presentation, clinical reasoning, etc.).
3. Comment on student's level of initiative, enthusiasm, and ability to self-start.
4. Assess the student's ability to work with **patients, peers, residents and faculty, and other members of the healthcare team.**
5. Be thoughtful with your use of superlatives and descriptors so as to **avoid hyperbole**

Don'ts

a. Use **"coded" adjectives** (e.g. outstanding, excellent) **without any behavioral examples**

b. Use vague generalities without examples.

c. Reward students for coming in early or staying late by using it as an example of positive behavior when it may violate the student work hours policy

d. **Reference grades** for core clerkship students (e.g., She would receive Honors if this were an option)

e. Allow **bias** to influence your comments*

f. **Fill the space with a lot of 'cheerleading' comments** that do not describe performance (i.e. avoid reliance on "we predict he has a bright future" or "she is sure to have a continued upward trajectory and make a fine house officer.")

Preceptor Narratives: Example Comments

Below, please find examples of narrative comments that would be considered "good", "intermediate" and "poor". The reasons for these classifications are mapped onto the "Do's" and "Don'ts" with number citations

Example 1

Good:

- Student excelled in the early part of this eight week clerkship. She has a deep fund of knowledge and is already an integral part of the trauma team. She took pride in accomplishing the tasks she was asked to perform and was reliable in completing all tasks. She prepared several talks on clinical topics we were seeing and presented these in an exceptional manner **(1)** (more like a resident than a student). She had exceptionally thorough history and physicals with stellar review of systems **(2)**. Her presentations were clear and succinct. She received feedback from me over time, and each time I noted subsequent improvement in the relevant area **(4)**. (Student) is a professional and empathic student who developed strong relationships with her patients **(4,5)**. She helped several families as their loved one underwent surgery **(4)**. Student will undoubtedly mature into an exceptional physician.

Example 2

Intermediate:

- A wonderful person all around **(a, b)**. He is improving his fund of knowledge daily and never hesitated asking great questions. His patients enjoyed him thoroughly and I felt he could easily be relied upon to follow through with tasks.

  Area for improvement: Need more specific examples; nothing particularly unique or commenting on student growth

- Student) is a top tier student and is among the upper few percent of third year students that I have observed in 5 years of attending on the service- she would definitely have received honors **(a, d)**. She is functioning at the level of a subintern, has a keen curiosity about the clinical problems that she encounters and an excellent fund of knowledge. I am delighted to recommend her highly and have offered to write a letter for her as part of her internship application process **(f)**.

  Need more discussion of tangible skills; too many hyperboles and coded language- clearly an outstanding student, but not clear exactly why

Example 3

Poor

- A pleasure to work with, and a valuable asset to the clinic-- dependable. He will be an excellent physician in whichever field he chooses.
- A joy to have on the team. Very motivated and contributed a great deal in presentations and extra reading. Student is an active learner

  Area for improvement: Need more specific clinical examples; does not mention how student interacted with team or patients. use of coded language that does not indicate what specifically sets this student apart. Could better address growth and progression of student.
