# Supplementary material for: Creation of an asynchronous faculty development curriculum on well-written narrative assessments that avoid bias
Source: BMC Med Educ. 2023 Apr 14;23:244. doi: 10.1186/s12909-023-04237-w (PMC10103041; doi:10.1186/s12909-023-04237-w)
Supplement: Supplementary file 14 — Additional file 14. [file 12909_2023_4237_MOESM14_ESM.pptx]

## Slide 1
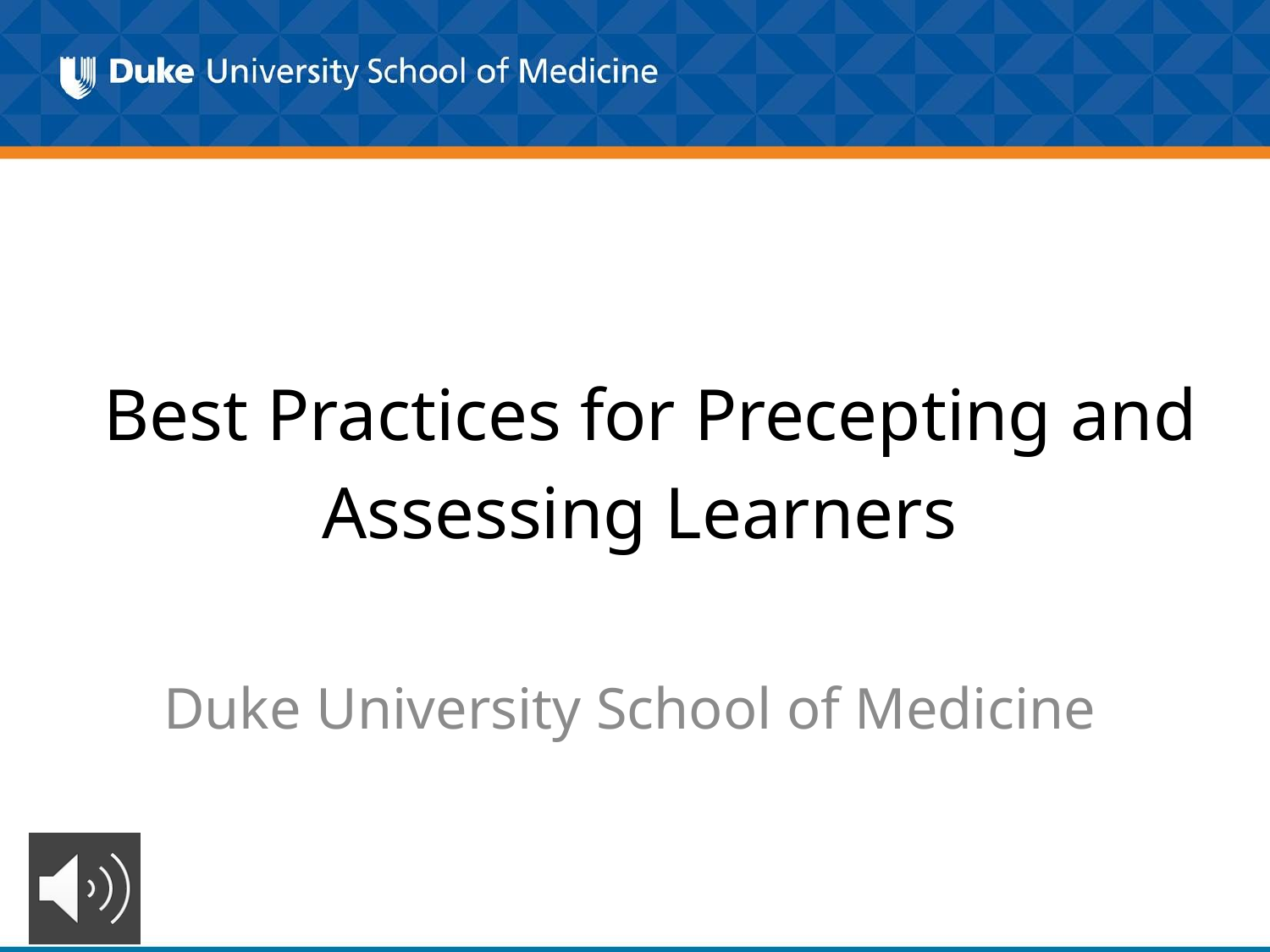

# Best Practices for Precepting and Assessing Learners
Duke University School of Medicine

## Slide 2
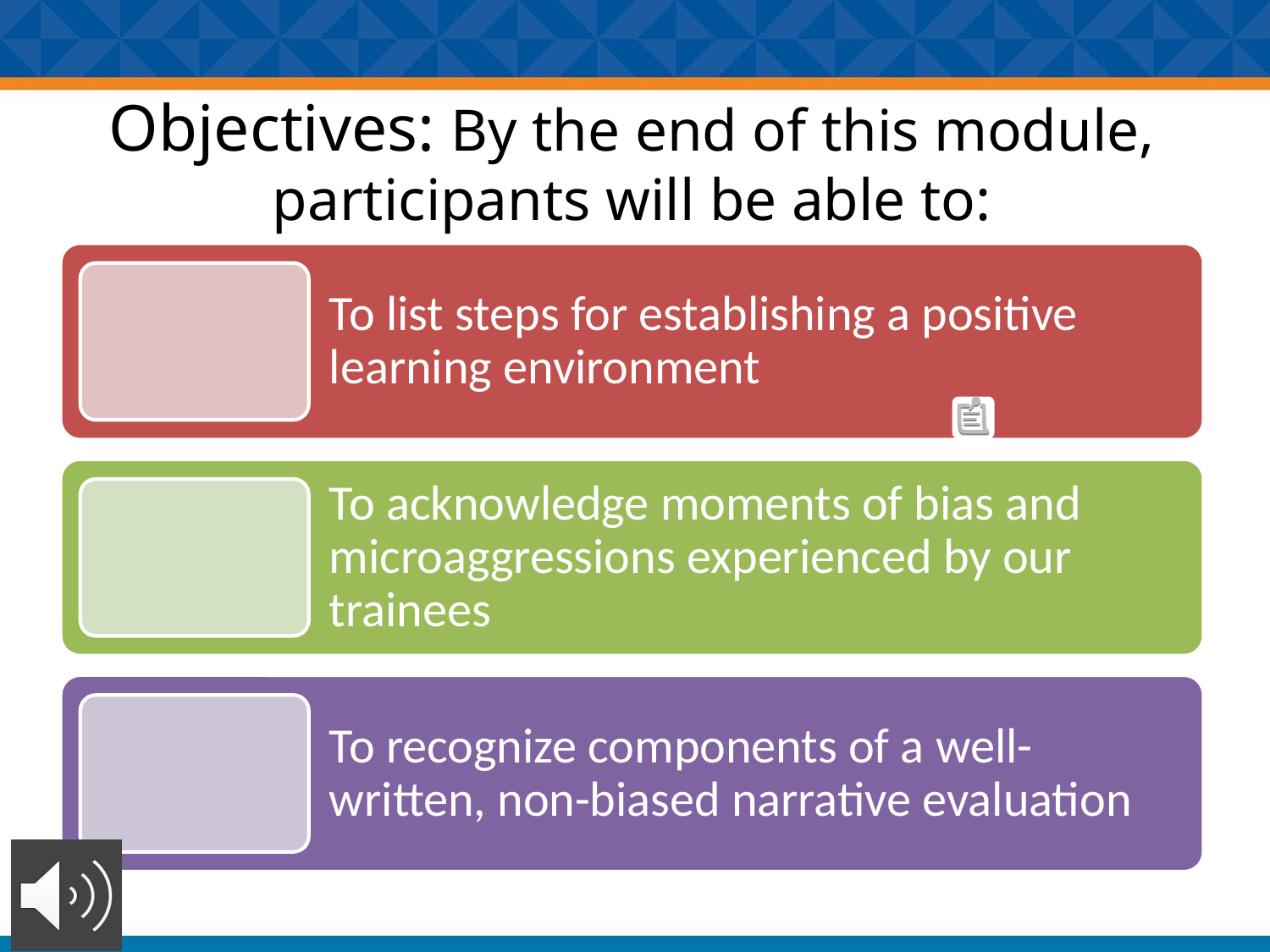

# Objectives: By the end of this module, participants will be able to:

## Slide 3
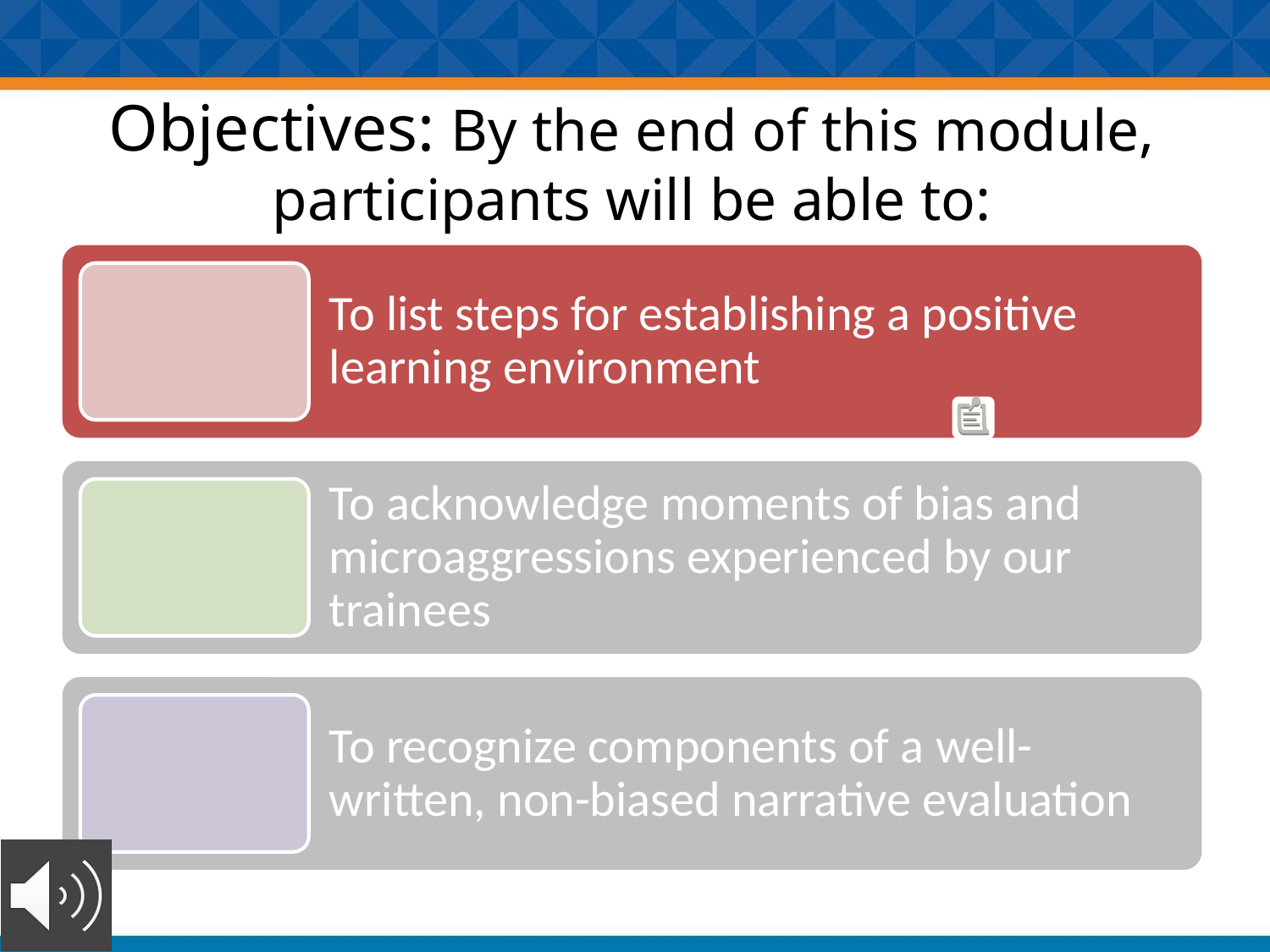

# Objectives: By the end of this module, participants will be able to:

## Slide 4
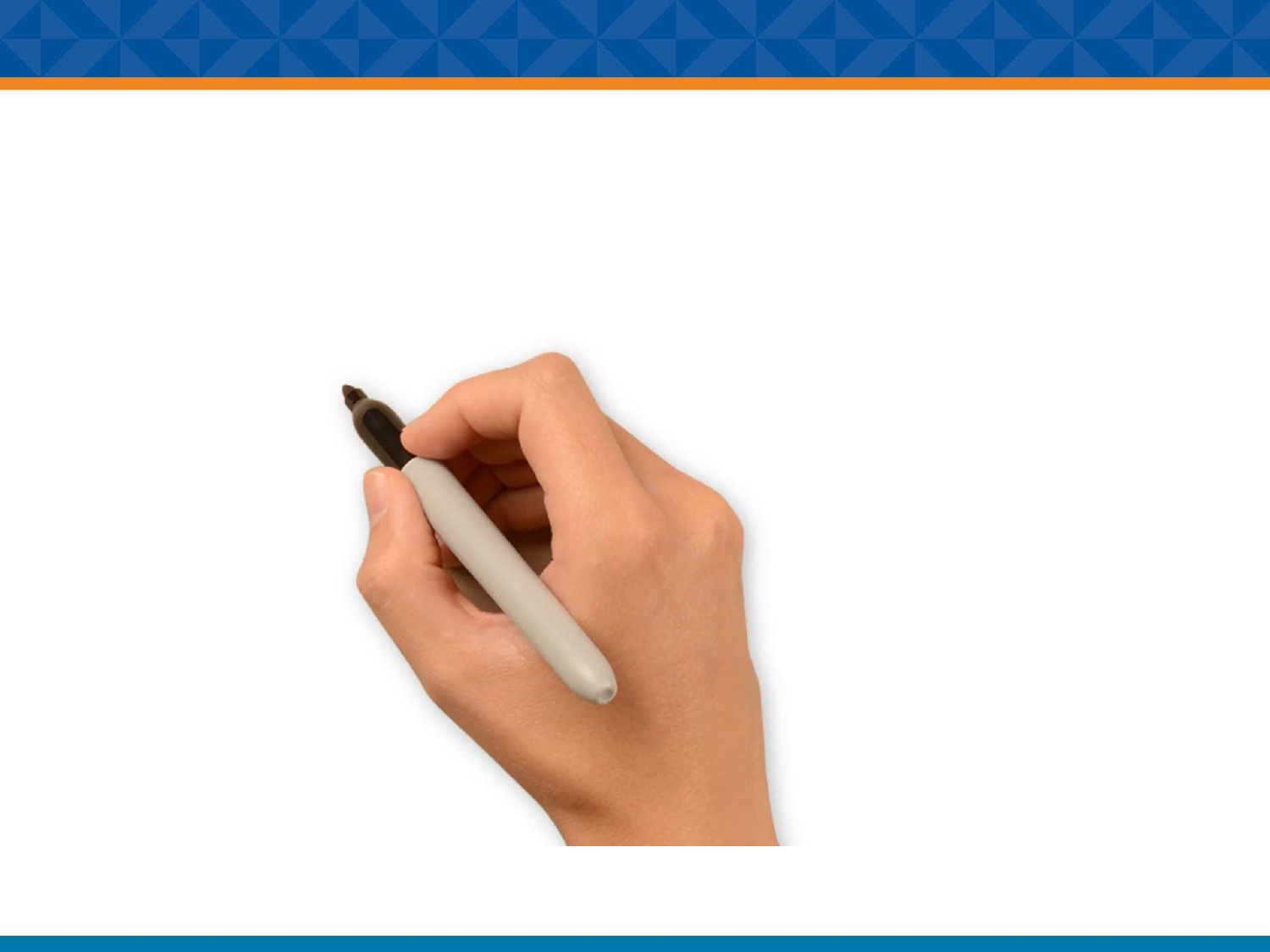

## Slide 5
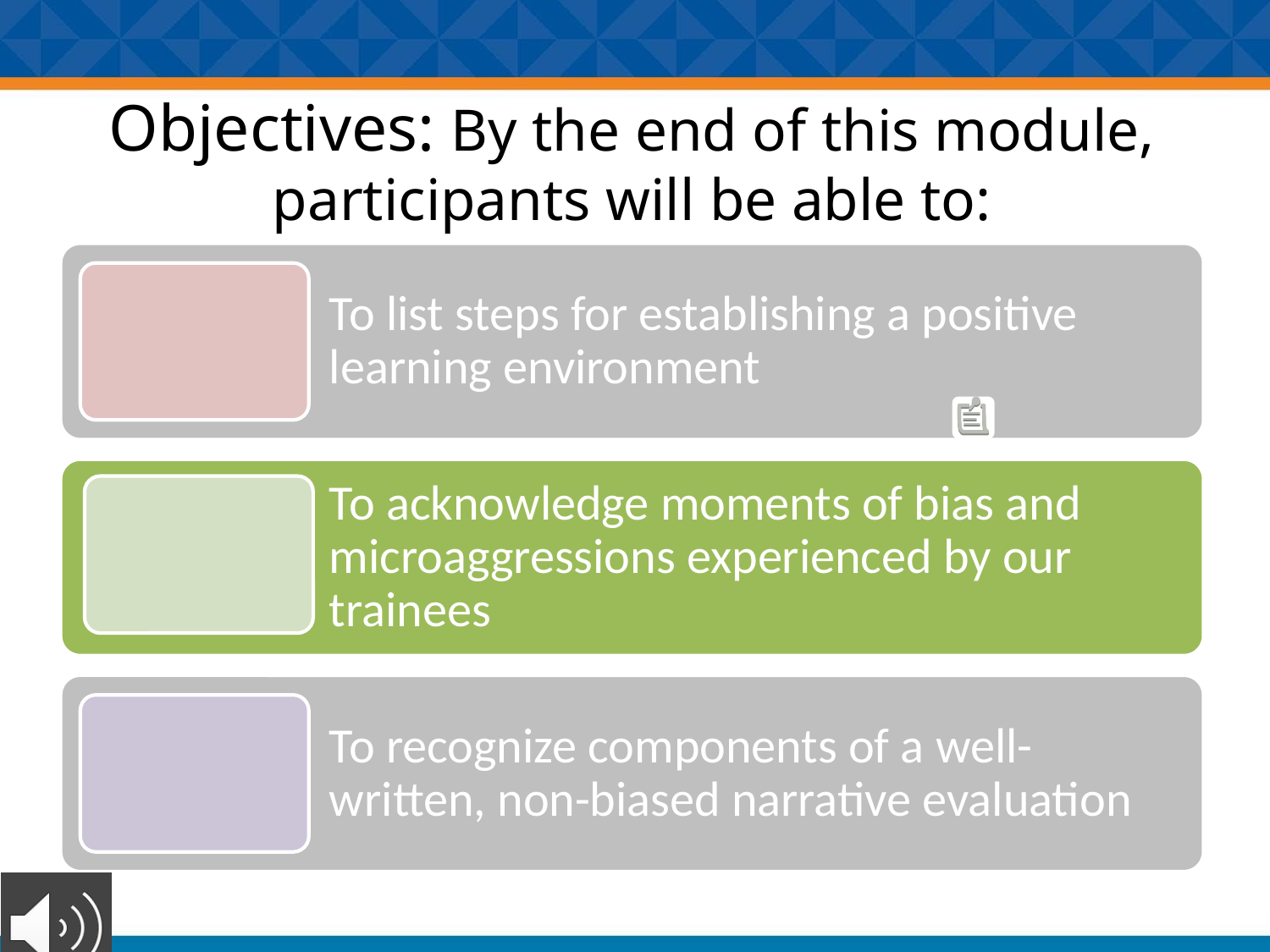

# Objectives: By the end of this module, participants will be able to:

## Slide 6
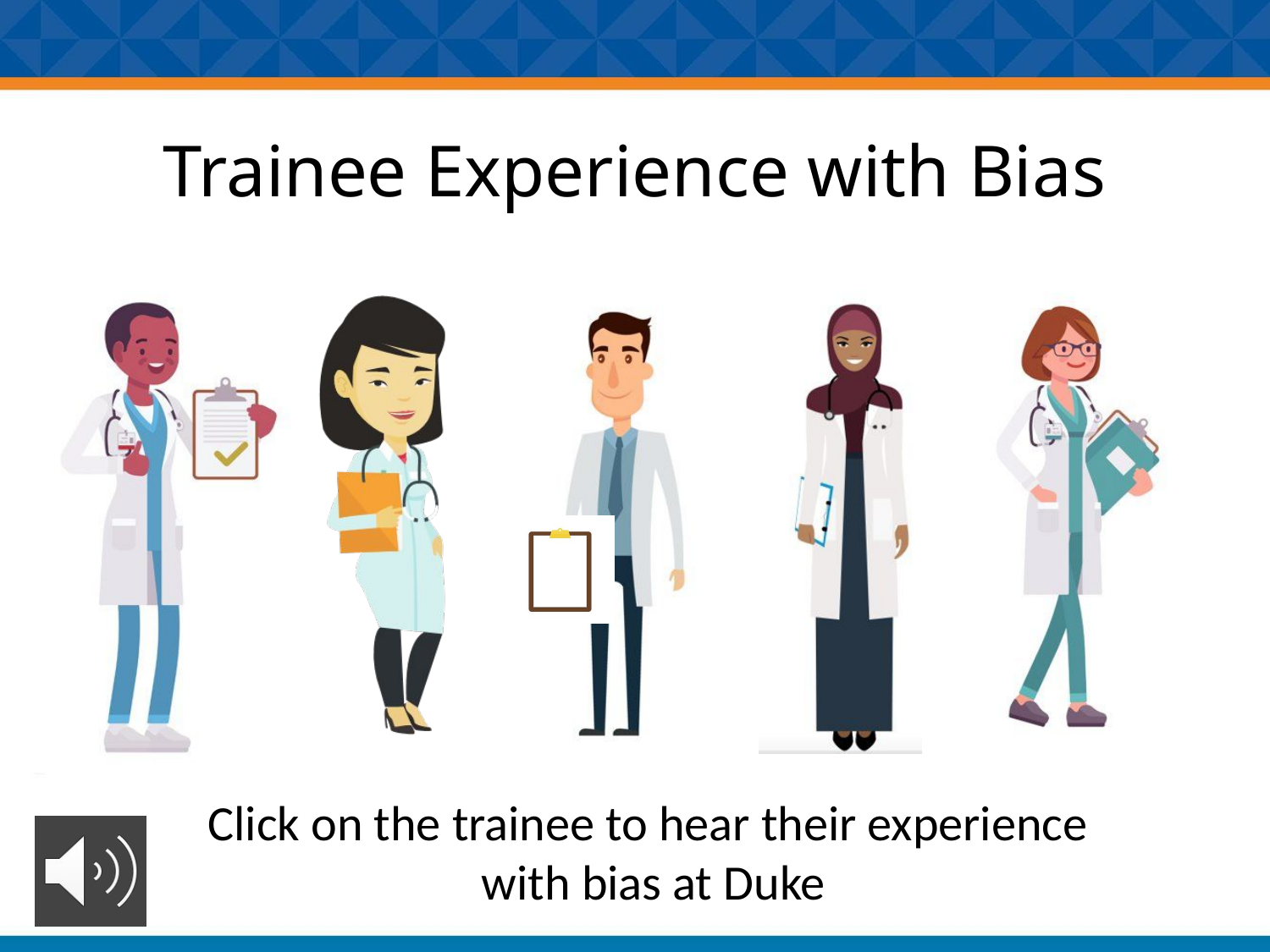

# Trainee Experience with Bias
Click on the trainee to hear their experience
with bias at Duke

## Slide 7
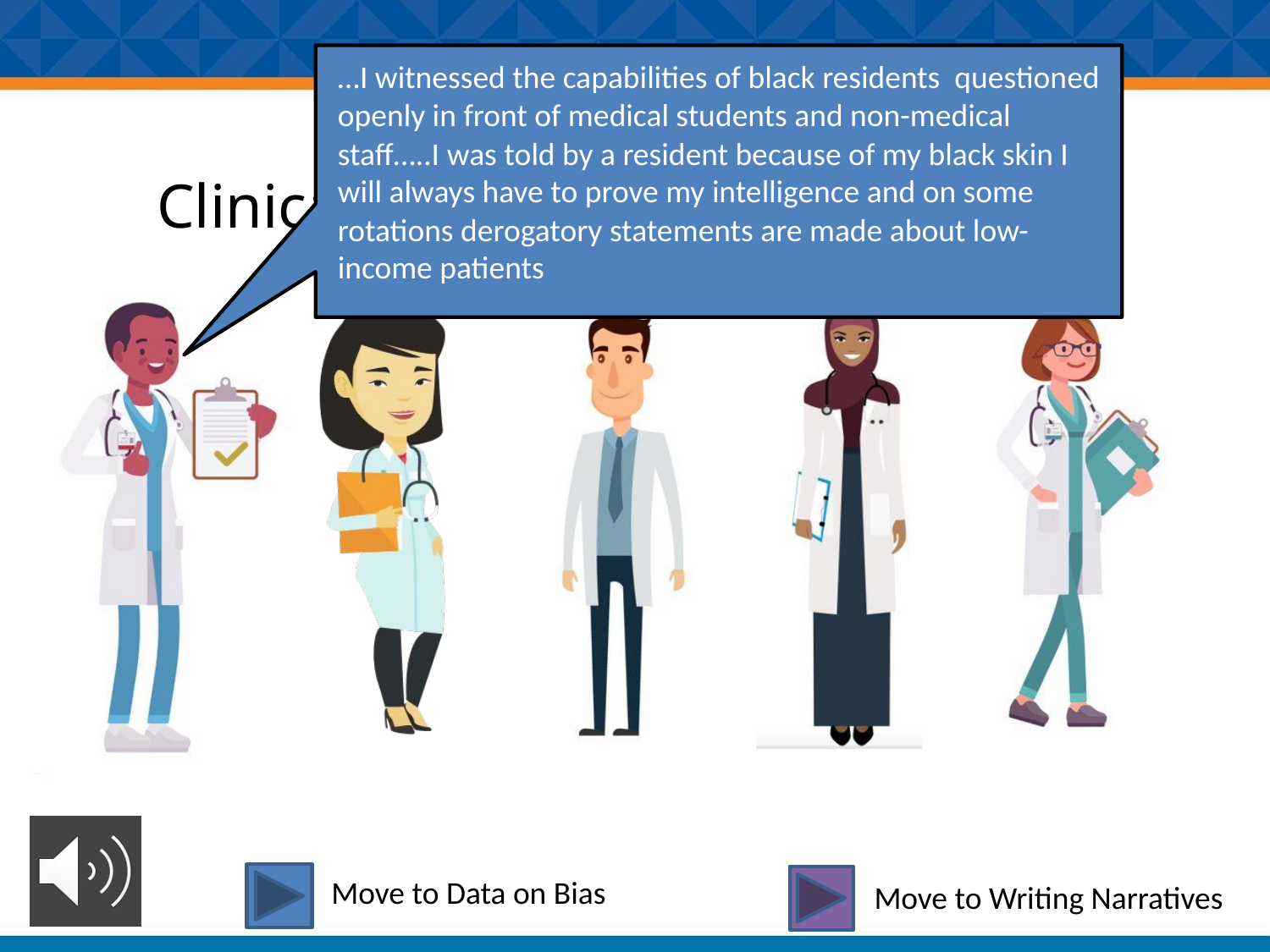

…I witnessed the capabilities of black residents questioned openly in front of medical students and non-medical staff…..I was told by a resident because of my black skin I will always have to prove my intelligence and on some rotations derogatory statements are made about low-income patients
# Bias in Clinical Education and Evaluations
Move to Data on Bias
Move to Writing Narratives

## Slide 8
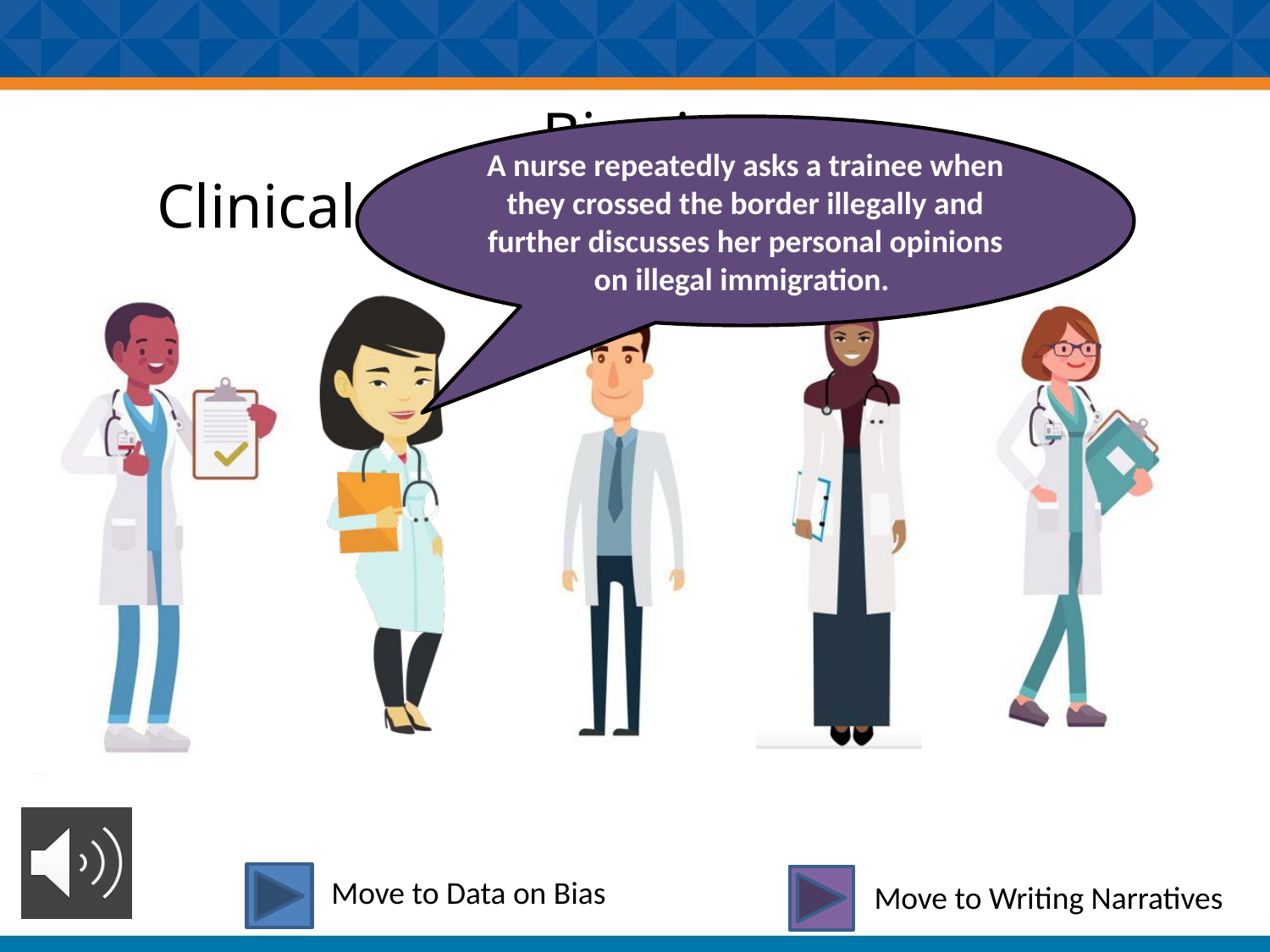

# Bias in Clinical Education and Evaluations
A nurse repeatedly asks a trainee when they crossed the border illegally and further discusses her personal opinions on illegal immigration.
Move to Data on Bias
Move to Writing Narratives

## Slide 9
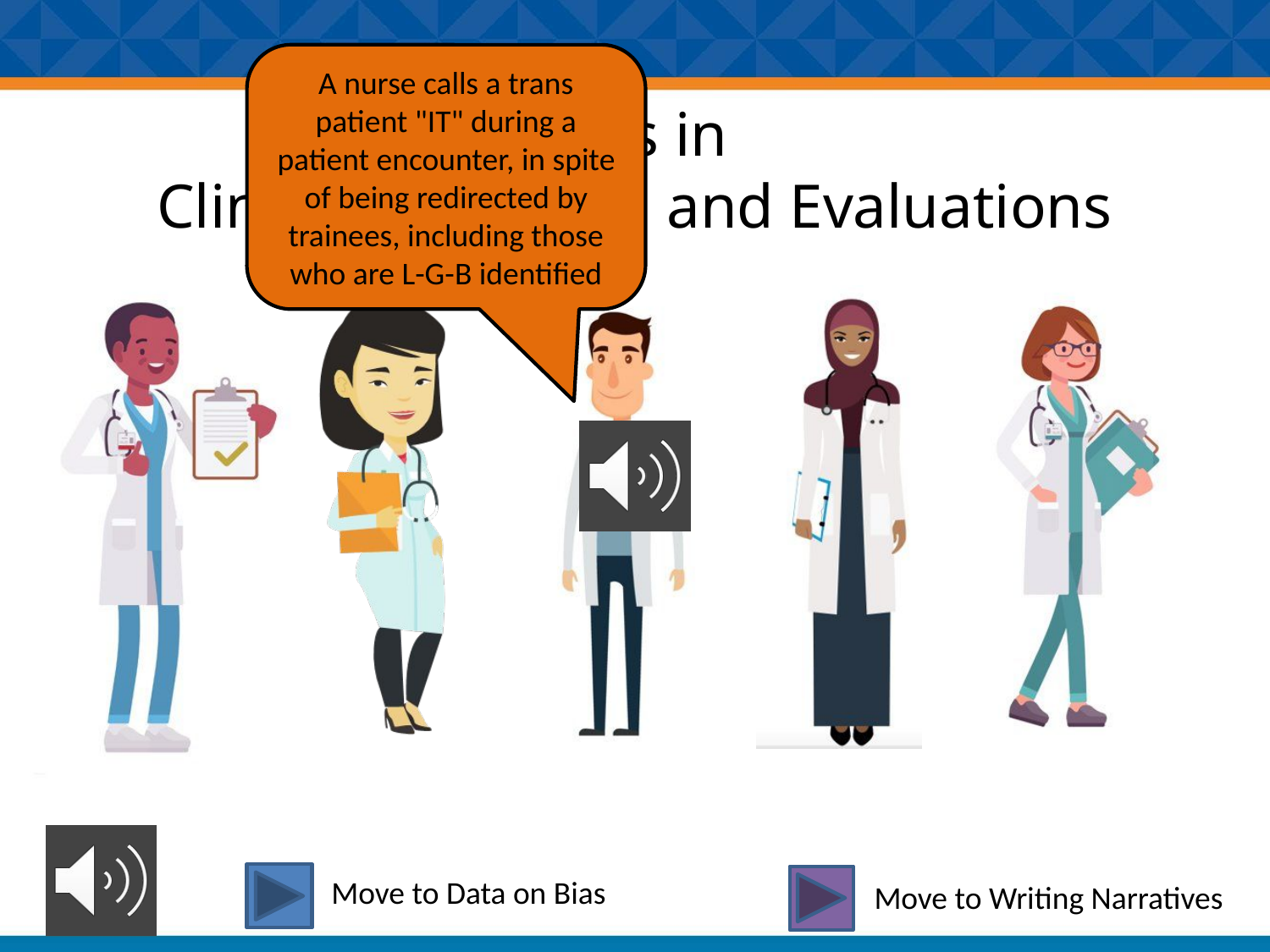

A nurse calls a trans patient "IT" during a patient encounter, in spite of being redirected by trainees, including those who are L-G-B identified
# Bias in Clinical Education and Evaluations
Move to Data on Bias
Move to Writing Narratives

## Slide 10
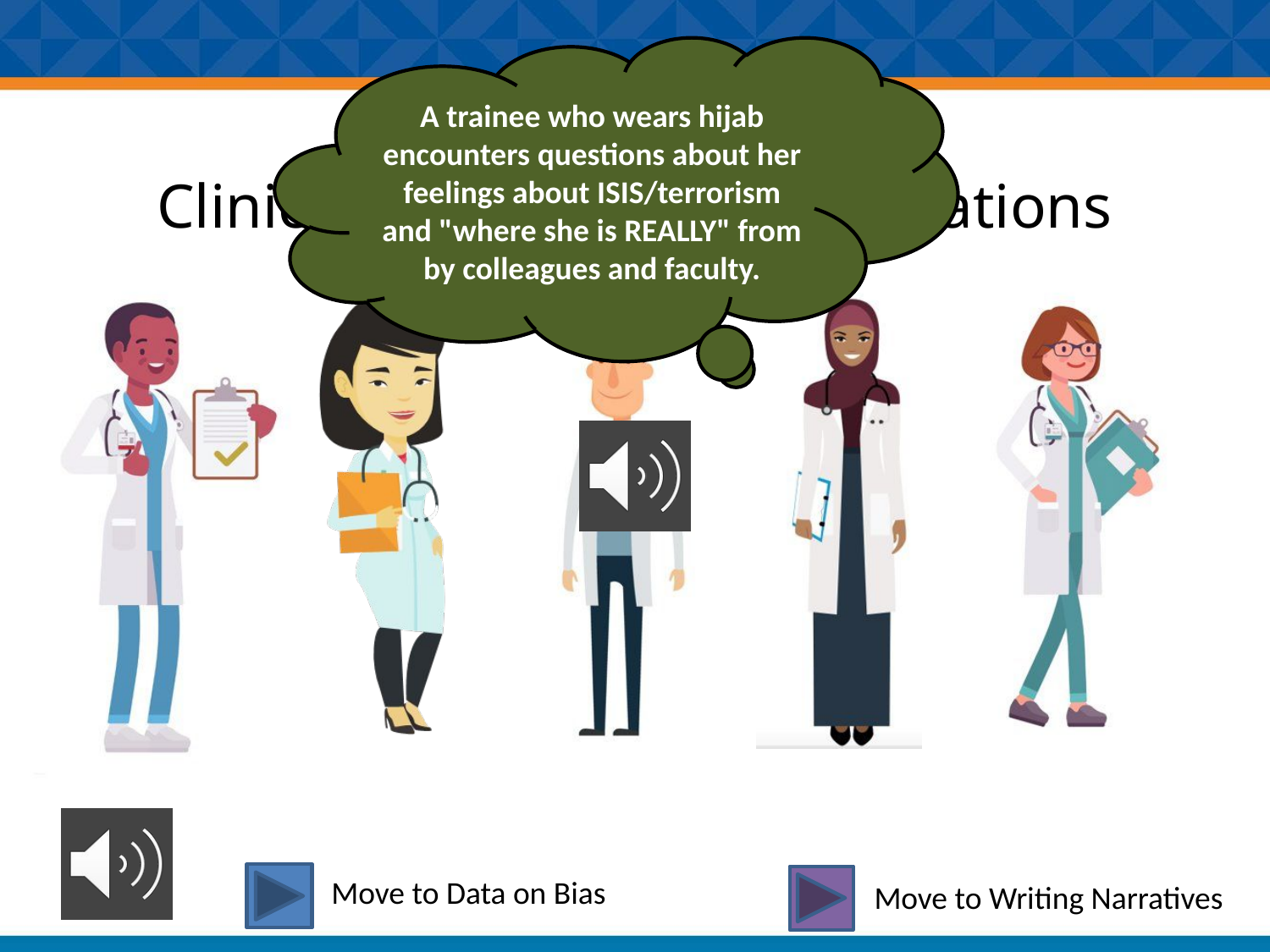

A trainee who wears hijab encounters questions about her feelings about ISIS/terrorism and "where she is REALLY" from by colleagues and faculty.
# Bias in Clinical Education and Evaluations
Move to Data on Bias
Move to Writing Narratives

## Slide 11
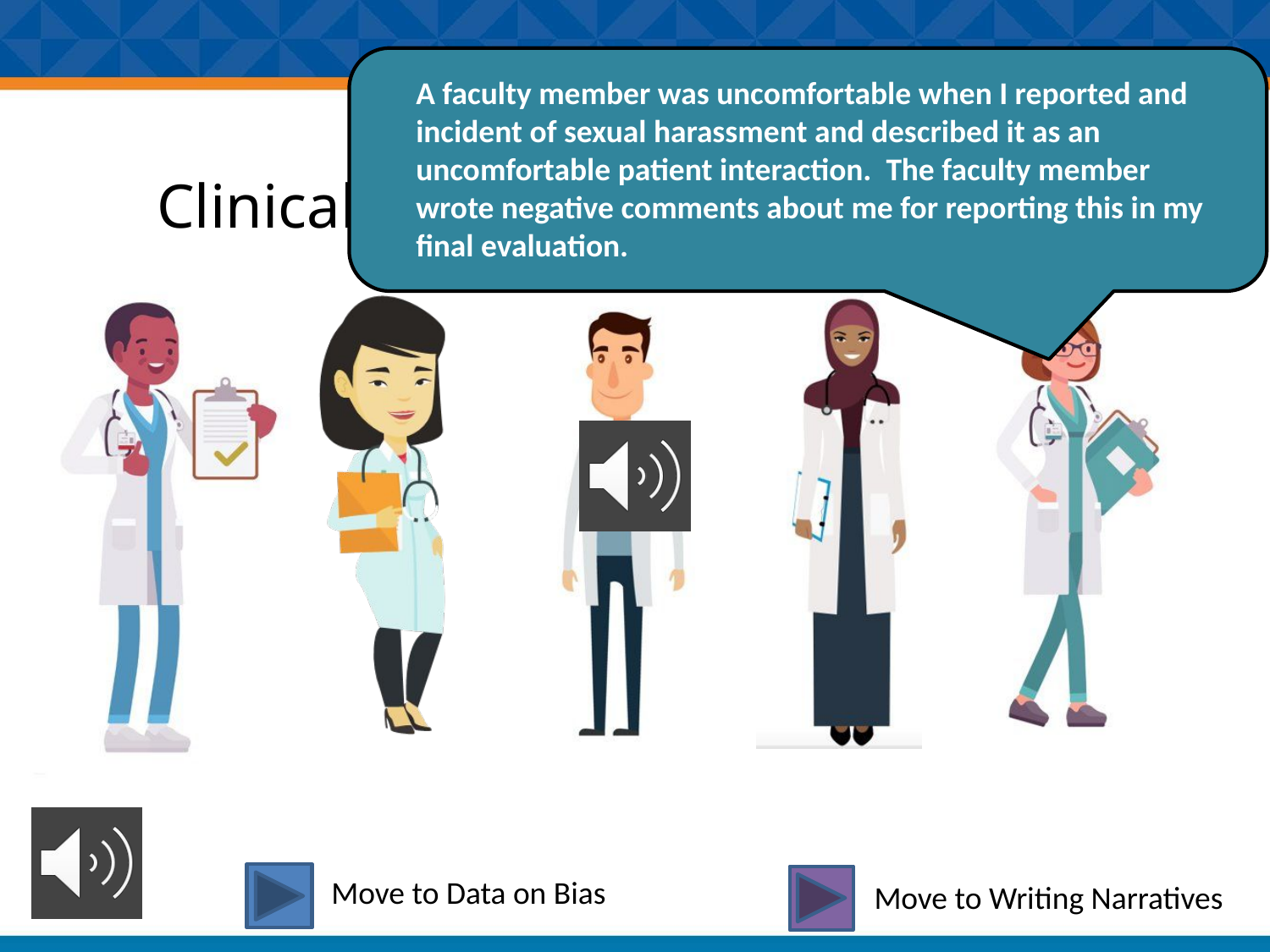

A faculty member was uncomfortable when I reported and incident of sexual harassment and described it as an uncomfortable patient interaction. The faculty member wrote negative comments about me for reporting this in my final evaluation.
# Bias in Clinical Education and Evaluations
Move to Data on Bias
Move to Writing Narratives

## Slide 12
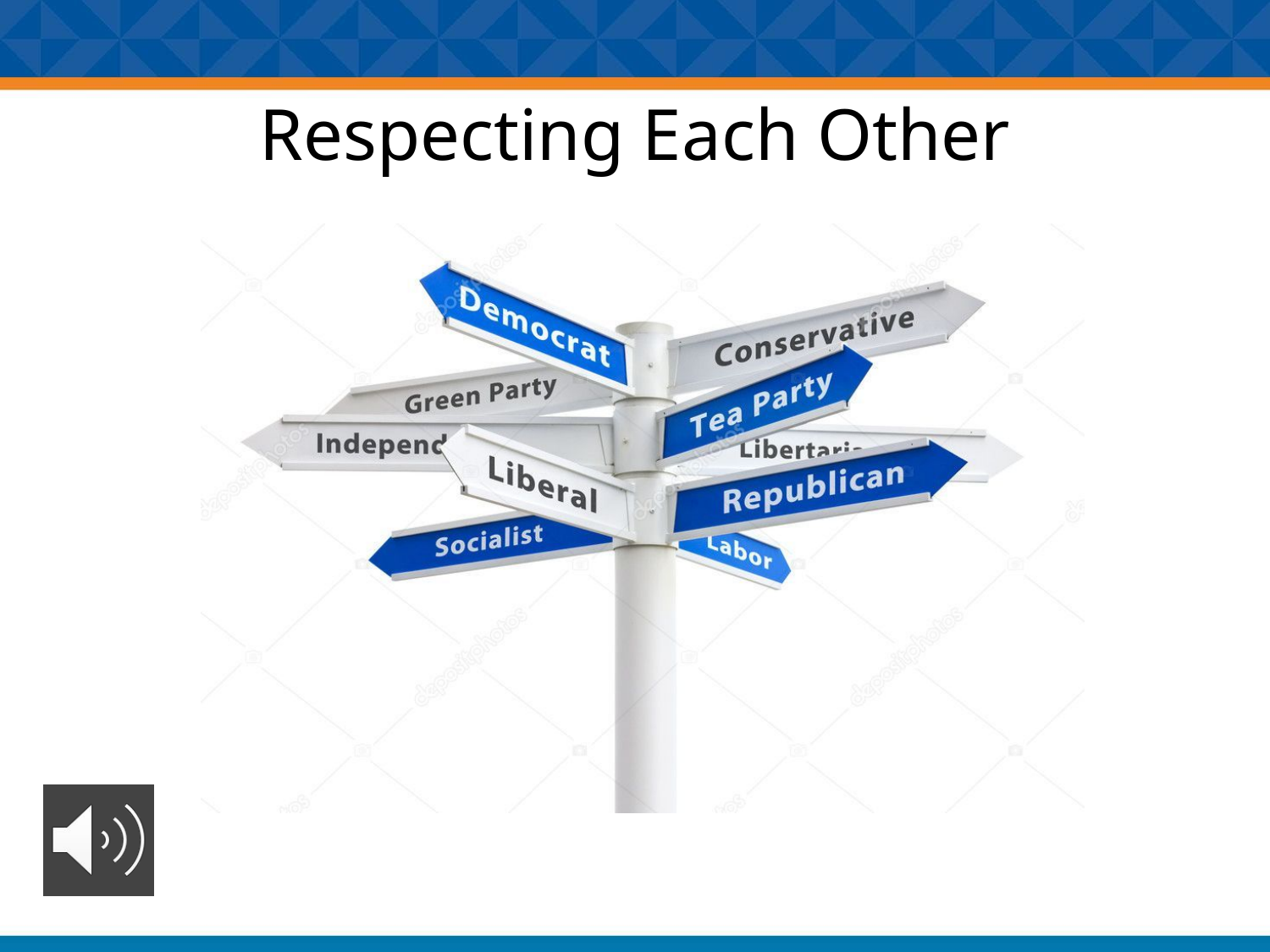

# Respecting Each Other

## Slide 13
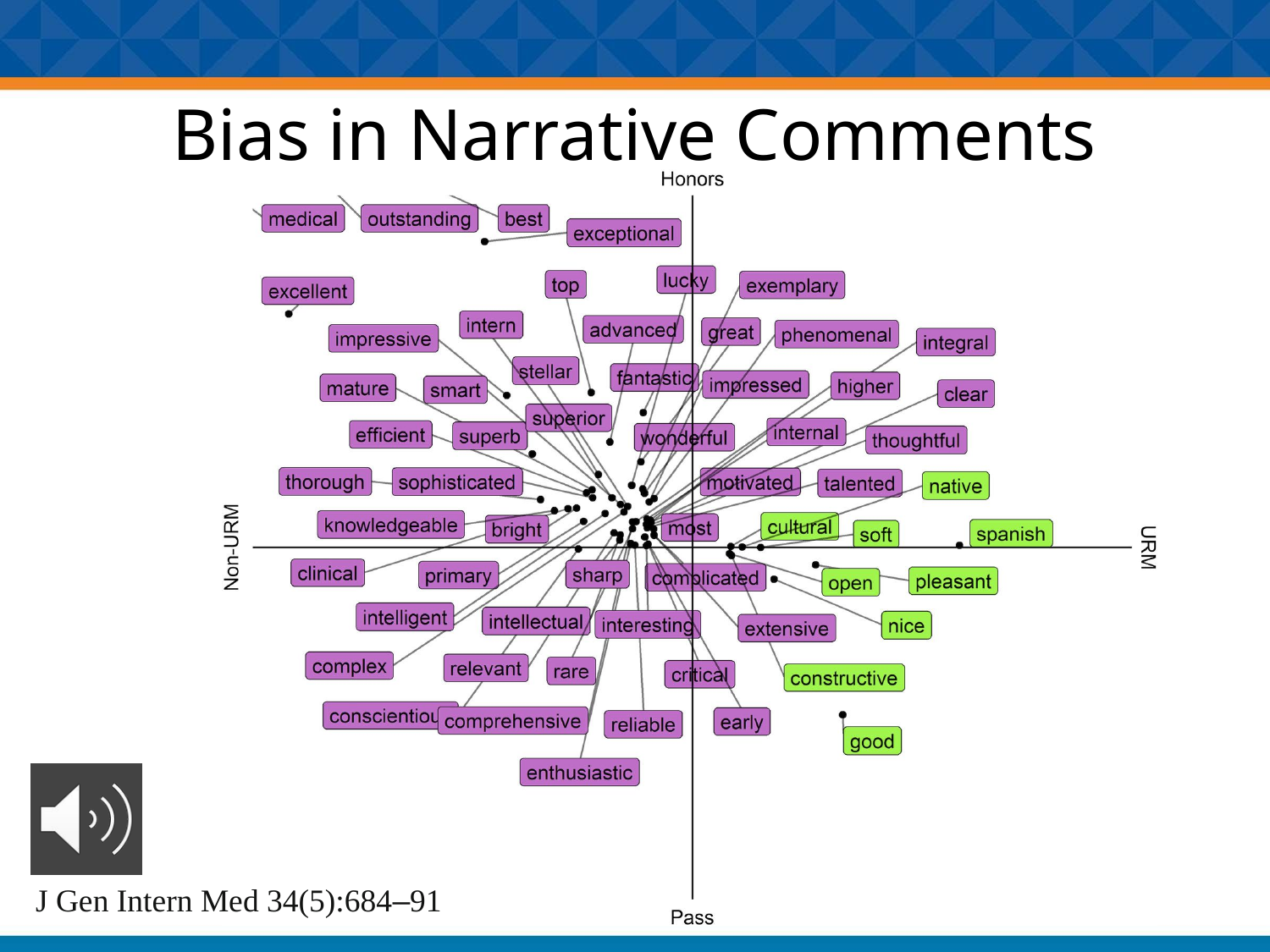

# Bias in Narrative Comments
J Gen Intern Med 34(5):684–91

## Slide 14
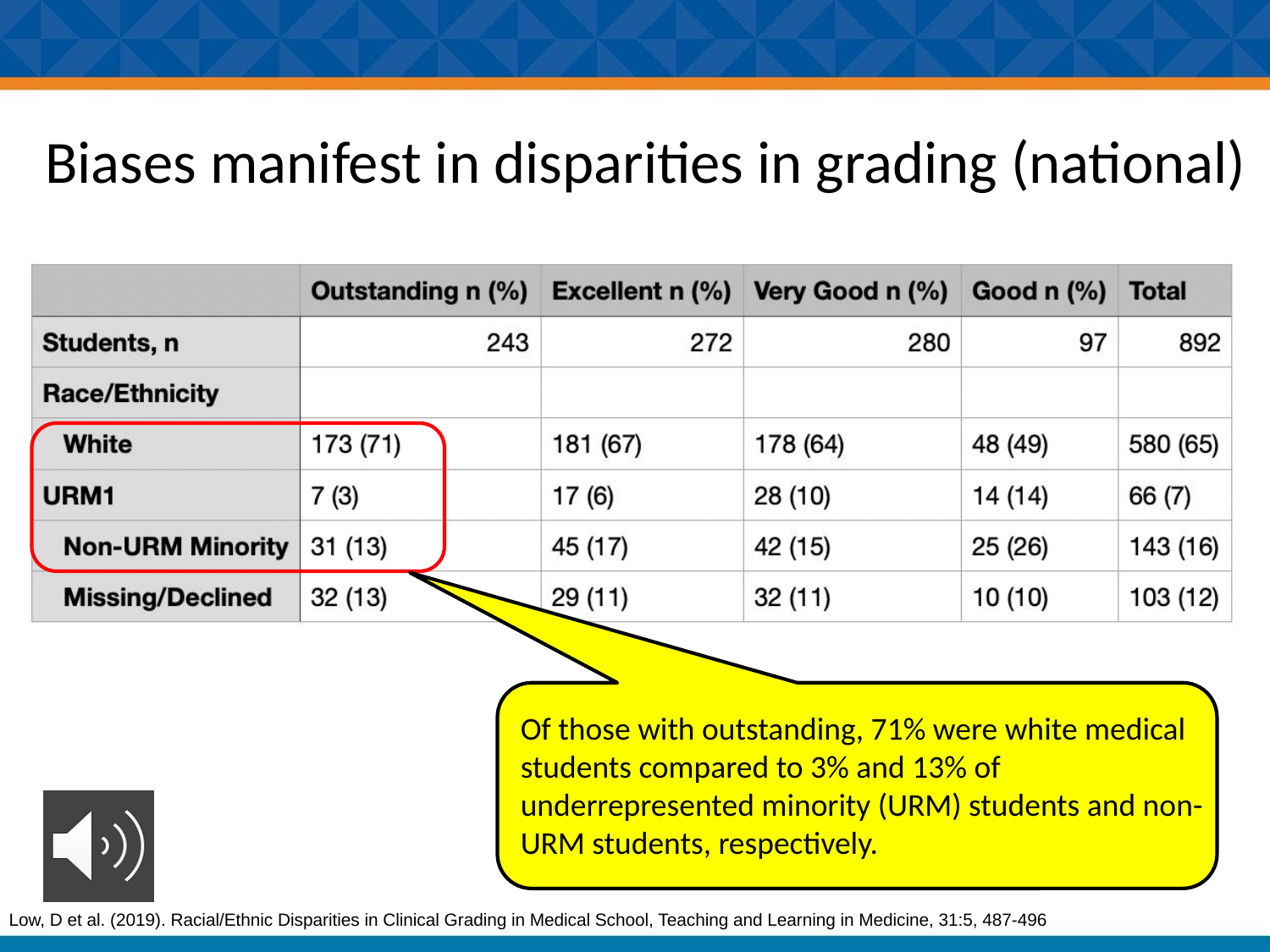

Biases manifest in disparities in grading (national)
Of those with outstanding, 71% were white medical students compared to 3% and 13% of underrepresented minority (URM) students and non-URM students, respectively.
Low, D et al. (2019). Racial/Ethnic Disparities in Clinical Grading in Medical School, Teaching and Learning in Medicine, 31:5, 487-496

## Slide 15
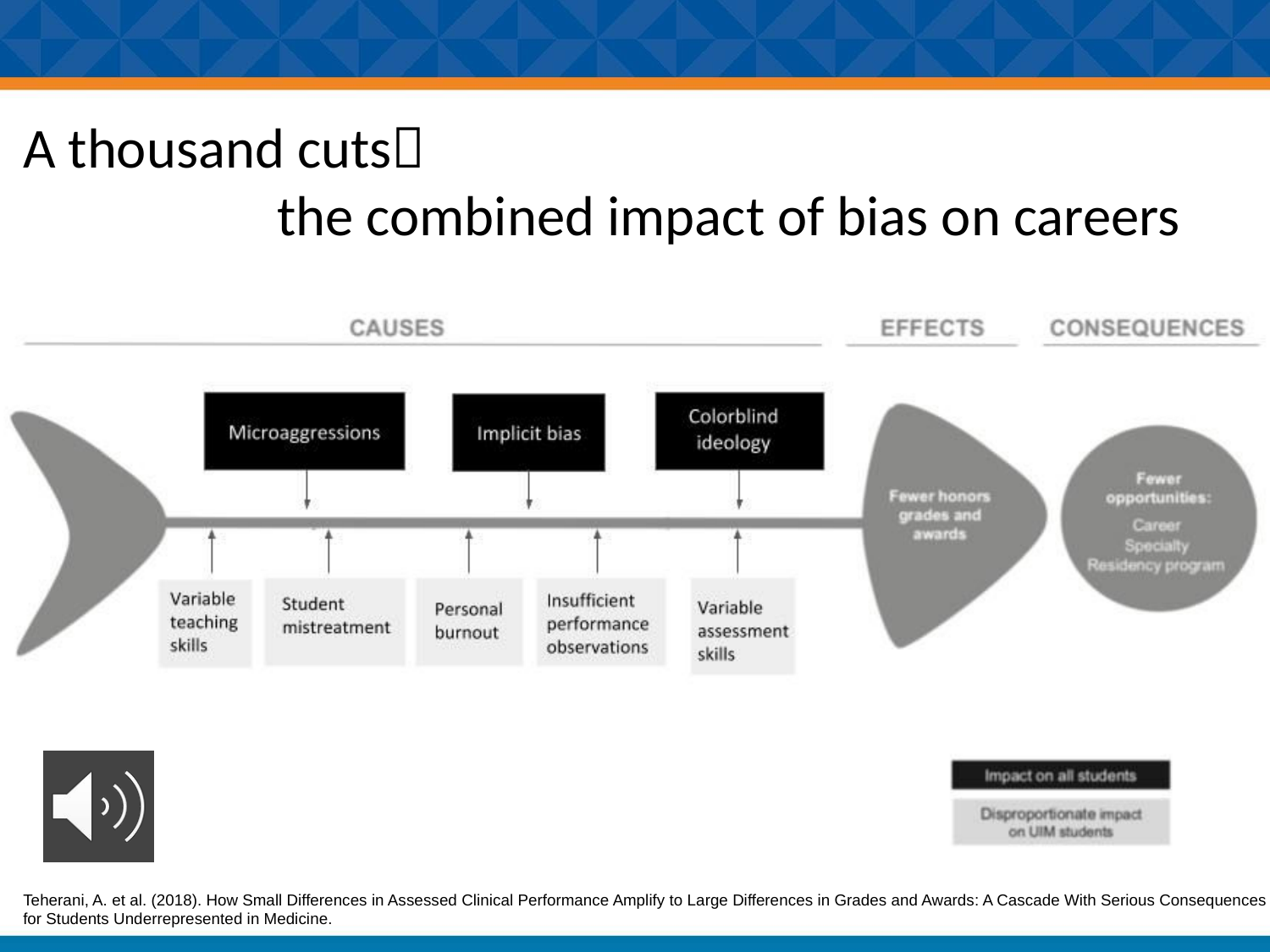

A thousand cuts
		the combined impact of bias on careers
Teherani, A. et al. (2018). How Small Differences in Assessed Clinical Performance Amplify to Large Differences in Grades and Awards: A Cascade With Serious Consequences for Students Underrepresented in Medicine.

## Slide 16
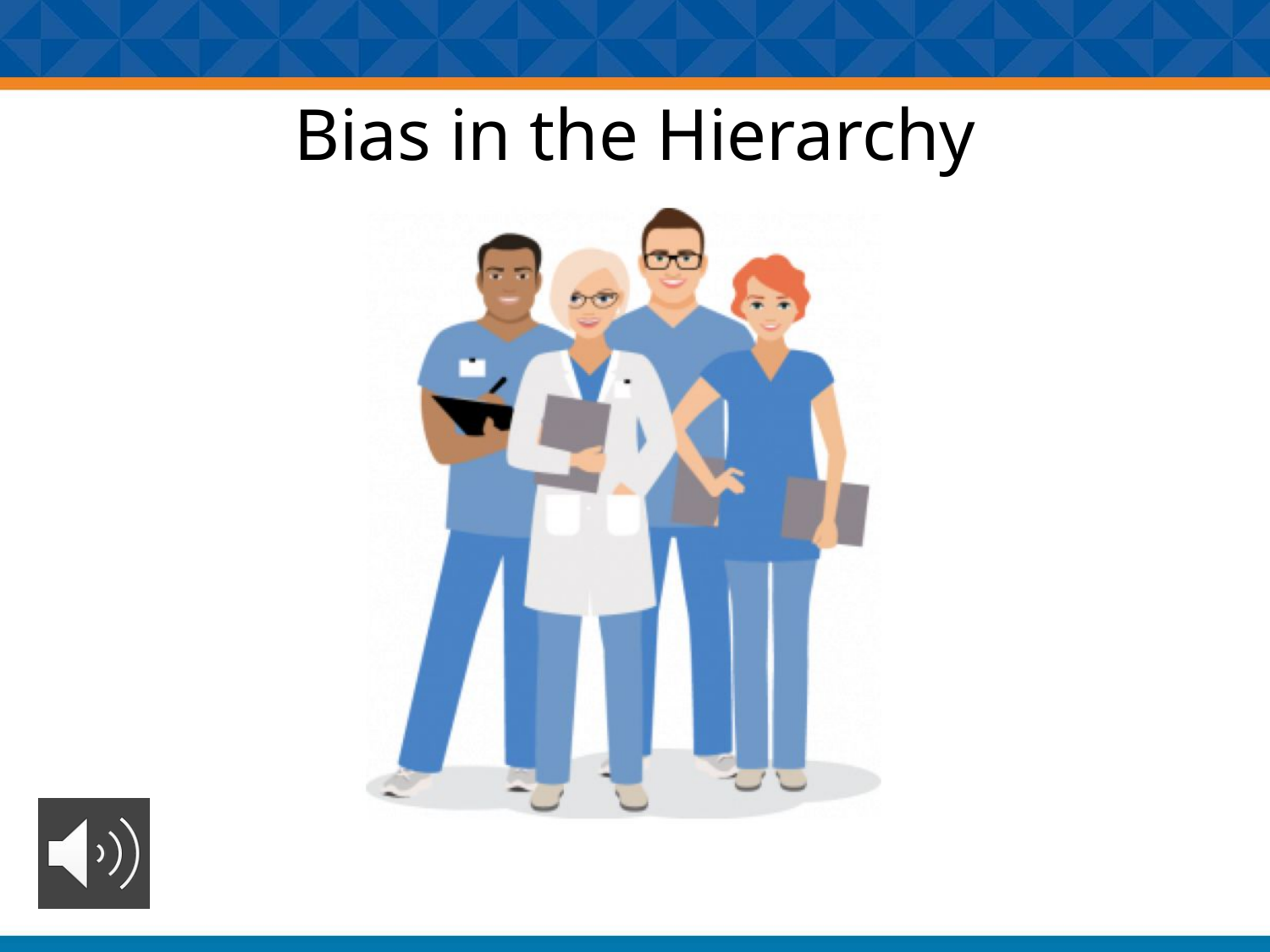

# Bias in the Hierarchy

## Slide 17
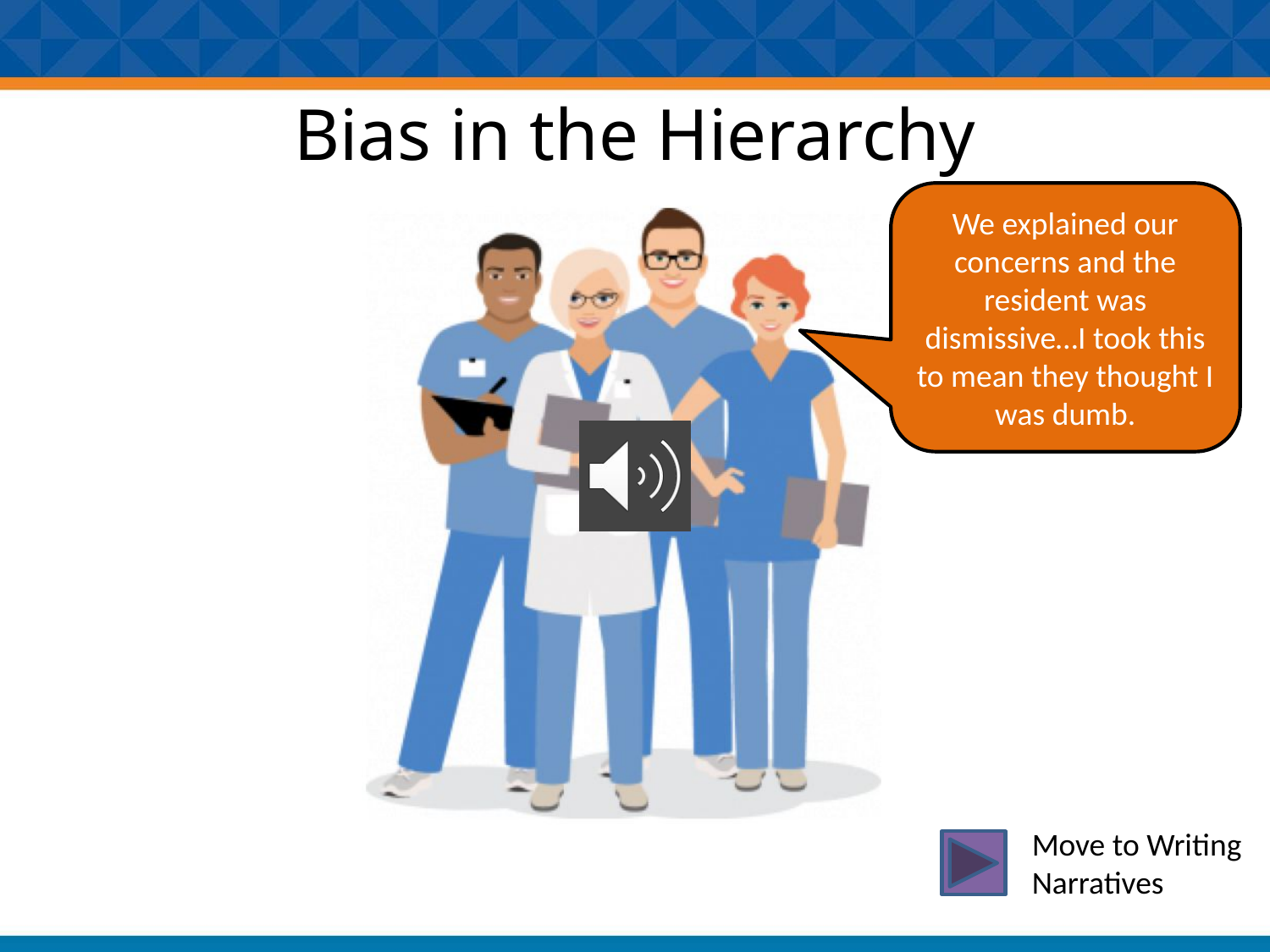

# Bias in the Hierarchy
We explained our concerns and the resident was dismissive…I took this to mean they thought I was dumb.
Move to Writing Narratives

## Slide 18
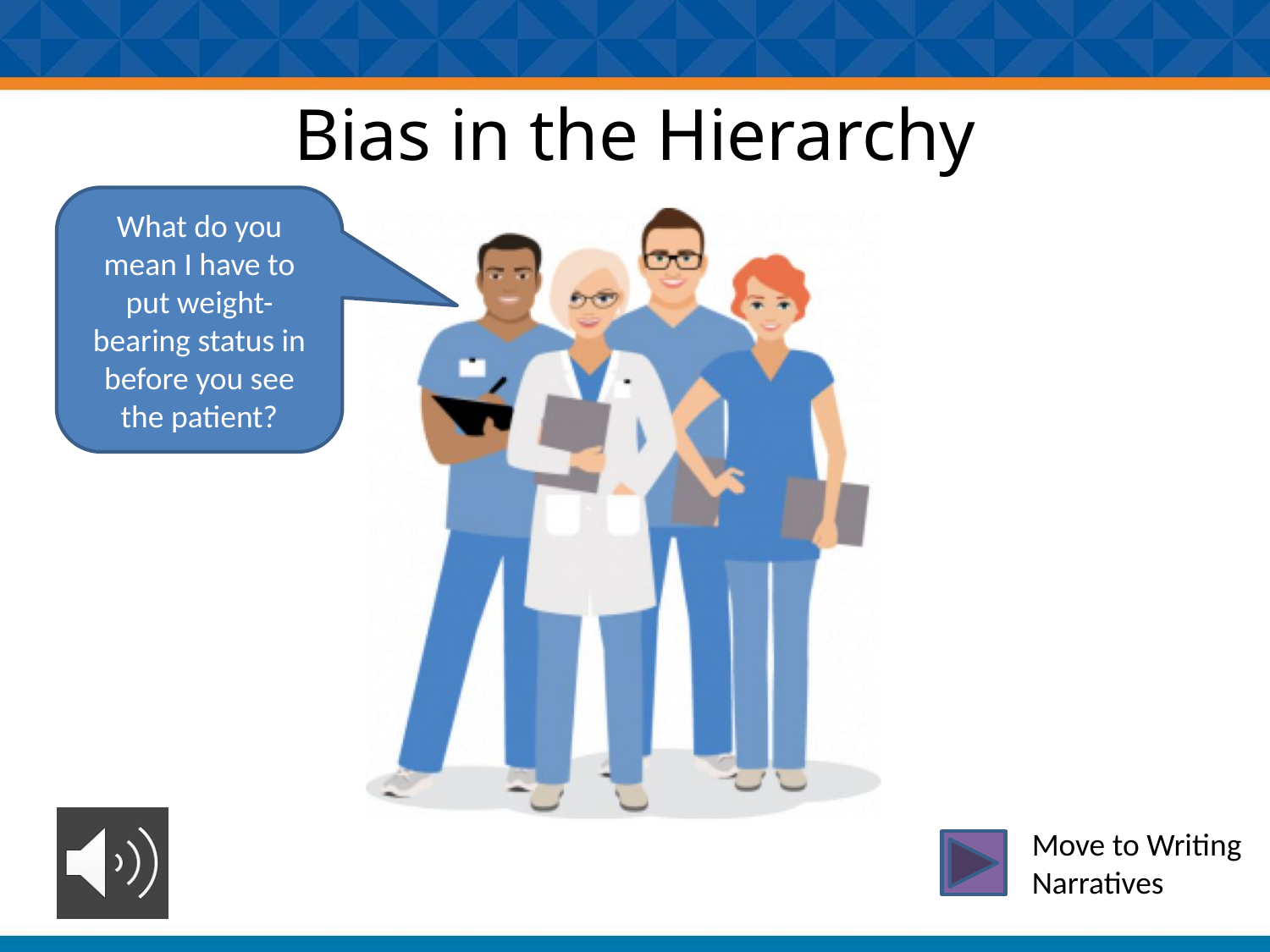

# Bias in the Hierarchy
What do you mean I have to put weight-bearing status in before you see the patient?
Move to Writing Narratives

## Slide 19
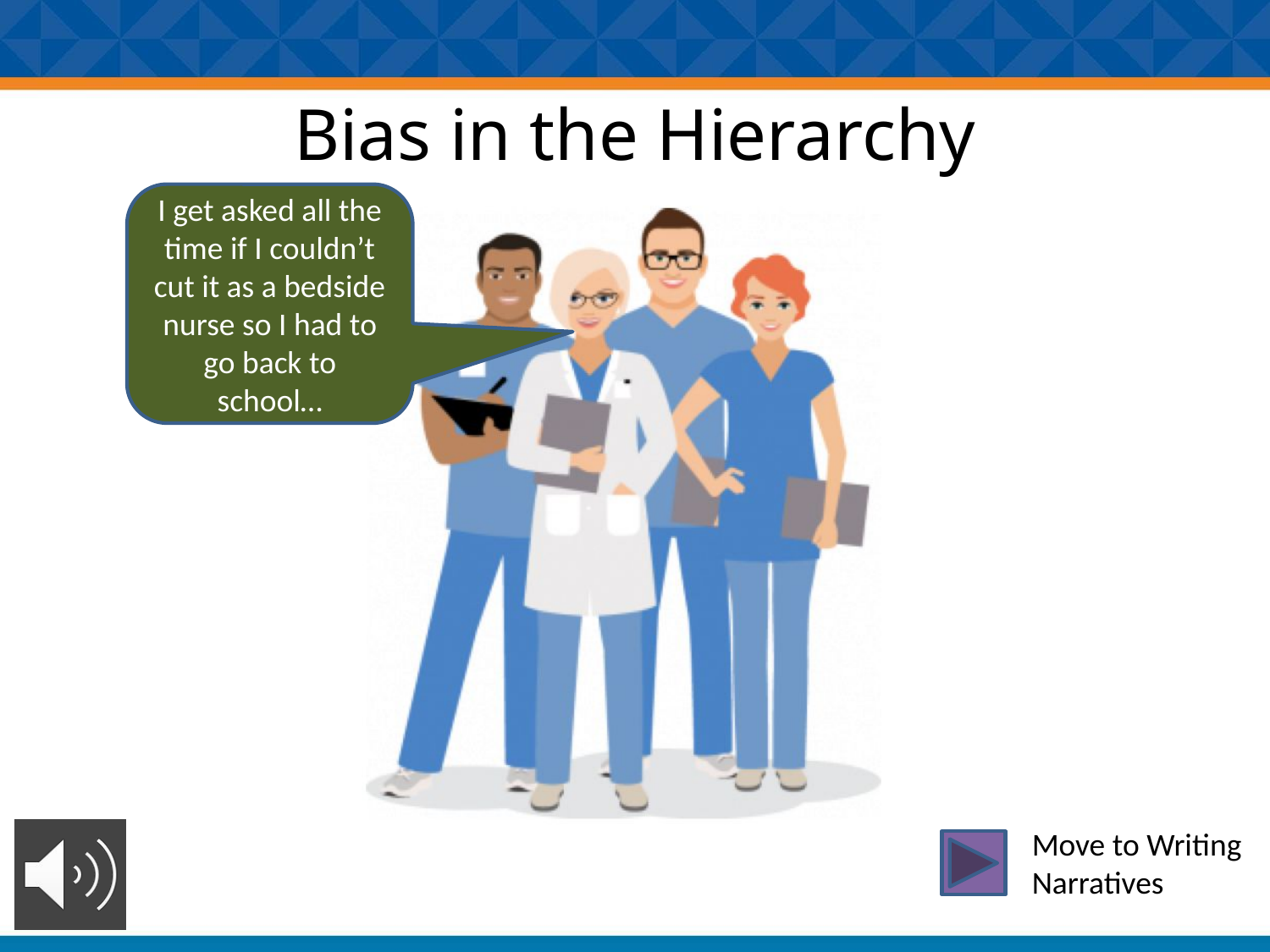

# Bias in the Hierarchy
I get asked all the time if I couldn’t cut it as a bedside nurse so I had to go back to school…
Move to Writing Narratives

## Slide 20
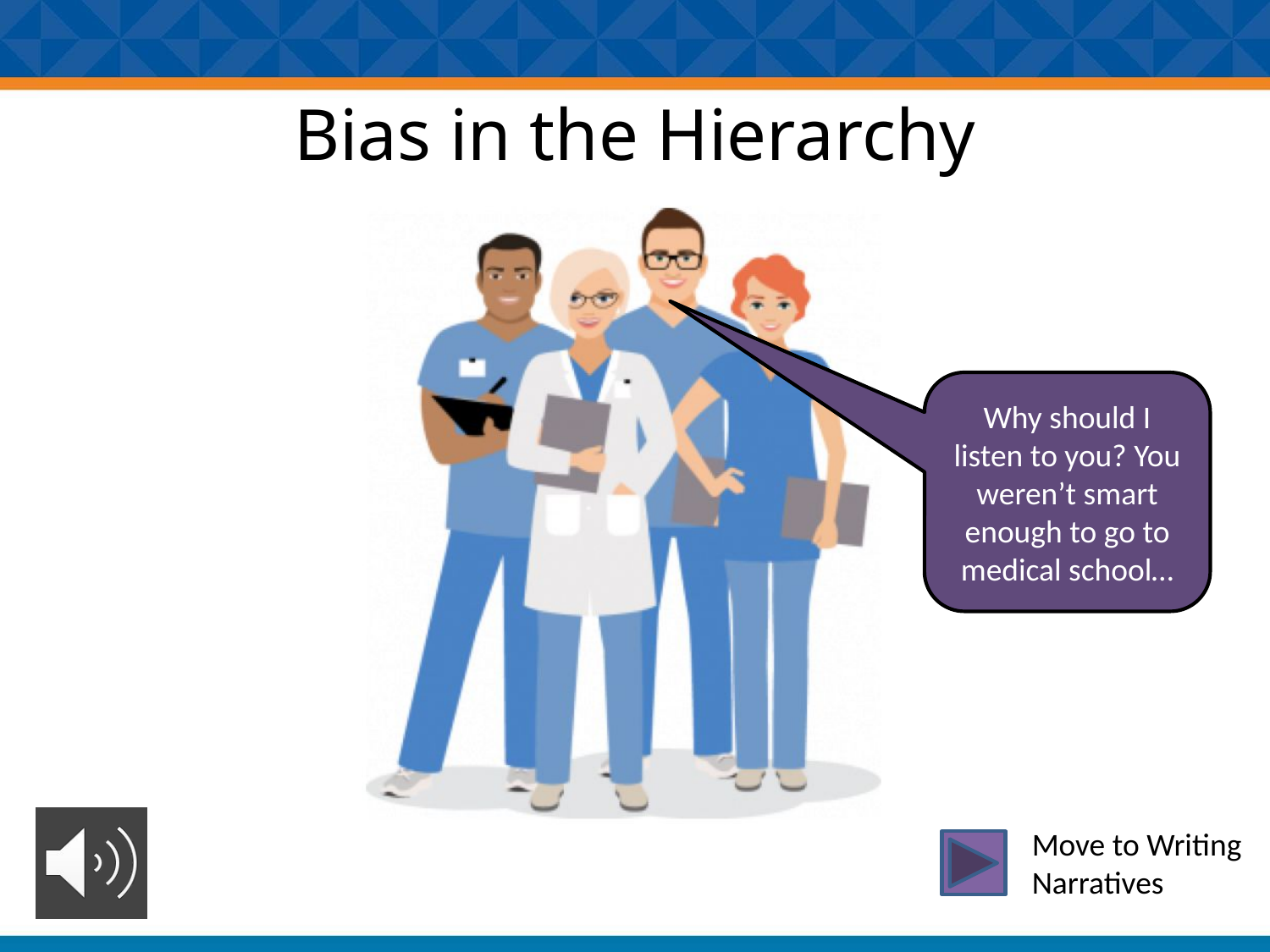

# Bias in the Hierarchy
Why should I listen to you? You weren’t smart enough to go to medical school…
Move to Writing Narratives

## Slide 21
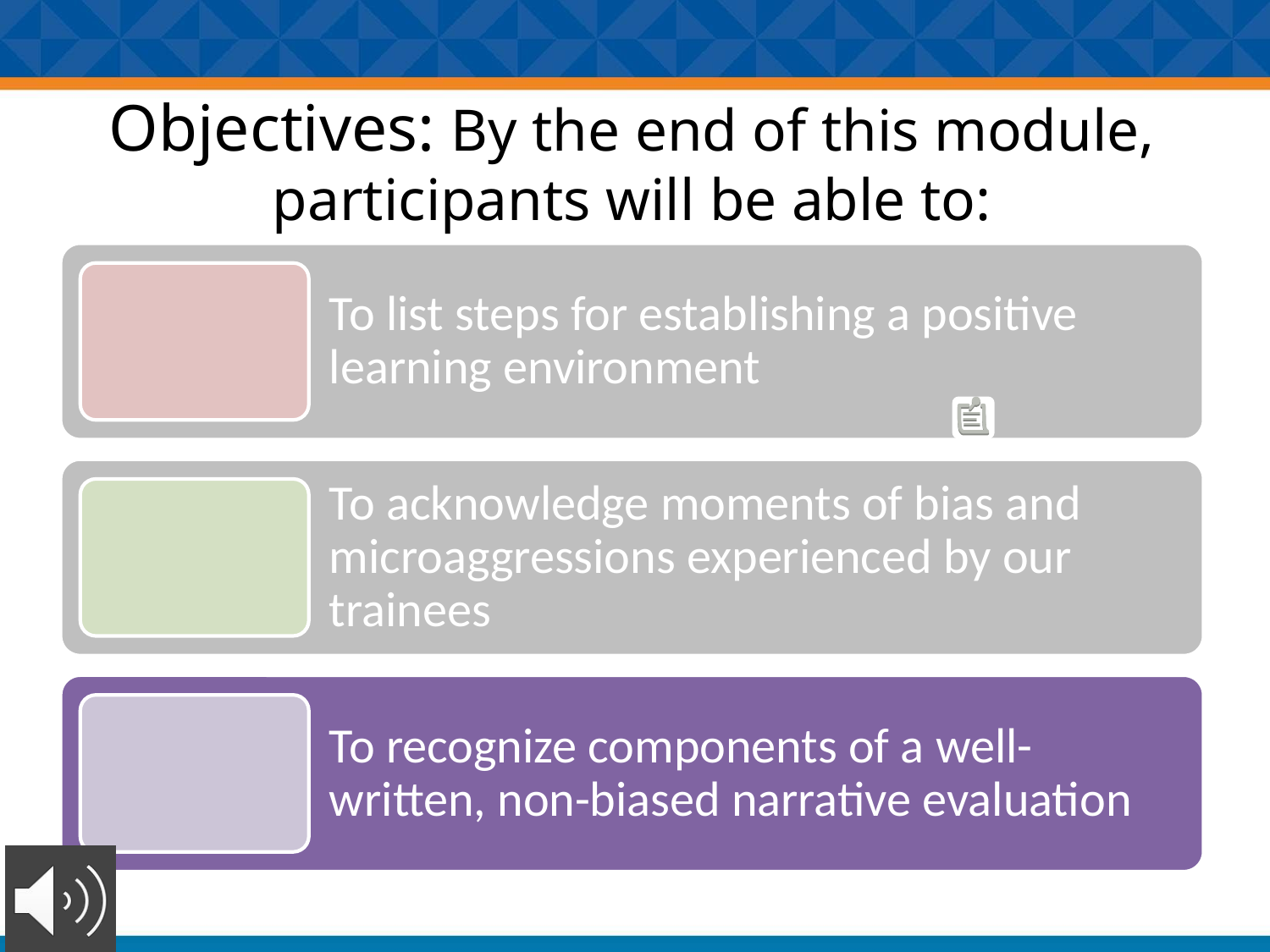

# Objectives: By the end of this module, participants will be able to:

## Slide 22
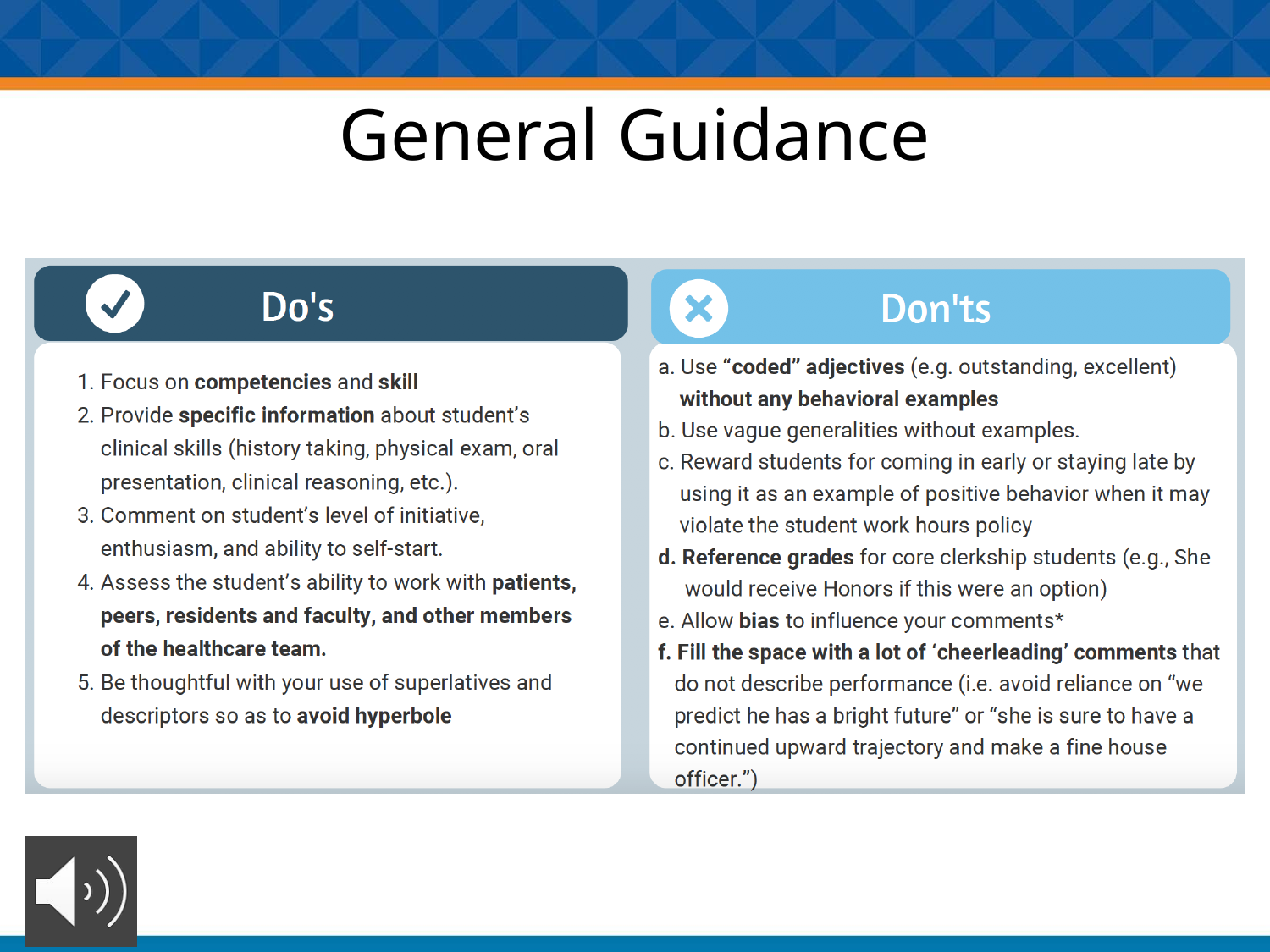

# General Guidance

## Slide 23
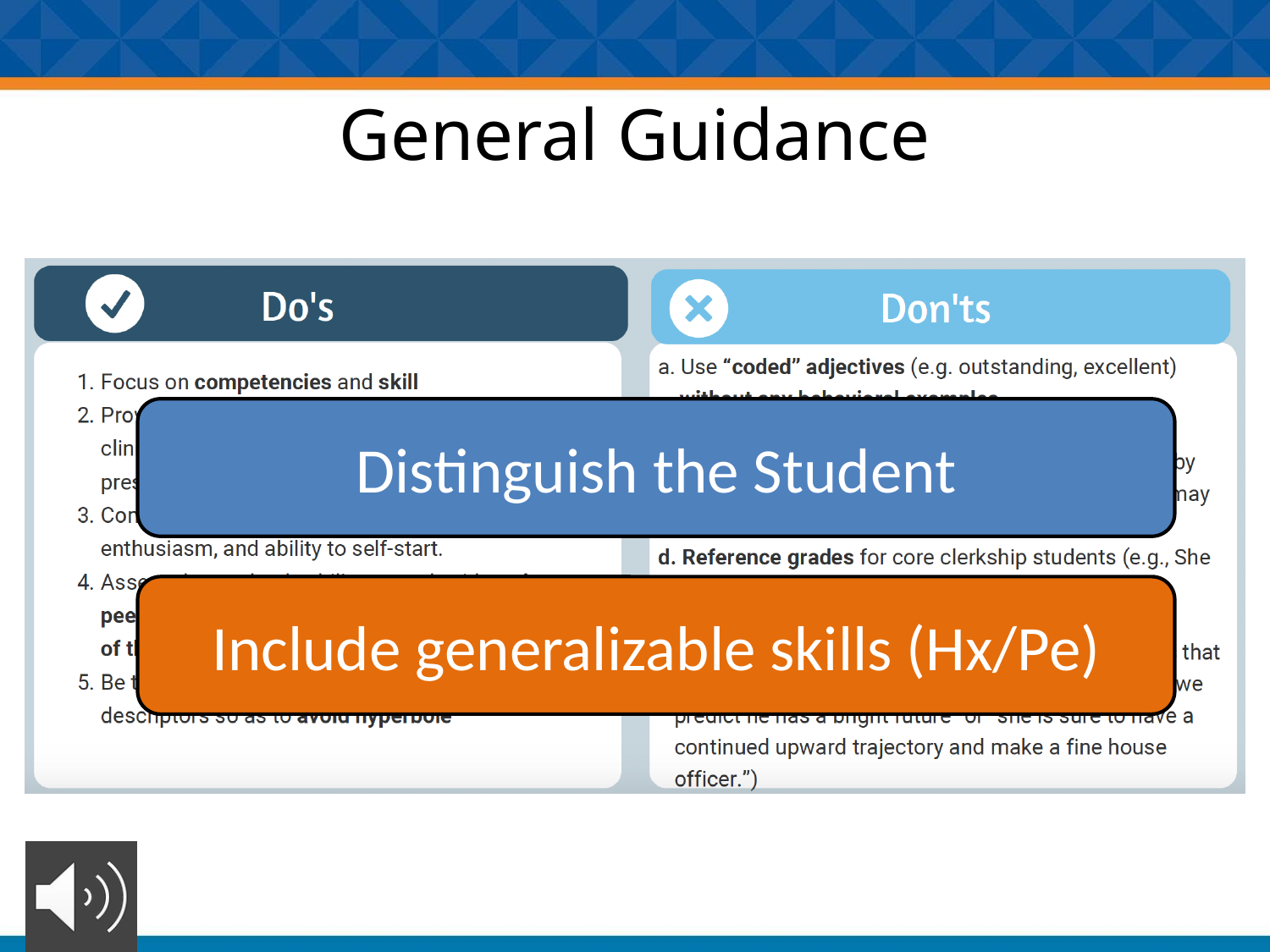

# General Guidance
Distinguish the Student
Include generalizable skills (Hx/Pe)

## Slide 24
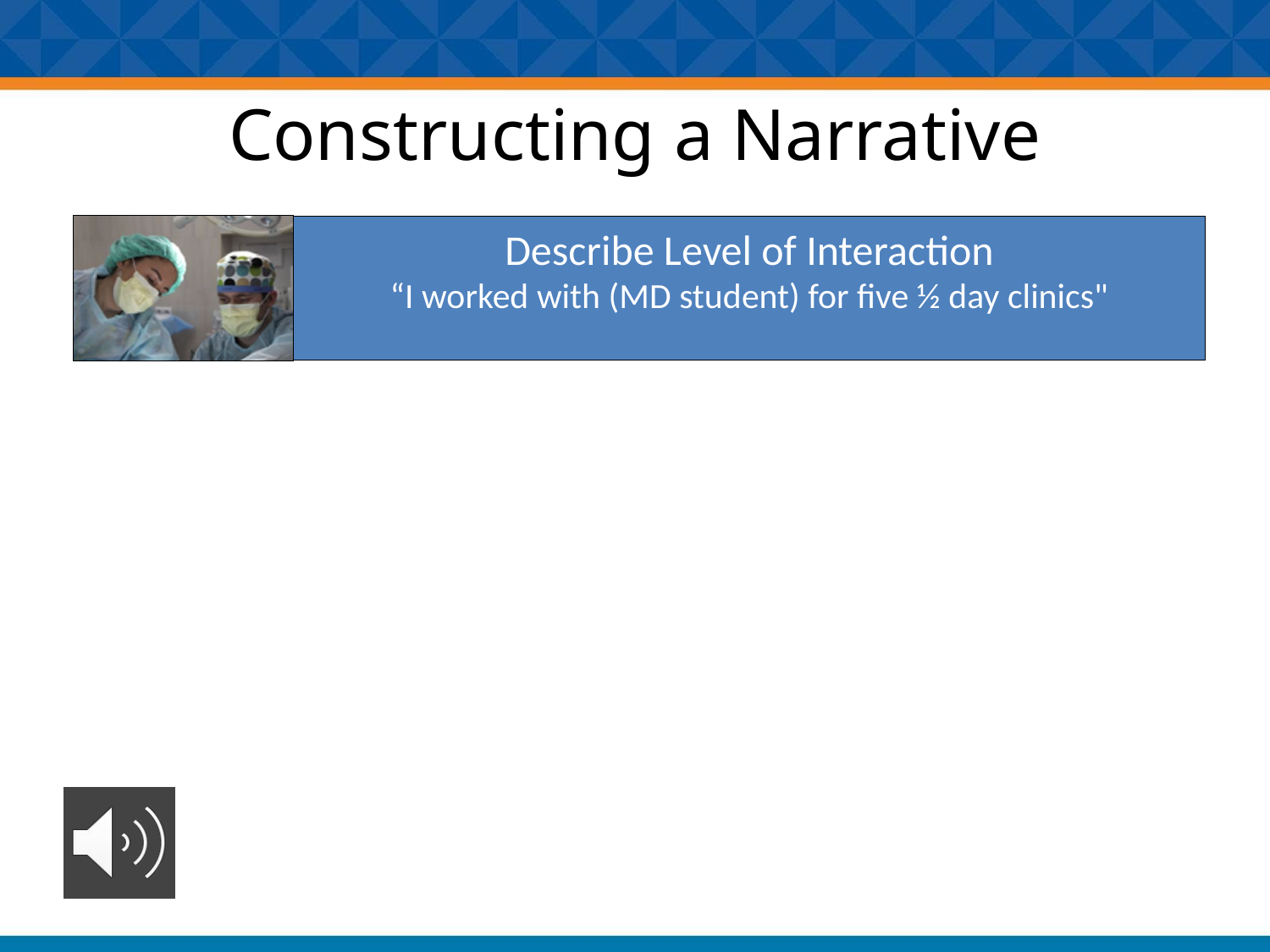

# Constructing a Narrative
Describe Level of Interaction
“I worked with (MD student) for five ½ day clinics"

## Slide 25
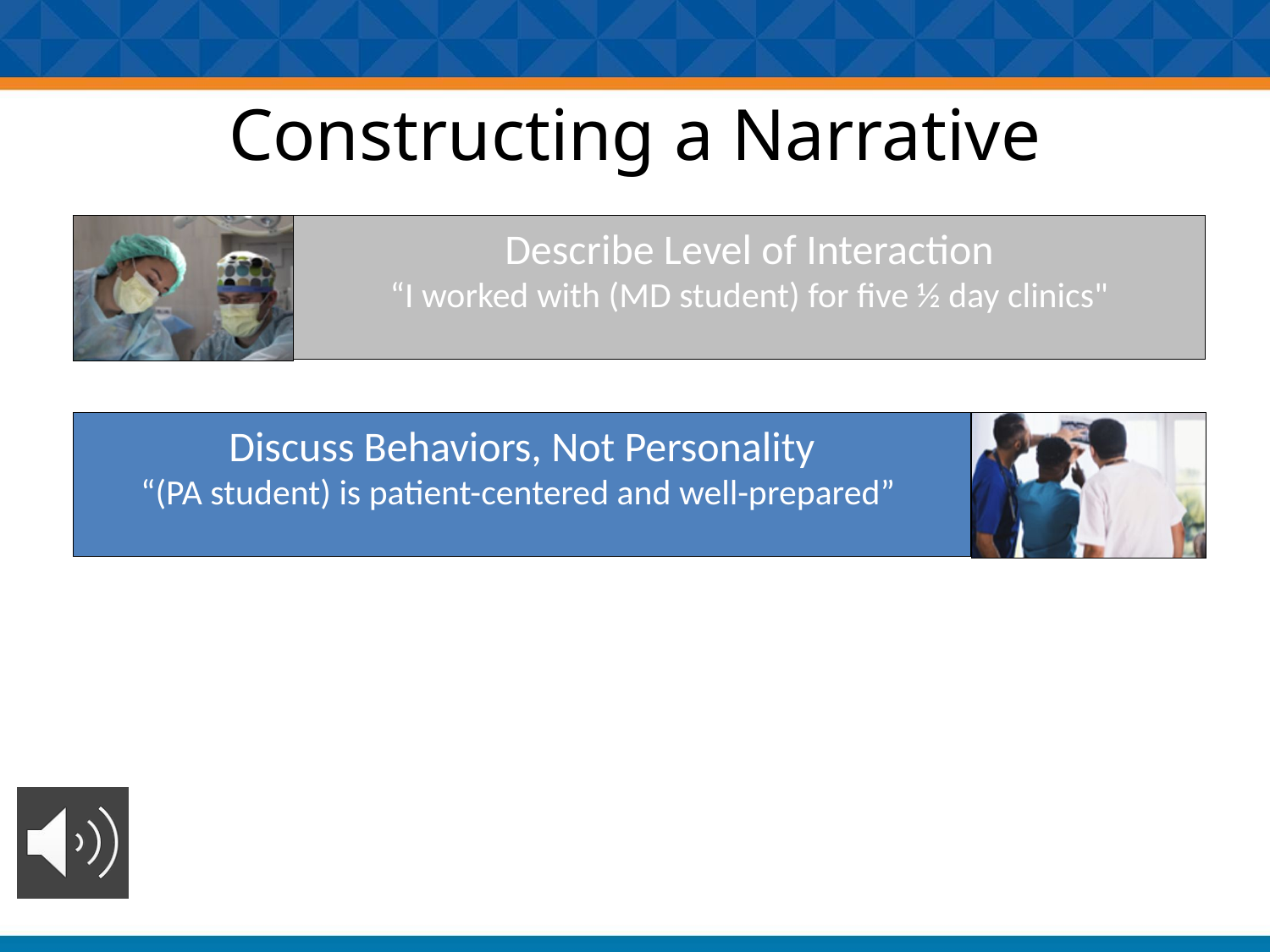

# Constructing a Narrative
Describe Level of Interaction
“I worked with (MD student) for five ½ day clinics"
Discuss Behaviors, Not Personality
“(PA student) is patient-centered and well-prepared”

## Slide 26
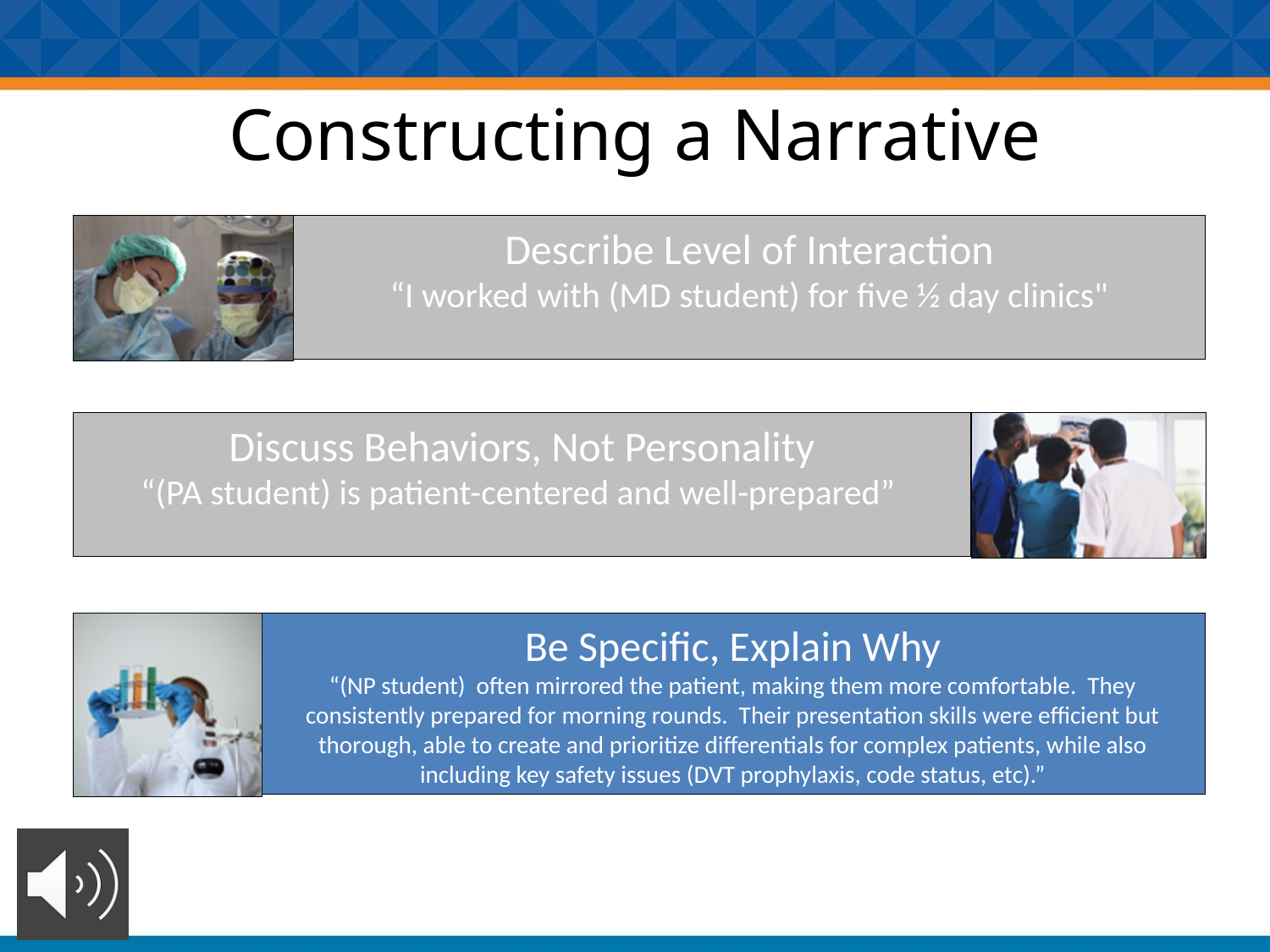

# Constructing a Narrative
Describe Level of Interaction
“I worked with (MD student) for five ½ day clinics"
Discuss Behaviors, Not Personality
“(PA student) is patient-centered and well-prepared”
Be Specific, Explain Why
“(NP student) often mirrored the patient, making them more comfortable. They consistently prepared for morning rounds. Their presentation skills were efficient but thorough, able to create and prioritize differentials for complex patients, while also including key safety issues (DVT prophylaxis, code status, etc).”

## Slide 27
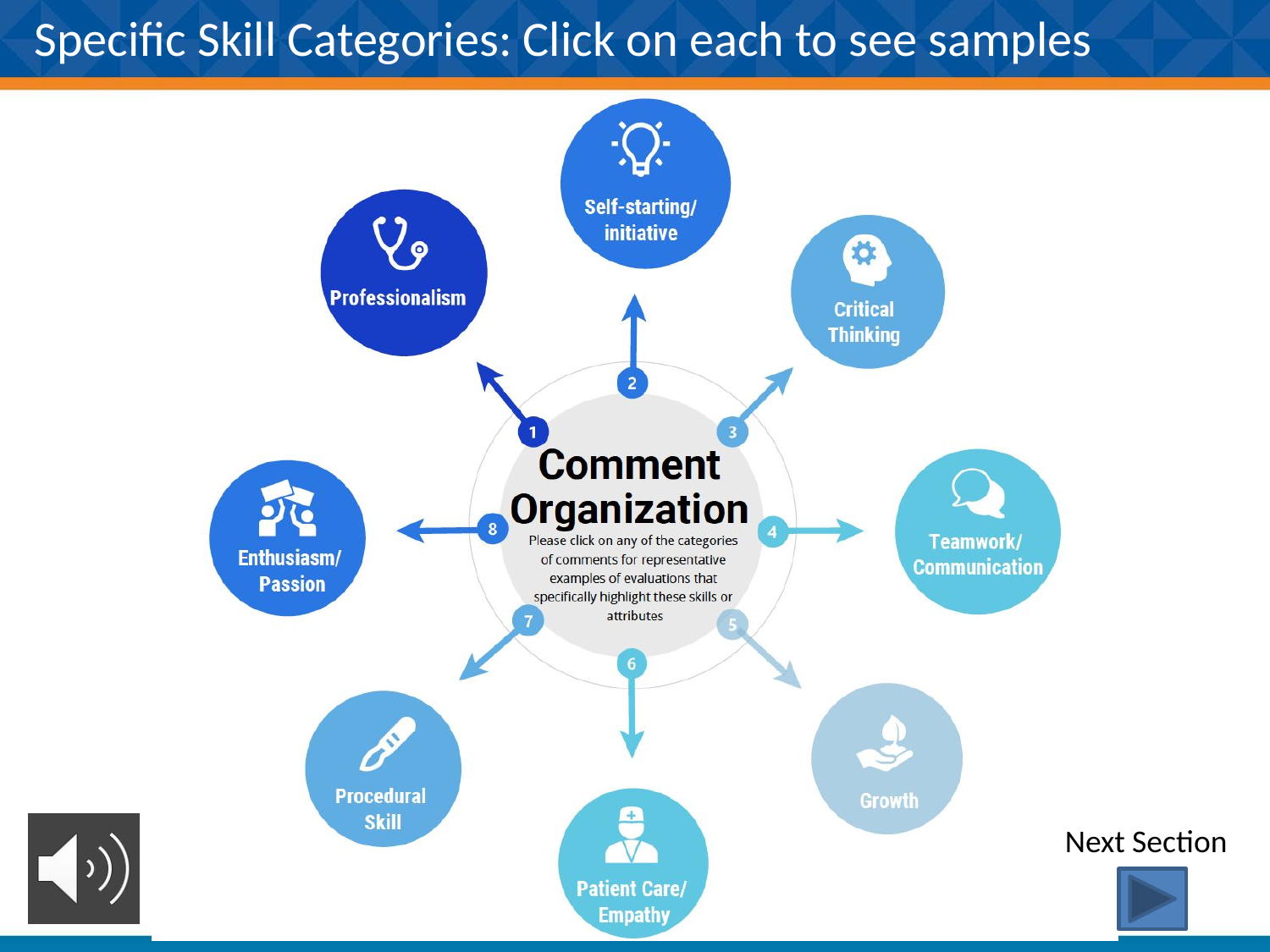

Specific Skill Categories: Click on each to see samples
Next Section

## Slide 28
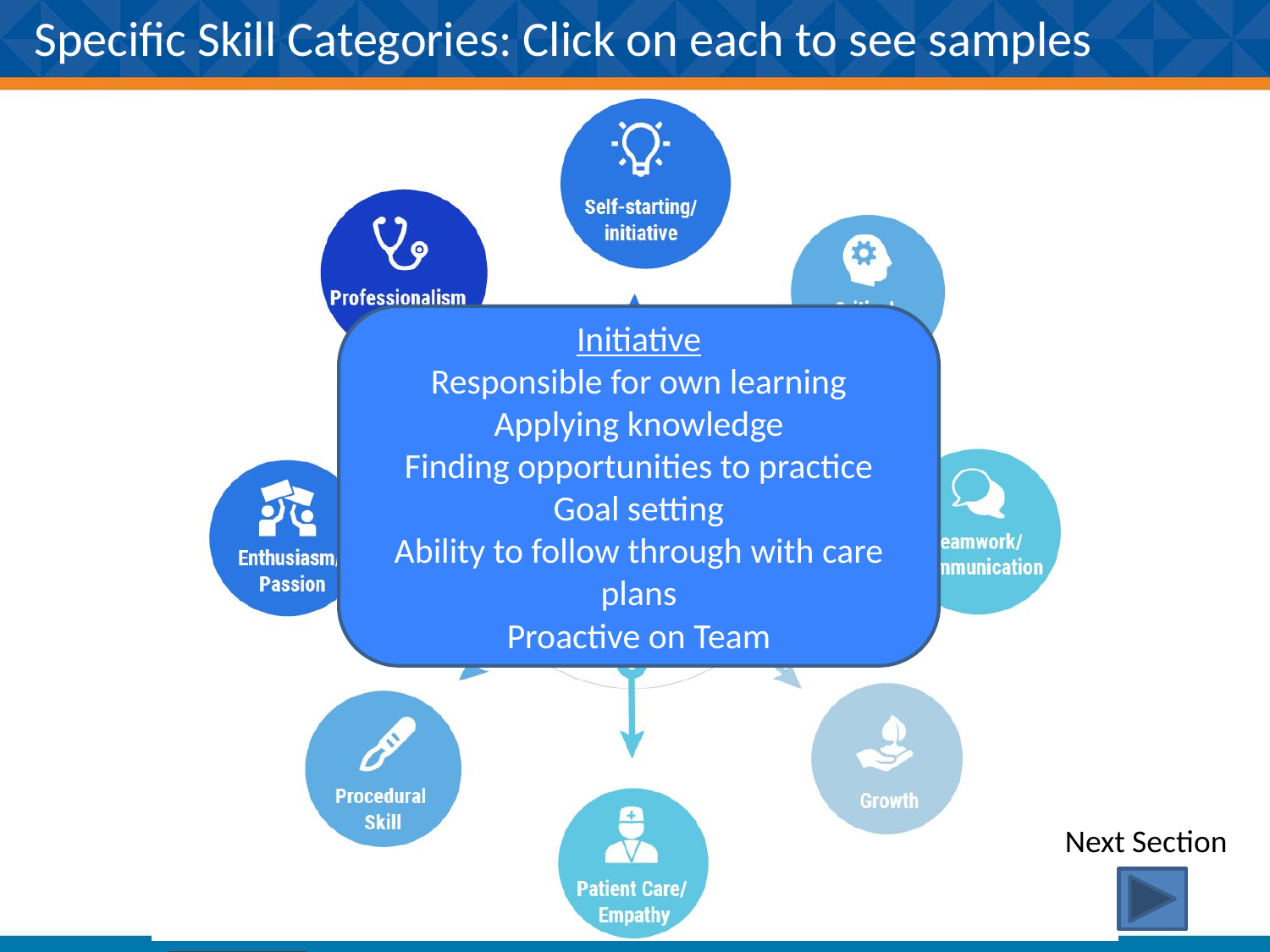

Specific Skill Categories: Click on each to see samples
Initiative
Responsible for own learning
Applying knowledge
Finding opportunities to practice
Goal setting
Ability to follow through with care plans
Proactive on Team
Next Section

## Slide 29
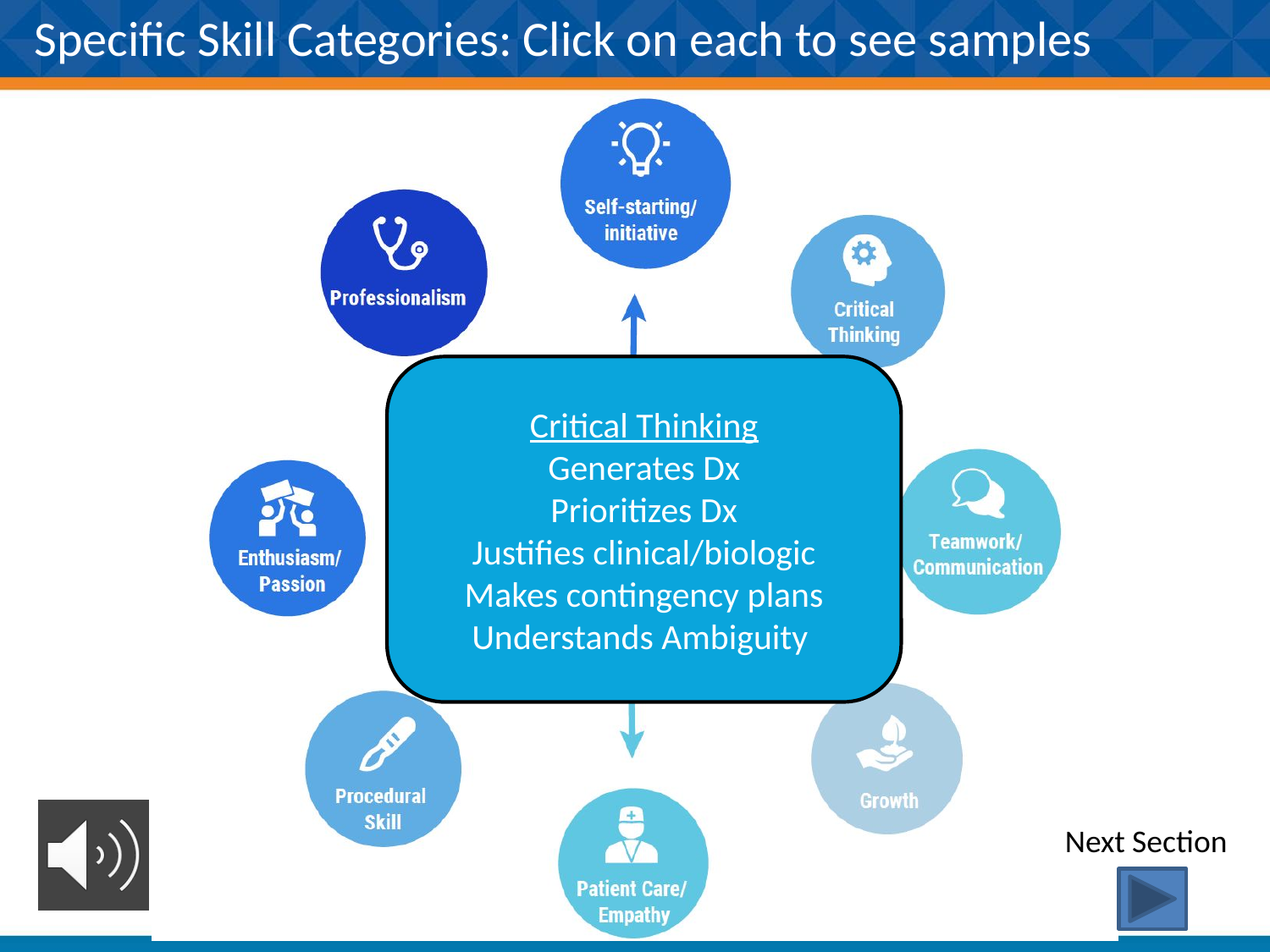

Specific Skill Categories: Click on each to see samples
Critical Thinking
Generates Dx
Prioritizes Dx
Justifies clinical/biologic
Makes contingency plans
Understands Ambiguity
Next Section

## Slide 30
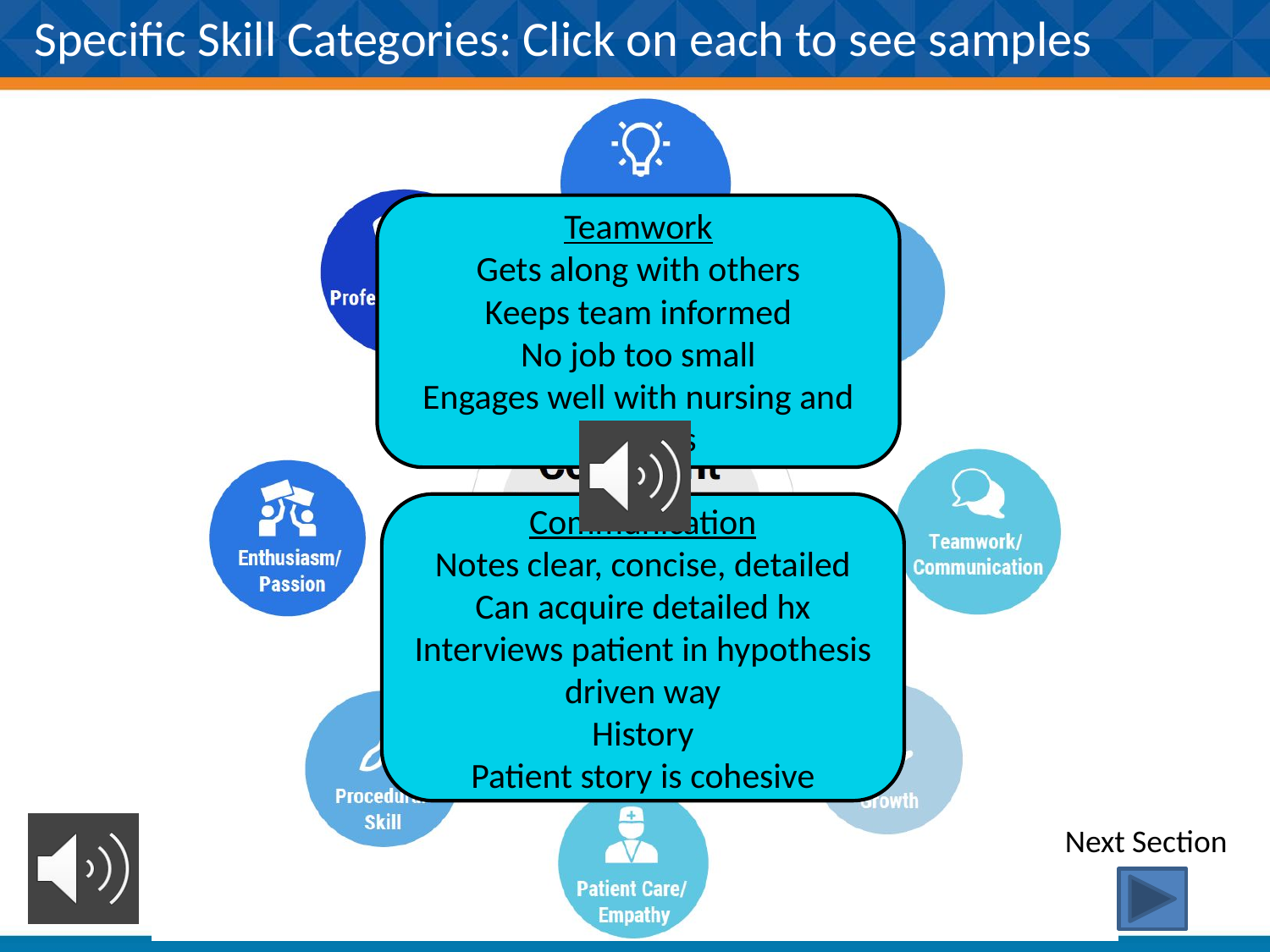

Specific Skill Categories: Click on each to see samples
Teamwork
Gets along with others
Keeps team informed
No job too small
Engages well with nursing and patients
Communication
Notes clear, concise, detailed
Can acquire detailed hx
Interviews patient in hypothesis driven way
History
Patient story is cohesive
Next Section

## Slide 31
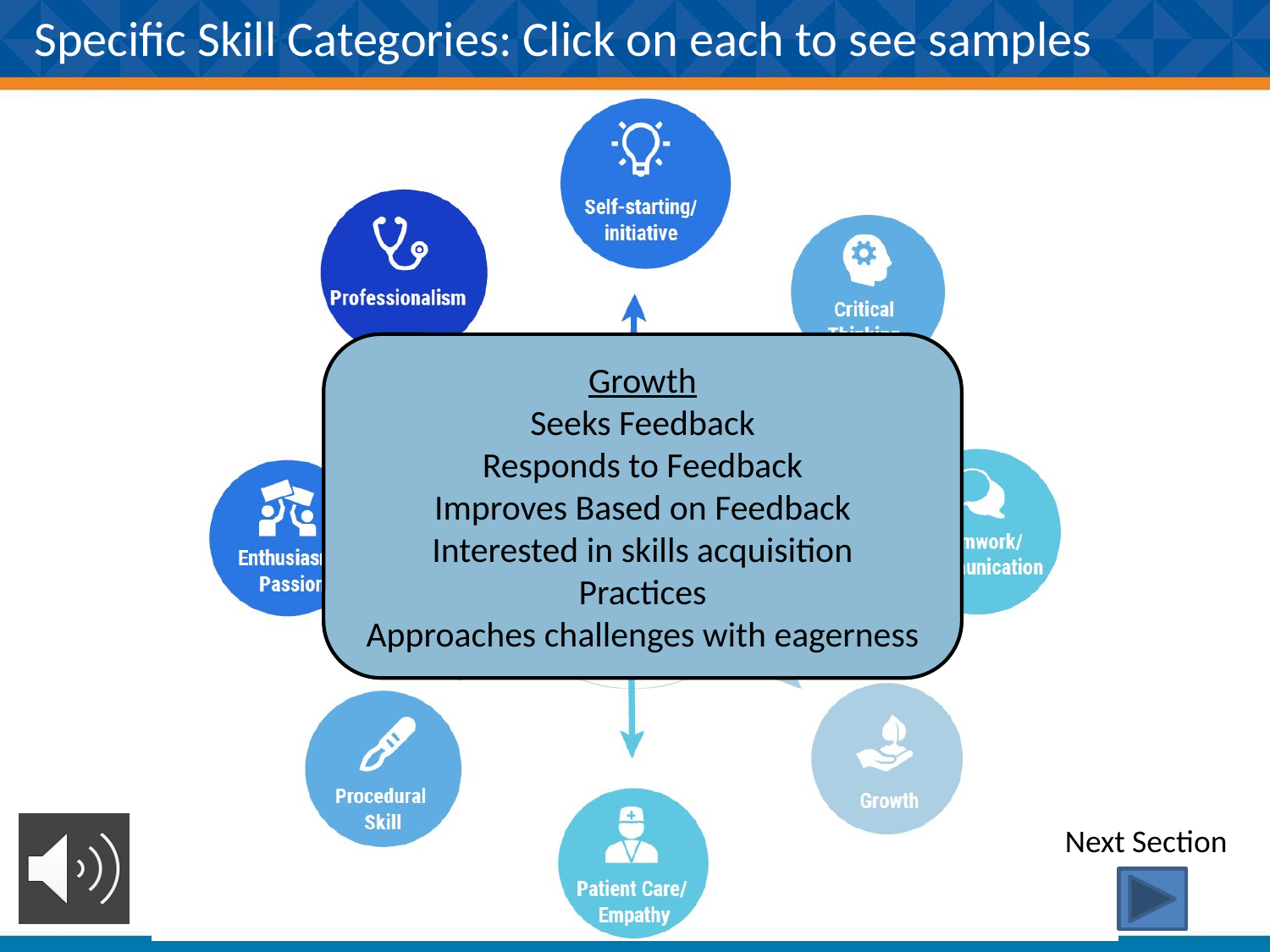

Specific Skill Categories: Click on each to see samples
Growth
Seeks Feedback
Responds to Feedback
Improves Based on Feedback
Interested in skills acquisition
Practices
Approaches challenges with eagerness
Next Section

## Slide 32
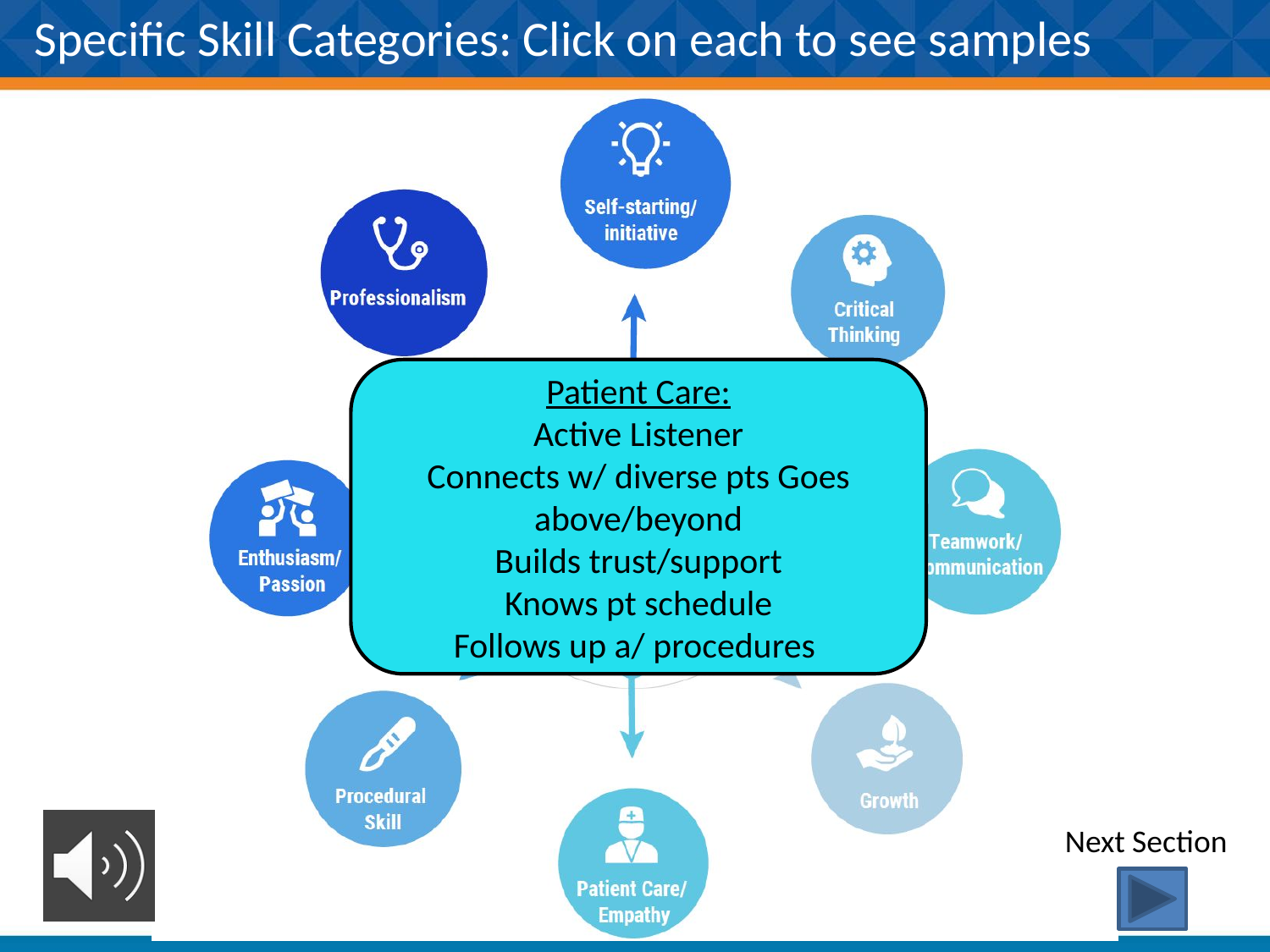

Specific Skill Categories: Click on each to see samples
Patient Care:
Active Listener
Connects w/ diverse pts Goes above/beyond
Builds trust/support
Knows pt schedule
Follows up a/ procedures
Next Section

## Slide 33
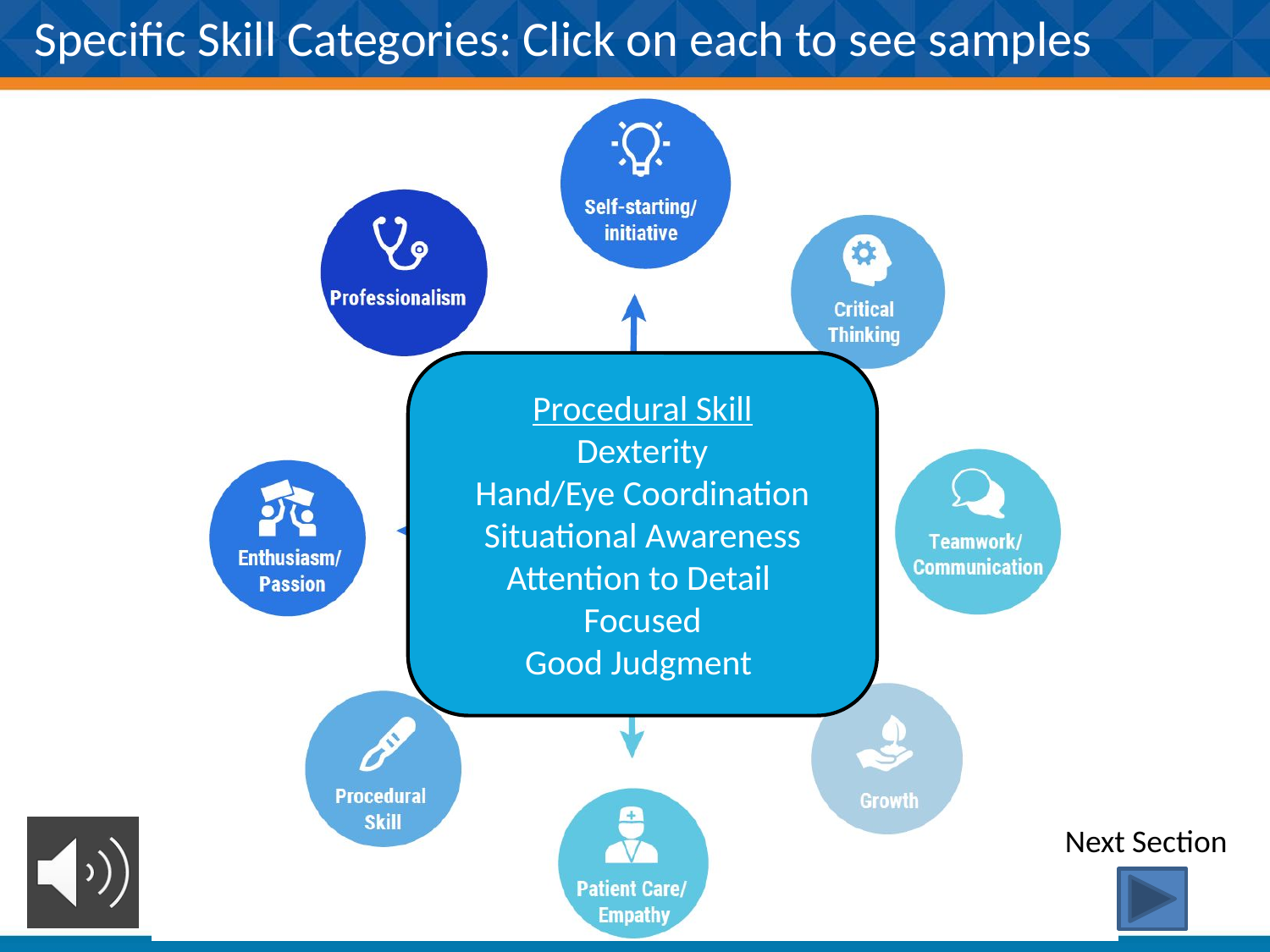

Specific Skill Categories: Click on each to see samples
Procedural Skill
Dexterity
Hand/Eye Coordination
Situational Awareness
Attention to Detail
Focused
Good Judgment
Next Section

## Slide 34
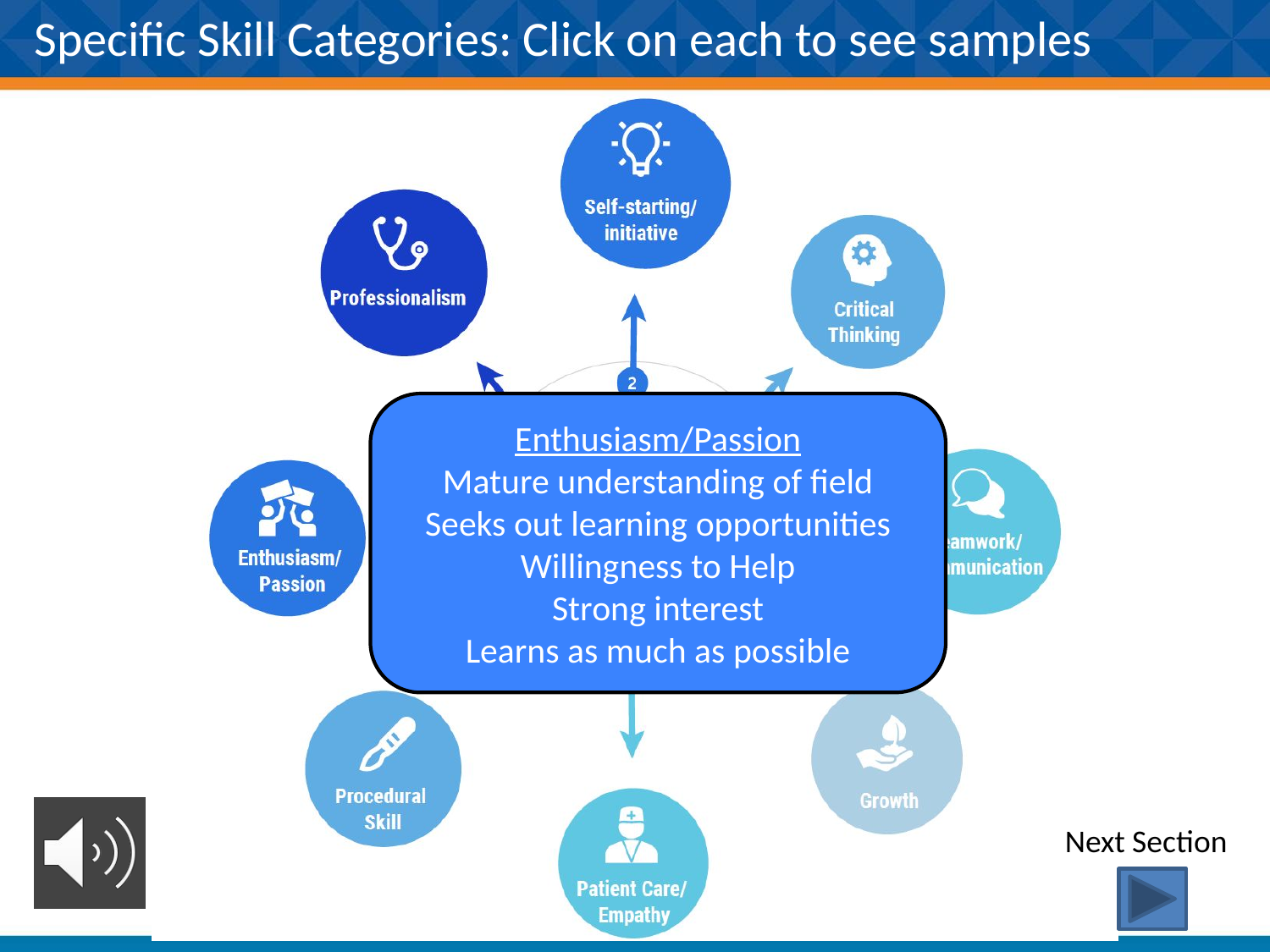

Specific Skill Categories: Click on each to see samples
Enthusiasm/Passion
Mature understanding of field
Seeks out learning opportunities
Willingness to Help
Strong interest
Learns as much as possible
Next Section

## Slide 35
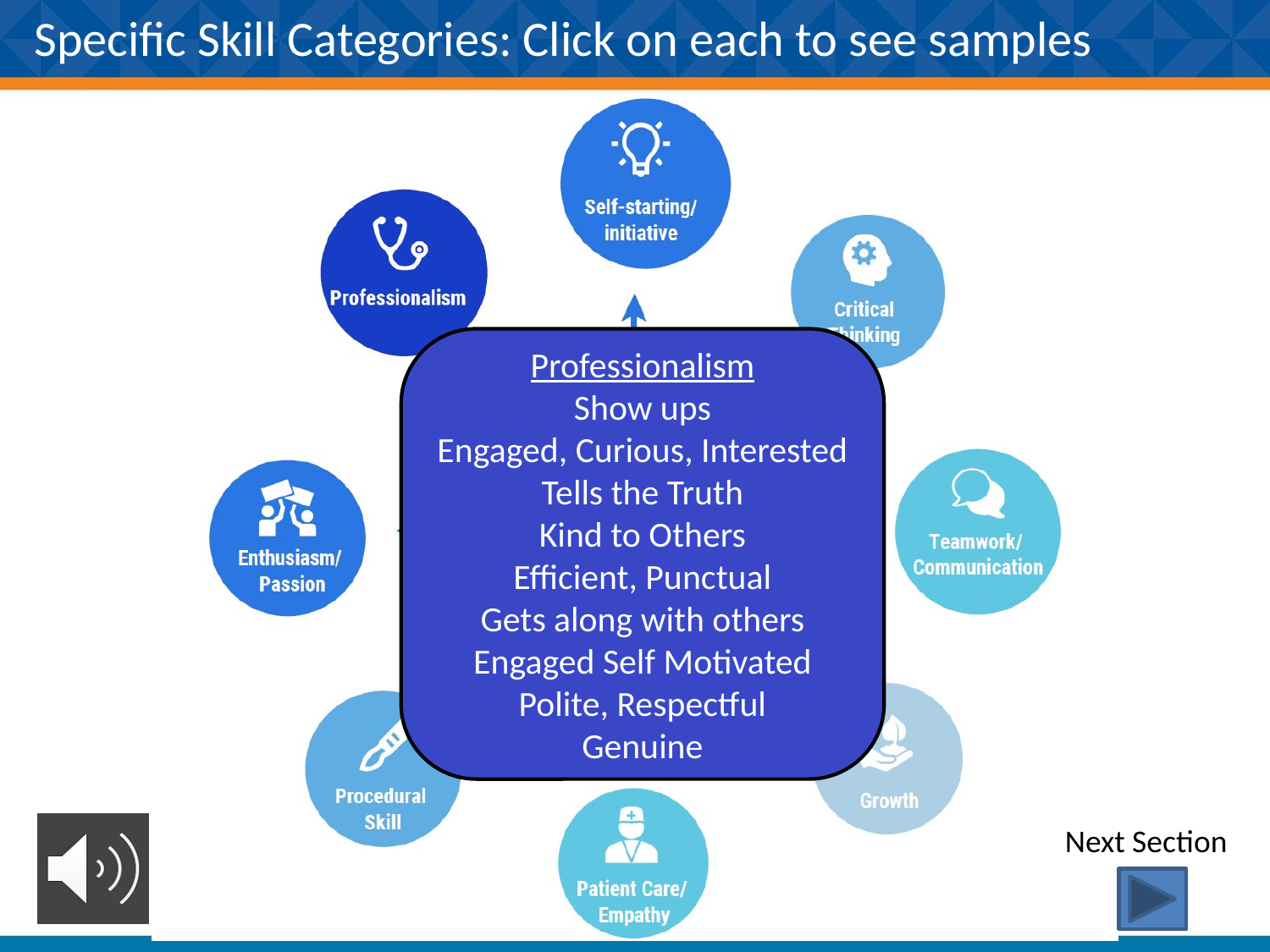

Specific Skill Categories: Click on each to see samples
Professionalism
Show ups
Engaged, Curious, Interested
Tells the Truth
Kind to Others
Efficient, Punctual
Gets along with others
Engaged Self Motivated
Polite, Respectful
Genuine
Next Section

## Slide 36
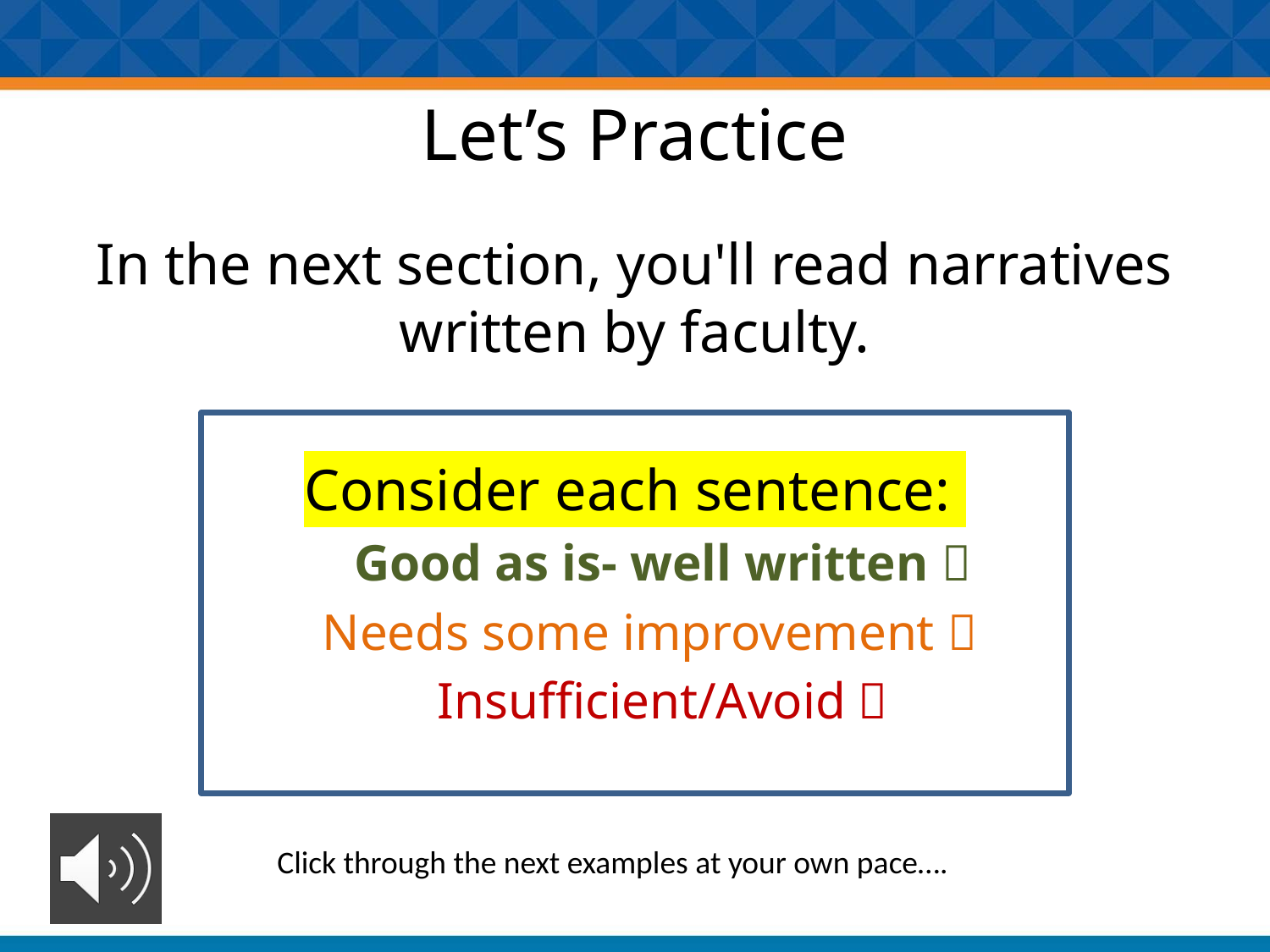

# Let’s Practice
In the next section, you'll read narratives written by faculty.
Consider each sentence:
Good as is- well written 
Needs some improvement 
Insufficient/Avoid 
Click through the next examples at your own pace….

## Slide 37
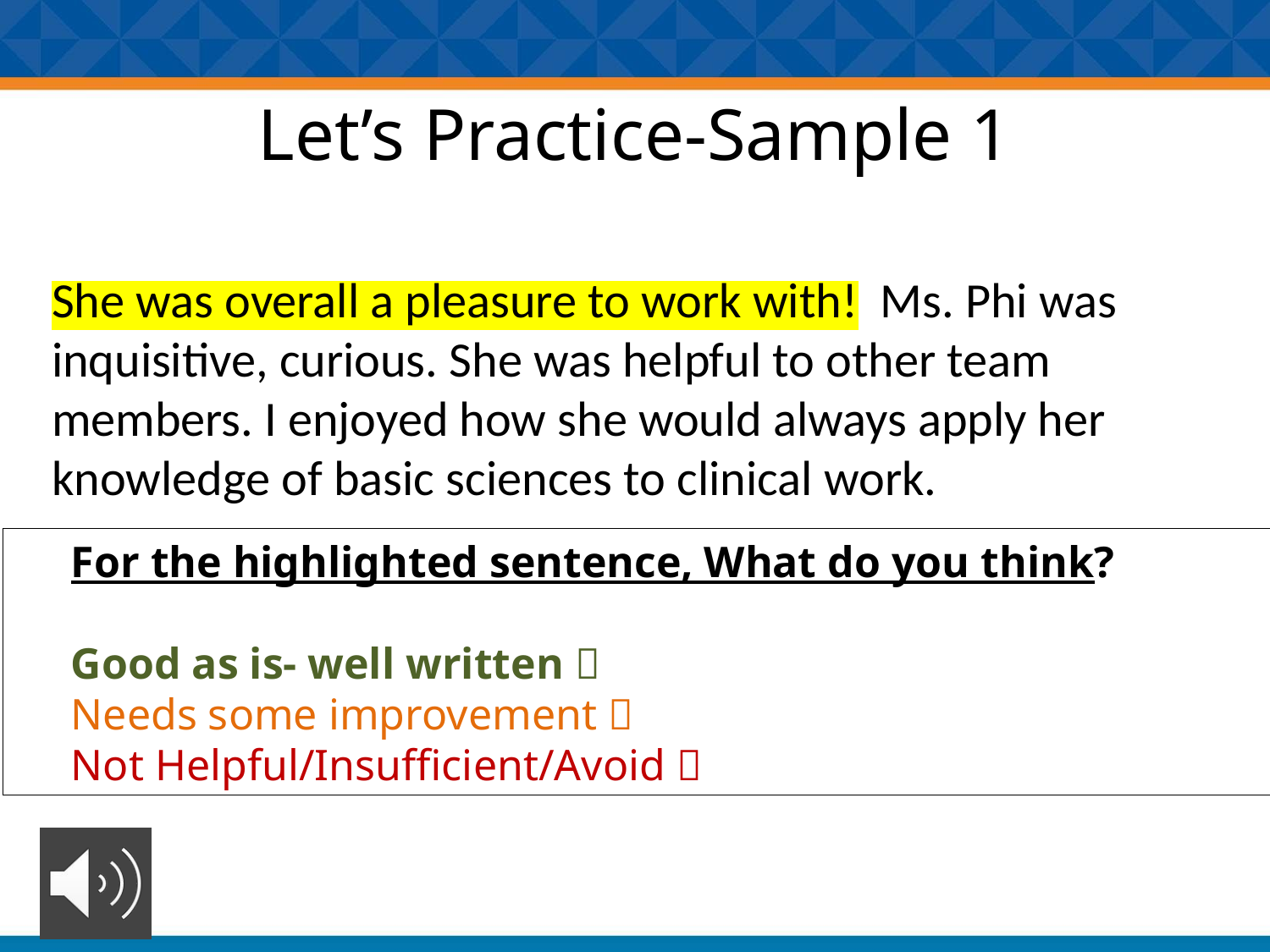

# Let’s Practice-Sample 1
​
She was overall a pleasure to work with! Ms. Phi was inquisitive, curious. She was helpful to other team members. I enjoyed how she would always apply her knowledge of basic sciences to clinical work.
For the highlighted sentence, What do you think?
Good as is- well written 
Needs some improvement 
Not Helpful/Insufficient/Avoid 

## Slide 38
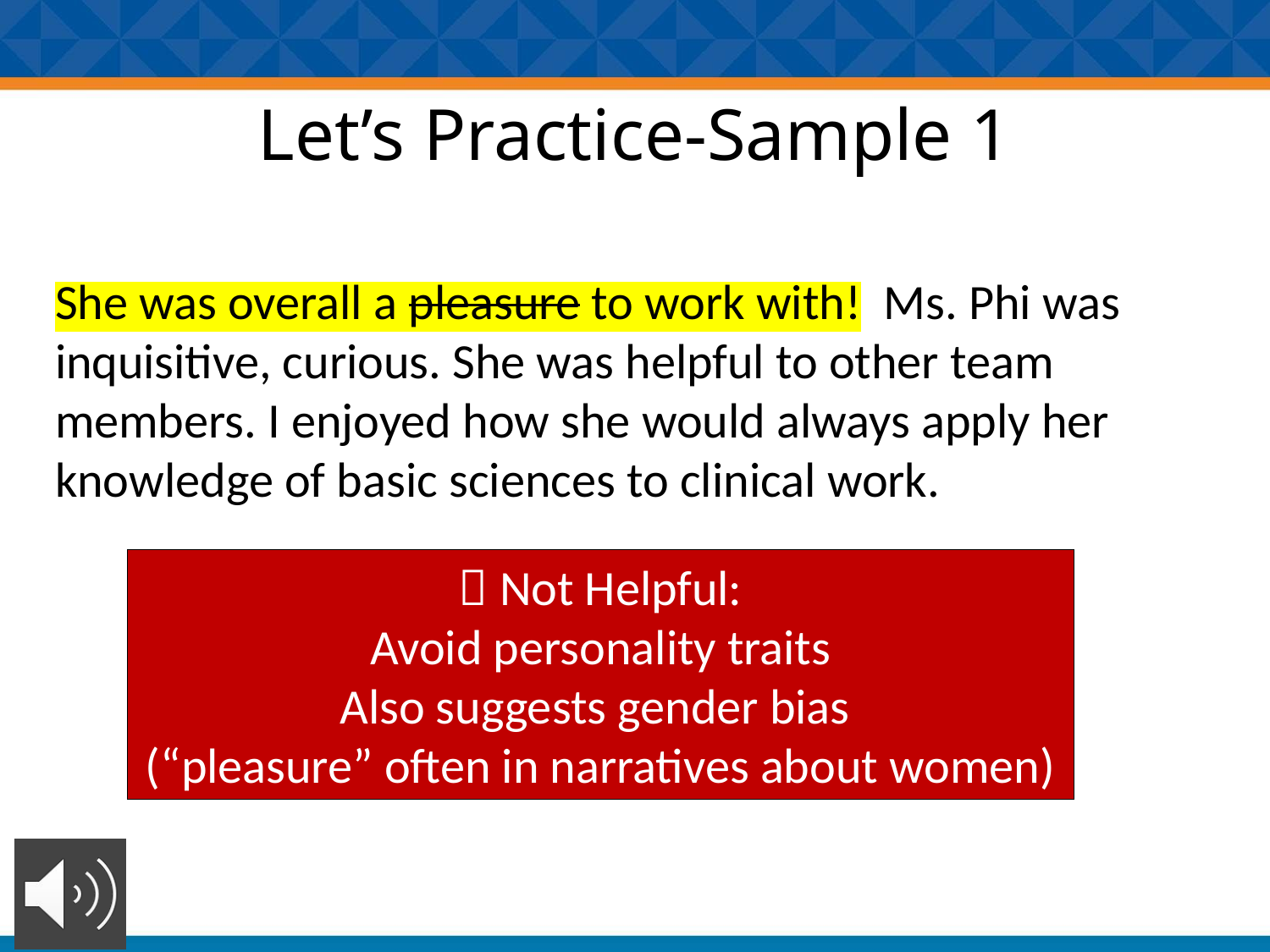

# Let’s Practice-Sample 1
​
She was overall a pleasure to work with! Ms. Phi was inquisitive, curious. She was helpful to other team members. I enjoyed how she would always apply her knowledge of basic sciences to clinical work.
 Not Helpful:
Avoid personality traits
Also suggests gender bias
(“pleasure” often in narratives about women)

## Slide 39
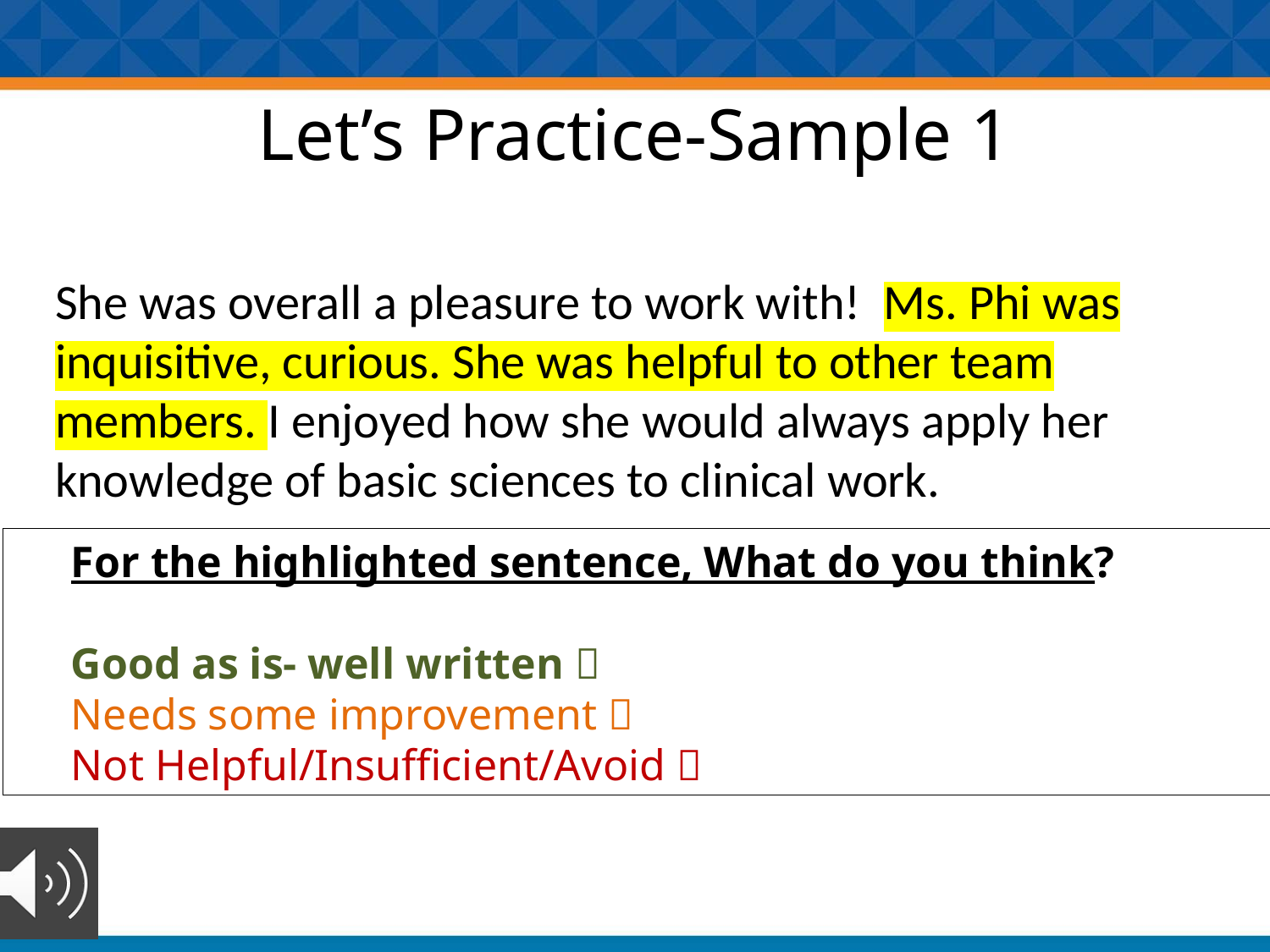

# Let’s Practice-Sample 1
​
She was overall a pleasure to work with! Ms. Phi was inquisitive, curious. She was helpful to other team members. I enjoyed how she would always apply her knowledge of basic sciences to clinical work.
For the highlighted sentence, What do you think?
Good as is- well written 
Needs some improvement 
Not Helpful/Insufficient/Avoid 

## Slide 40
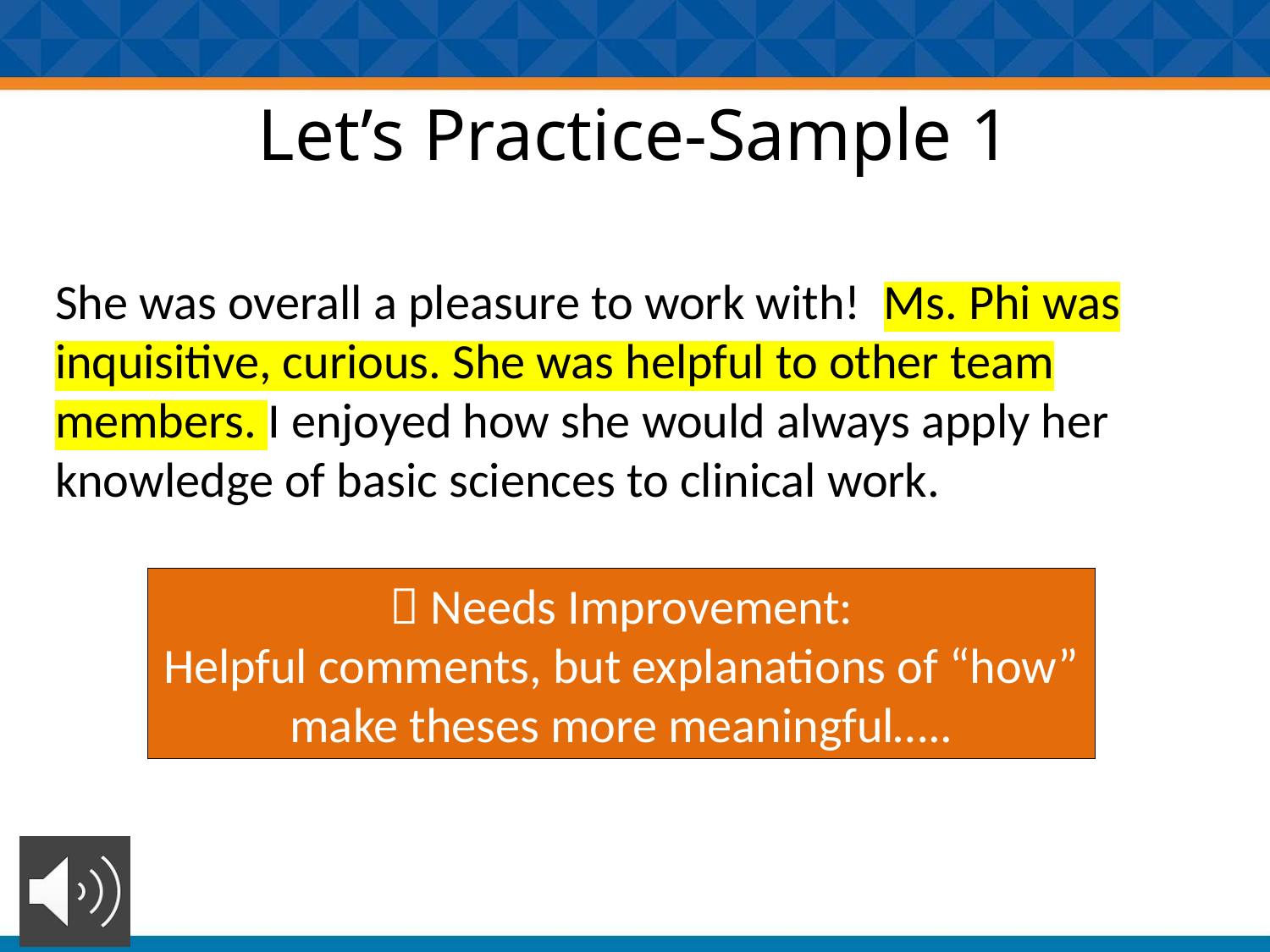

# Let’s Practice-Sample 1
​
She was overall a pleasure to work with! Ms. Phi was inquisitive, curious. She was helpful to other team members. I enjoyed how she would always apply her knowledge of basic sciences to clinical work.
 Needs Improvement:
Helpful comments, but explanations of “how” make theses more meaningful…..

## Slide 41
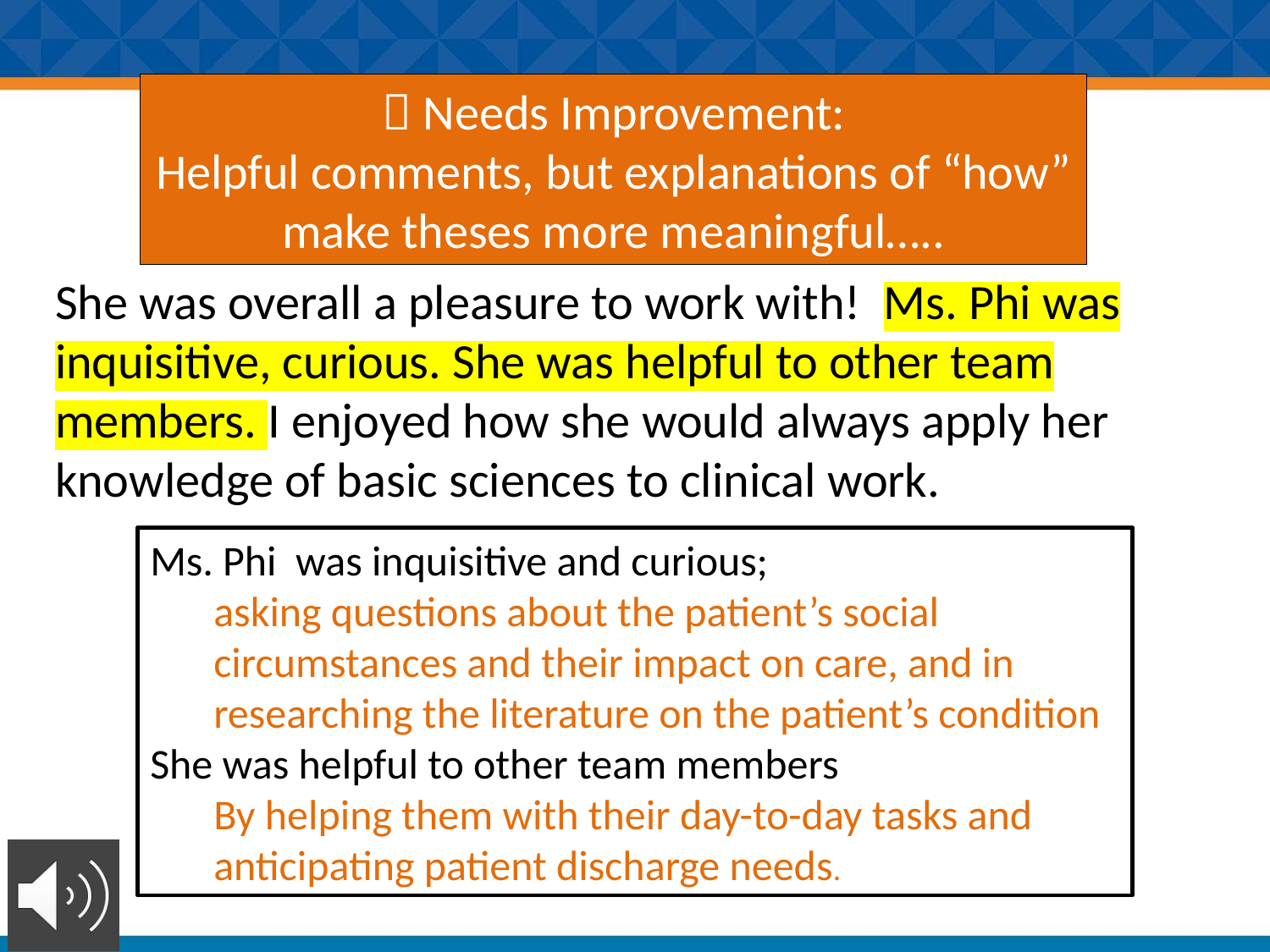

# Let’s Practice-Sample 1
 Needs Improvement:
Helpful comments, but explanations of “how” make theses more meaningful…..
​
She was overall a pleasure to work with! Ms. Phi was inquisitive, curious. She was helpful to other team members. I enjoyed how she would always apply her knowledge of basic sciences to clinical work.
Ms. Phi was inquisitive and curious;
asking questions about the patient’s social circumstances and their impact on care, and in researching the literature on the patient’s condition
She was helpful to other team members
By helping them with their day-to-day tasks and anticipating patient discharge needs.

## Slide 42
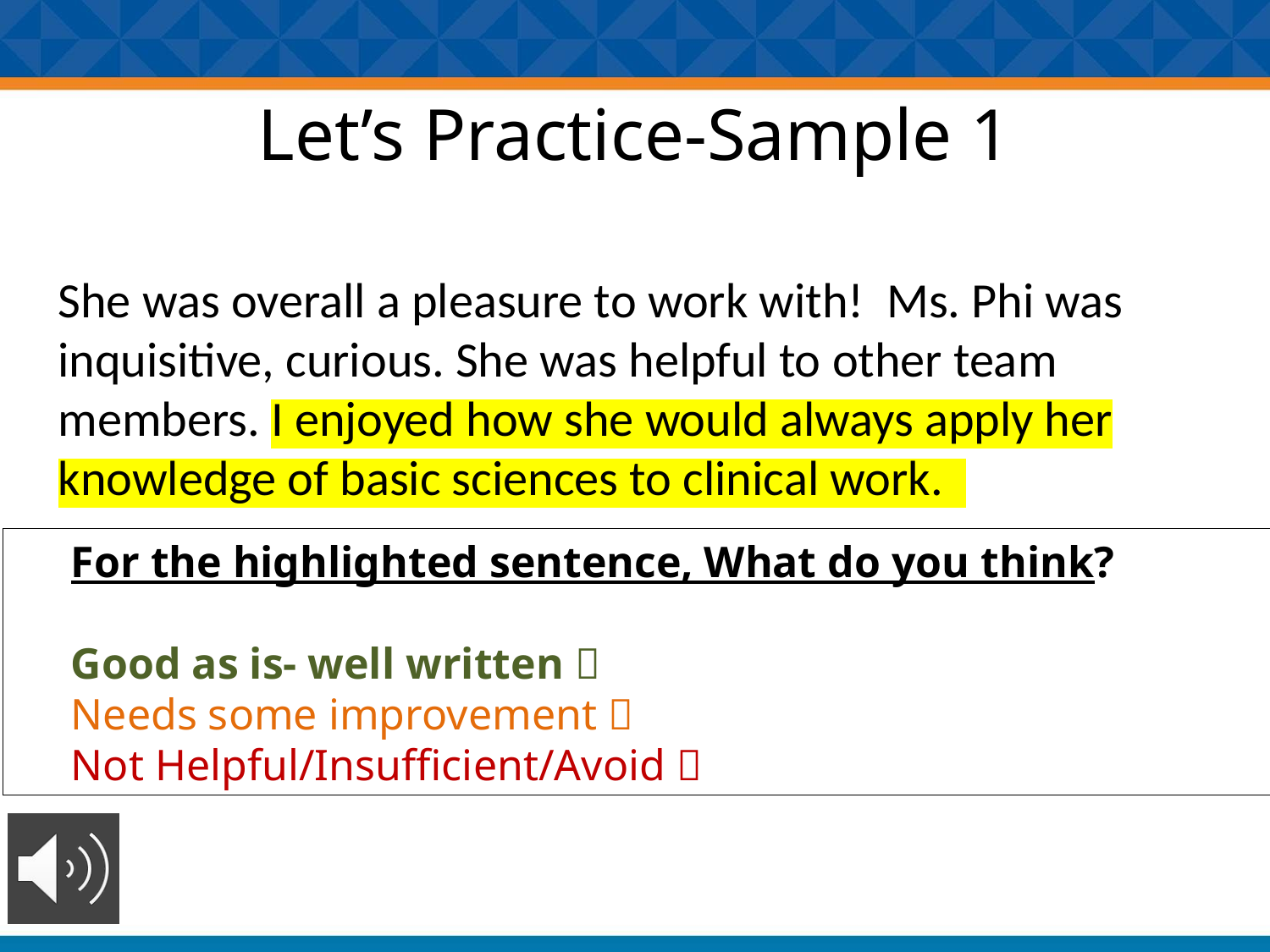

# Let’s Practice-Sample 1
​
She was overall a pleasure to work with! Ms. Phi was inquisitive, curious. She was helpful to other team members. I enjoyed how she would always apply her knowledge of basic sciences to clinical work.
For the highlighted sentence, What do you think?
Good as is- well written 
Needs some improvement 
Not Helpful/Insufficient/Avoid 

## Slide 43
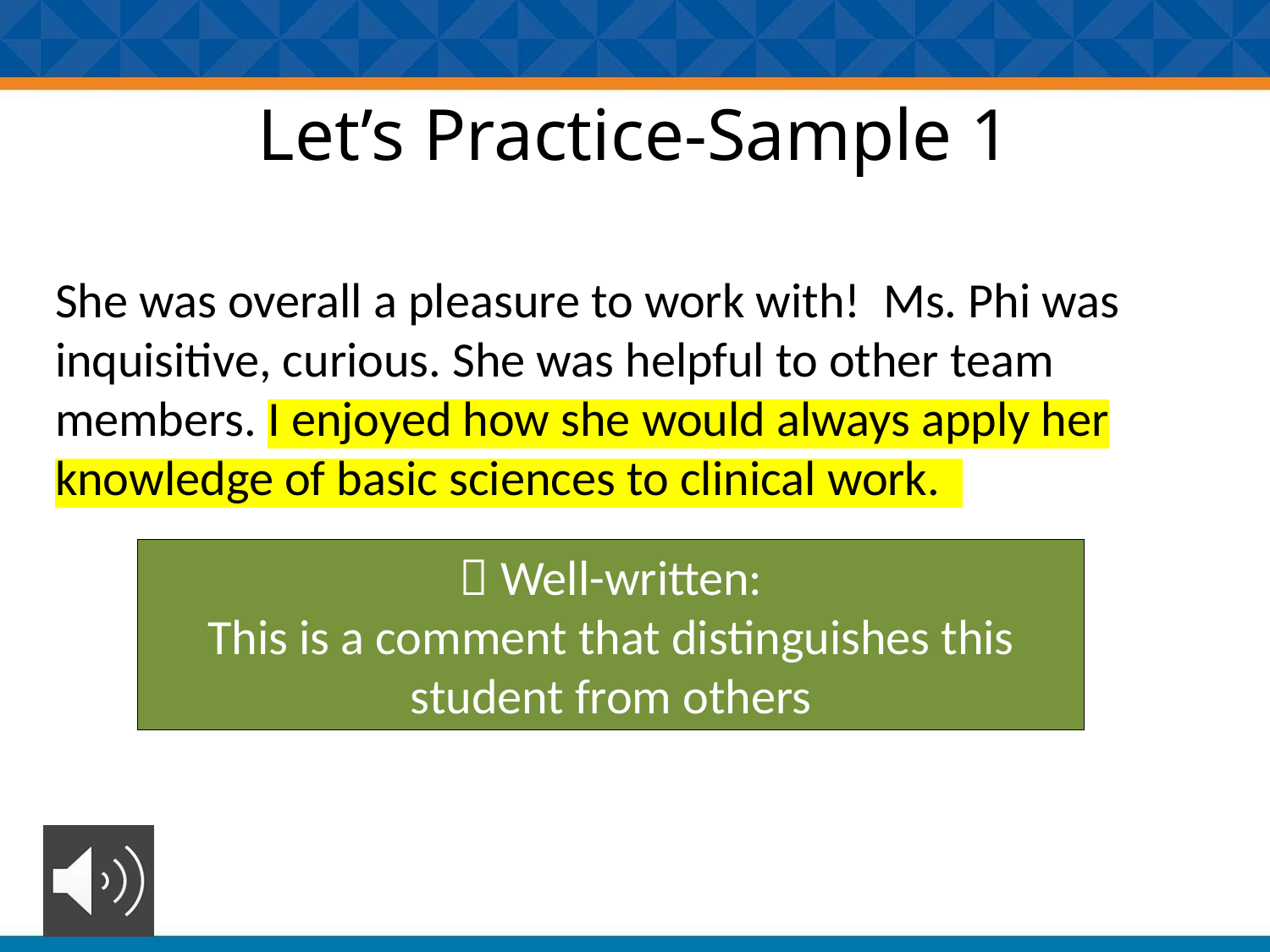

# Let’s Practice-Sample 1
​
She was overall a pleasure to work with! Ms. Phi was inquisitive, curious. She was helpful to other team members. I enjoyed how she would always apply her knowledge of basic sciences to clinical work.
 Well-written:
This is a comment that distinguishes this student from others

## Slide 44
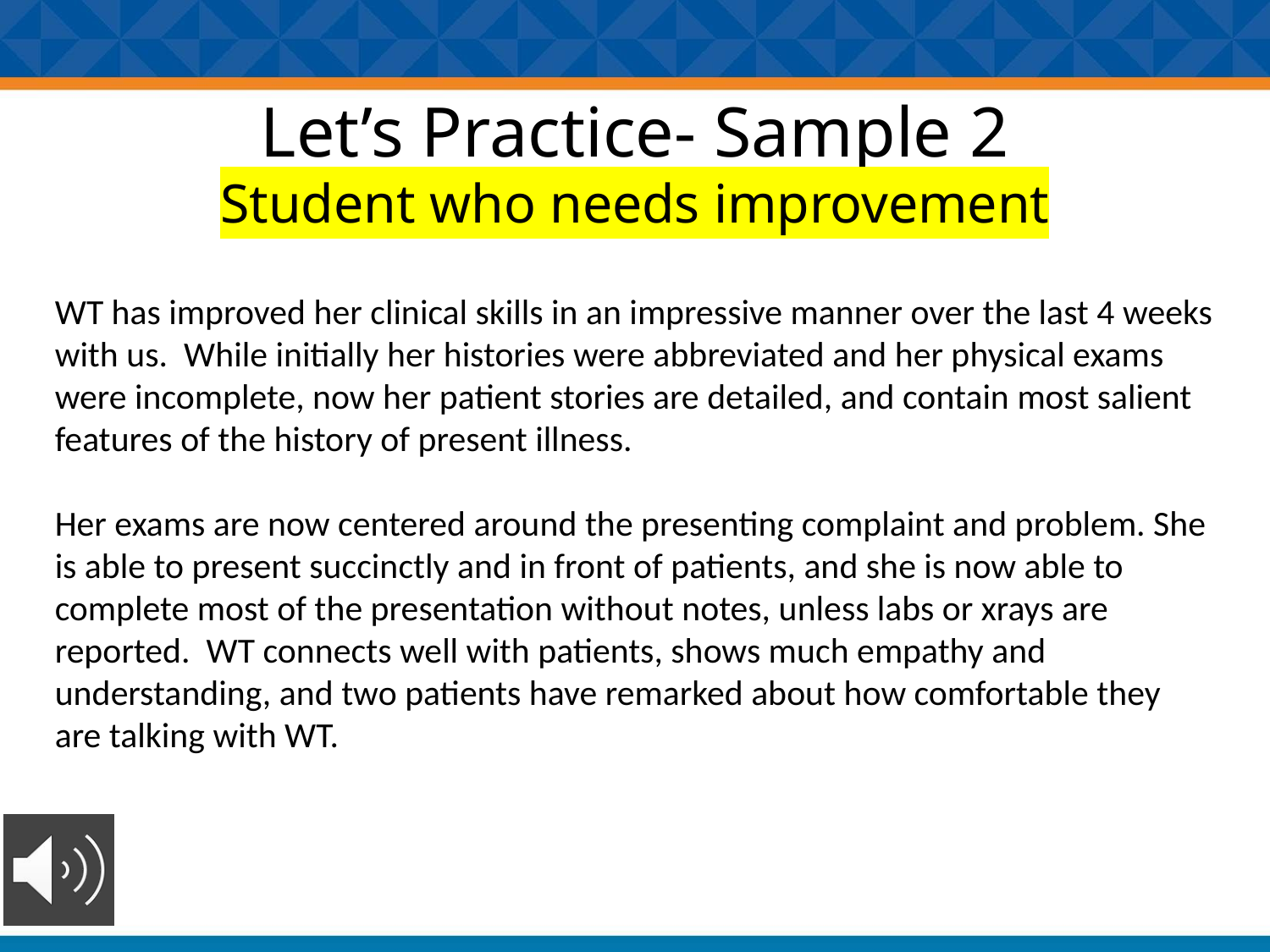

# Let’s Practice- Sample 2 Student who needs improvement
WT has improved her clinical skills in an impressive manner over the last 4 weeks with us.  While initially her histories were abbreviated and her physical exams were incomplete, now her patient stories are detailed, and contain most salient features of the history of present illness.
Her exams are now centered around the presenting complaint and problem. She is able to present succinctly and in front of patients, and she is now able to complete most of the presentation without notes, unless labs or xrays are reported.  WT connects well with patients, shows much empathy and understanding, and two patients have remarked about how comfortable they are talking with WT.

## Slide 45
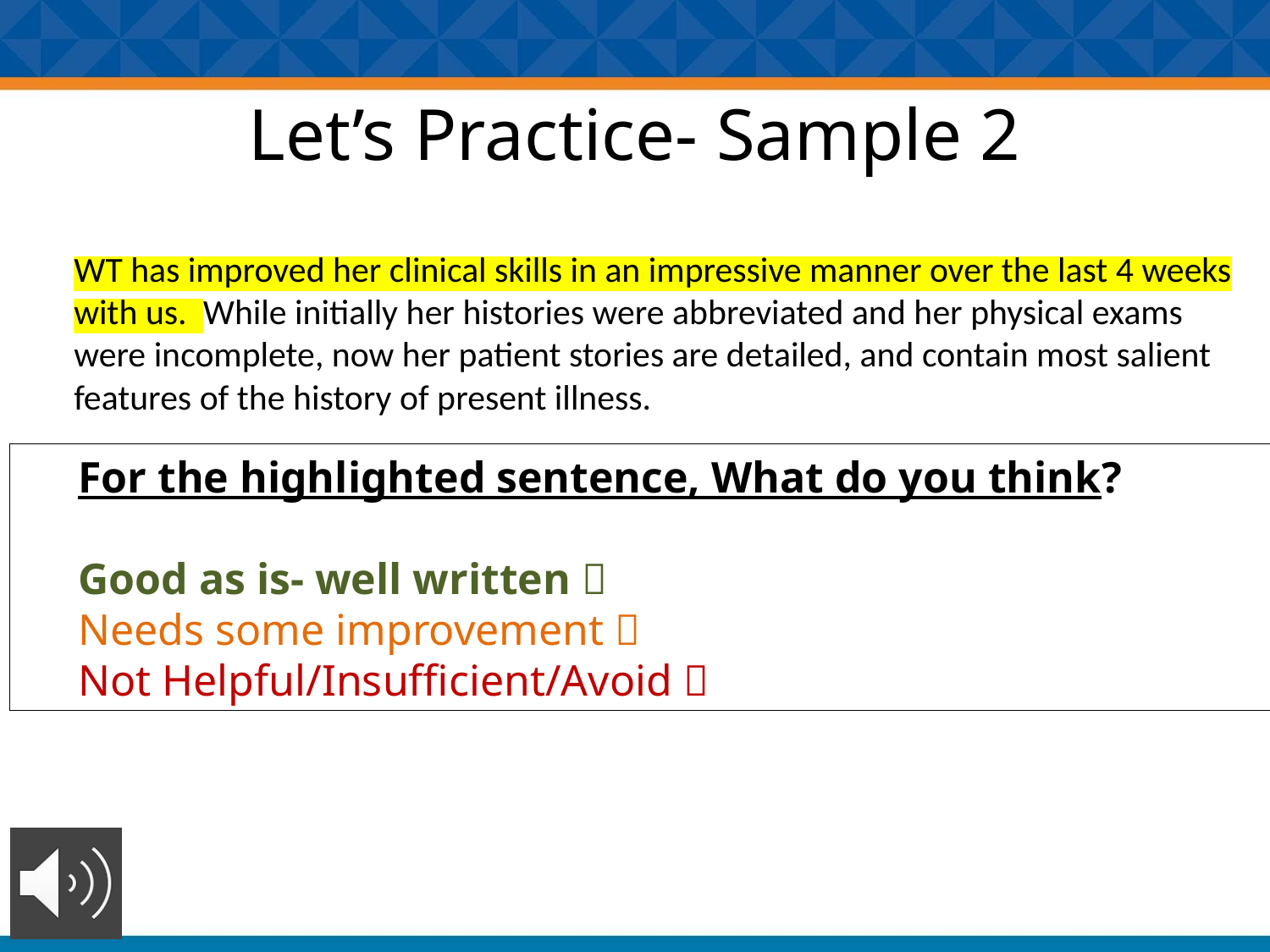

# Let’s Practice- Sample 2
WT has improved her clinical skills in an impressive manner over the last 4 weeks with us.  While initially her histories were abbreviated and her physical exams were incomplete, now her patient stories are detailed, and contain most salient features of the history of present illness.
Her exams are now centered around the presenting complaint and problem. She is able to present succinctly and in front of patients, and she is now able to complete most of the presentation without notes, unless labs or xrays are reported.  WT connects well with patients, shows much empathy and understanding, and two patients have remarked about how comfortable they are talking with WT.
For the highlighted sentence, What do you think?
Good as is- well written 
Needs some improvement 
Not Helpful/Insufficient/Avoid 

## Slide 46
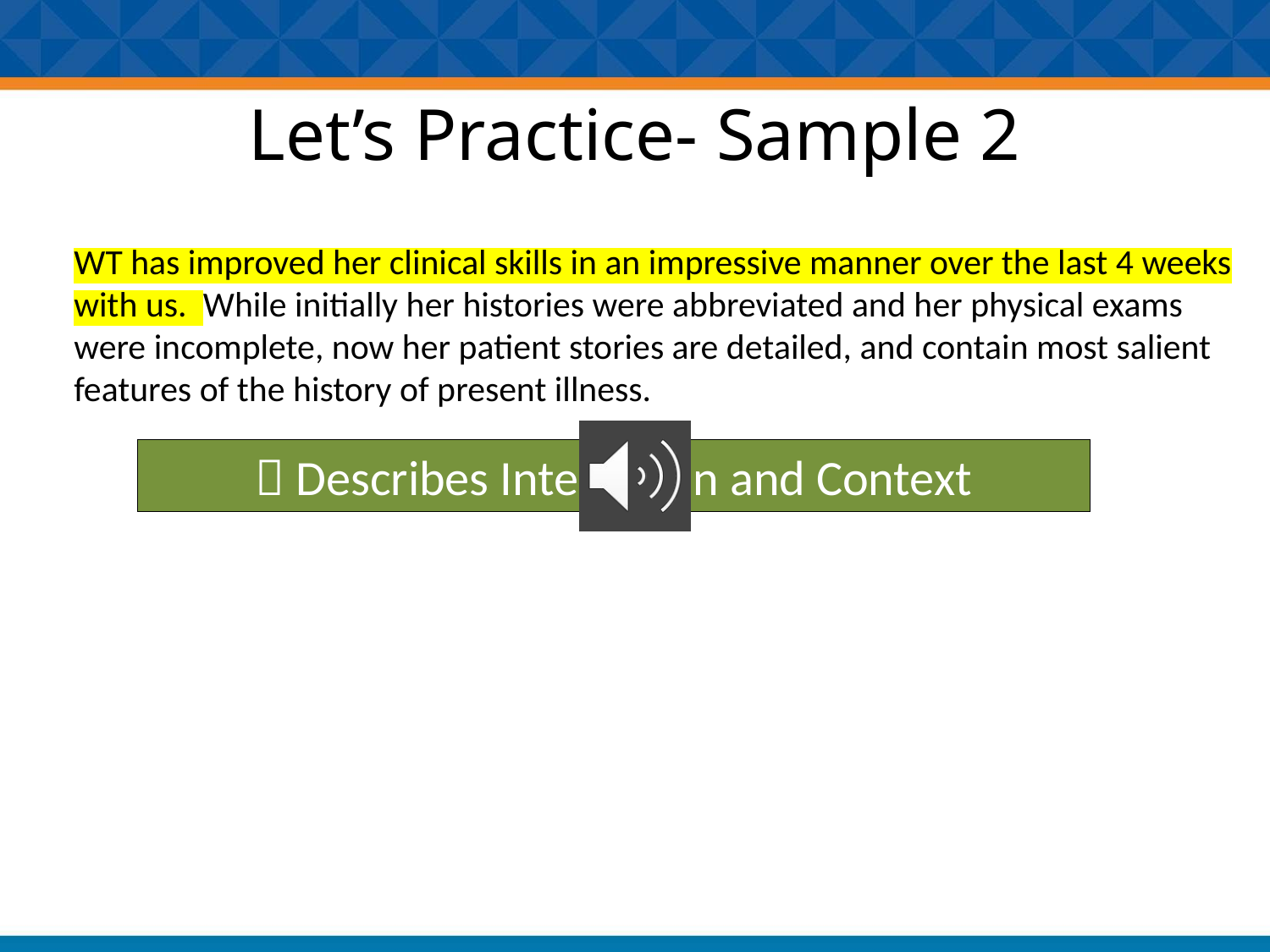

# Let’s Practice- Sample 2
WT has improved her clinical skills in an impressive manner over the last 4 weeks with us.  While initially her histories were abbreviated and her physical exams were incomplete, now her patient stories are detailed, and contain most salient features of the history of present illness.
 Describes Interaction and Context

## Slide 47
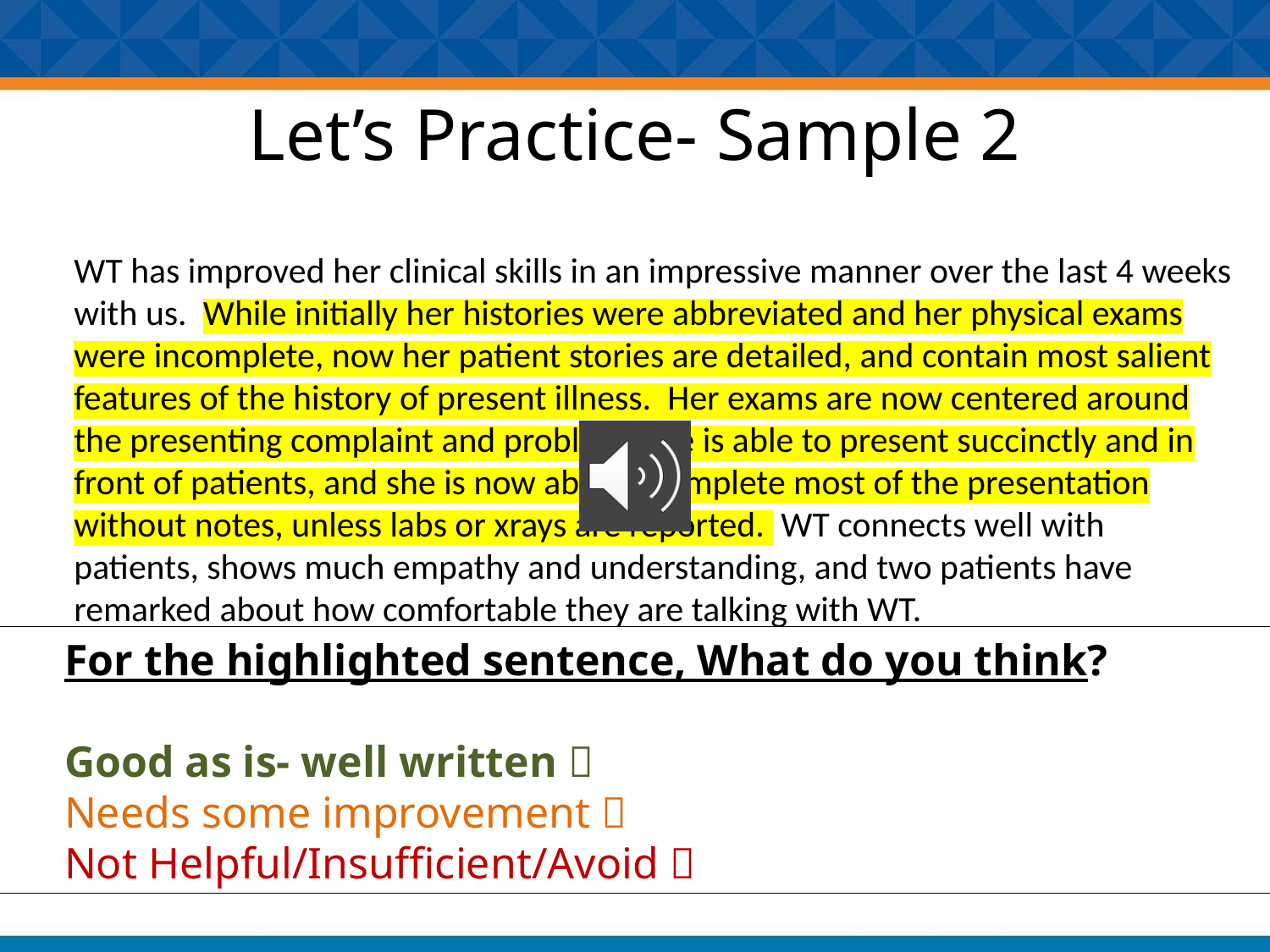

# Let’s Practice- Sample 2
WT has improved her clinical skills in an impressive manner over the last 4 weeks with us.  While initially her histories were abbreviated and her physical exams were incomplete, now her patient stories are detailed, and contain most salient features of the history of present illness.  Her exams are now centered around the presenting complaint and problem. She is able to present succinctly and in front of patients, and she is now able to complete most of the presentation without notes, unless labs or xrays are reported.  WT connects well with patients, shows much empathy and understanding, and two patients have remarked about how comfortable they are talking with WT.
.
For the highlighted sentence, What do you think?
Good as is- well written 
Needs some improvement 
Not Helpful/Insufficient/Avoid 

## Slide 48
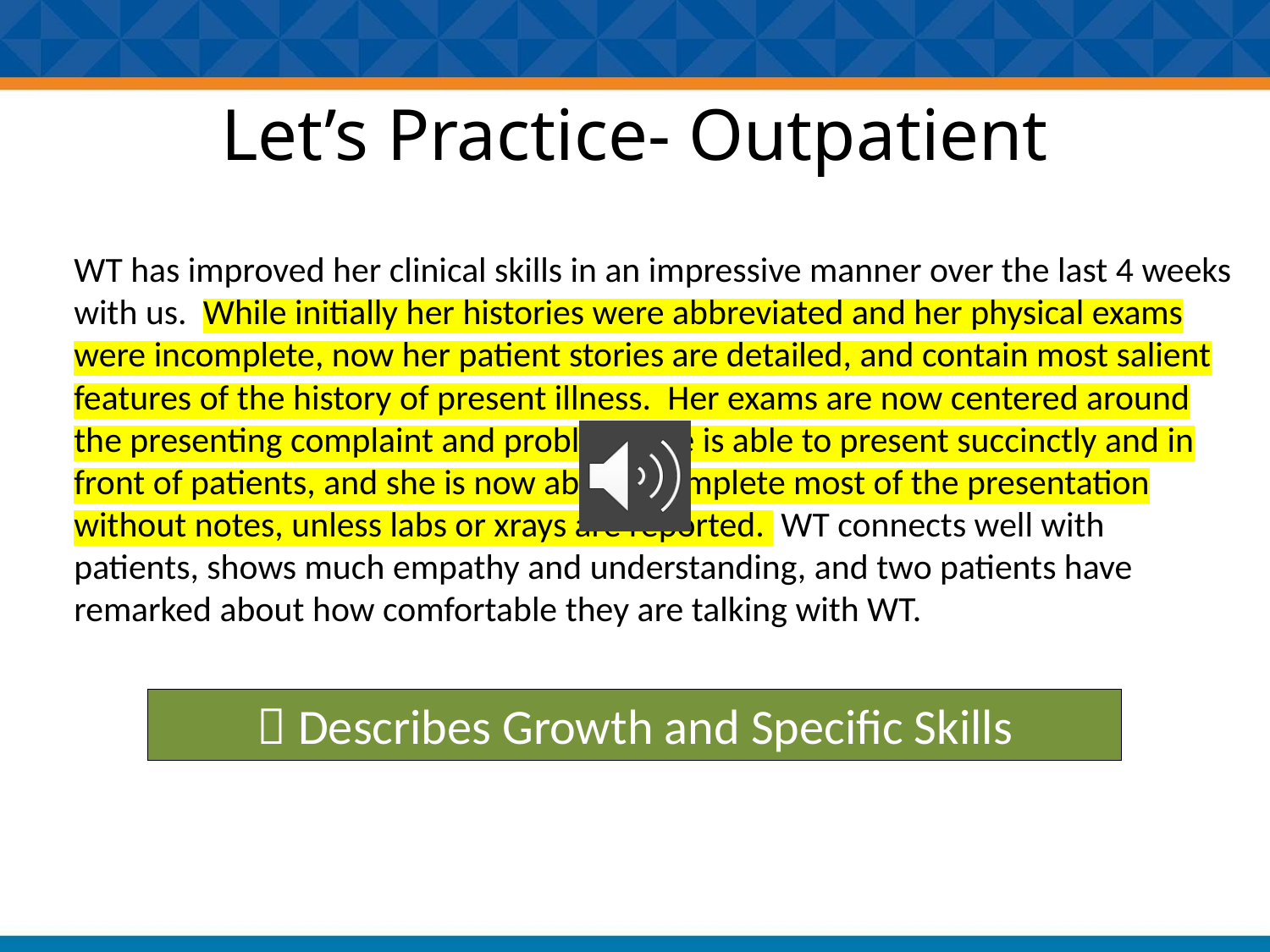

# Let’s Practice- Outpatient
WT has improved her clinical skills in an impressive manner over the last 4 weeks with us.  While initially her histories were abbreviated and her physical exams were incomplete, now her patient stories are detailed, and contain most salient features of the history of present illness.  Her exams are now centered around the presenting complaint and problem. She is able to present succinctly and in front of patients, and she is now able to complete most of the presentation without notes, unless labs or xrays are reported.  WT connects well with patients, shows much empathy and understanding, and two patients have remarked about how comfortable they are talking with WT.
 Describes Growth and Specific Skills

## Slide 49
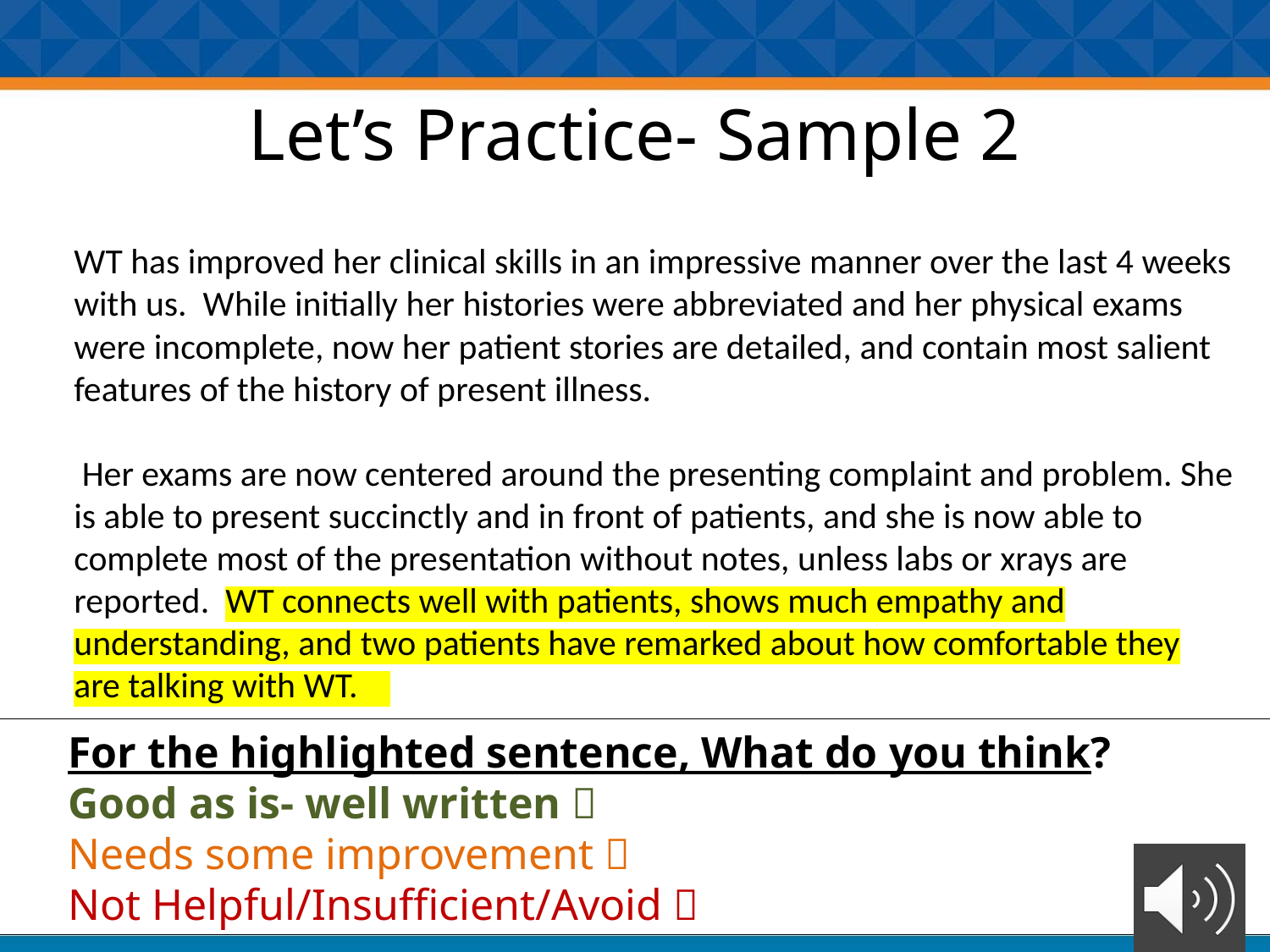

# Let’s Practice- Sample 2
WT has improved her clinical skills in an impressive manner over the last 4 weeks with us.  While initially her histories were abbreviated and her physical exams were incomplete, now her patient stories are detailed, and contain most salient features of the history of present illness.
 Her exams are now centered around the presenting complaint and problem. She is able to present succinctly and in front of patients, and she is now able to complete most of the presentation without notes, unless labs or xrays are reported.  WT connects well with patients, shows much empathy and understanding, and two patients have remarked about how comfortable they are talking with WT.
For the highlighted sentence, What do you think?
Good as is- well written 
Needs some improvement 
Not Helpful/Insufficient/Avoid 

## Slide 50
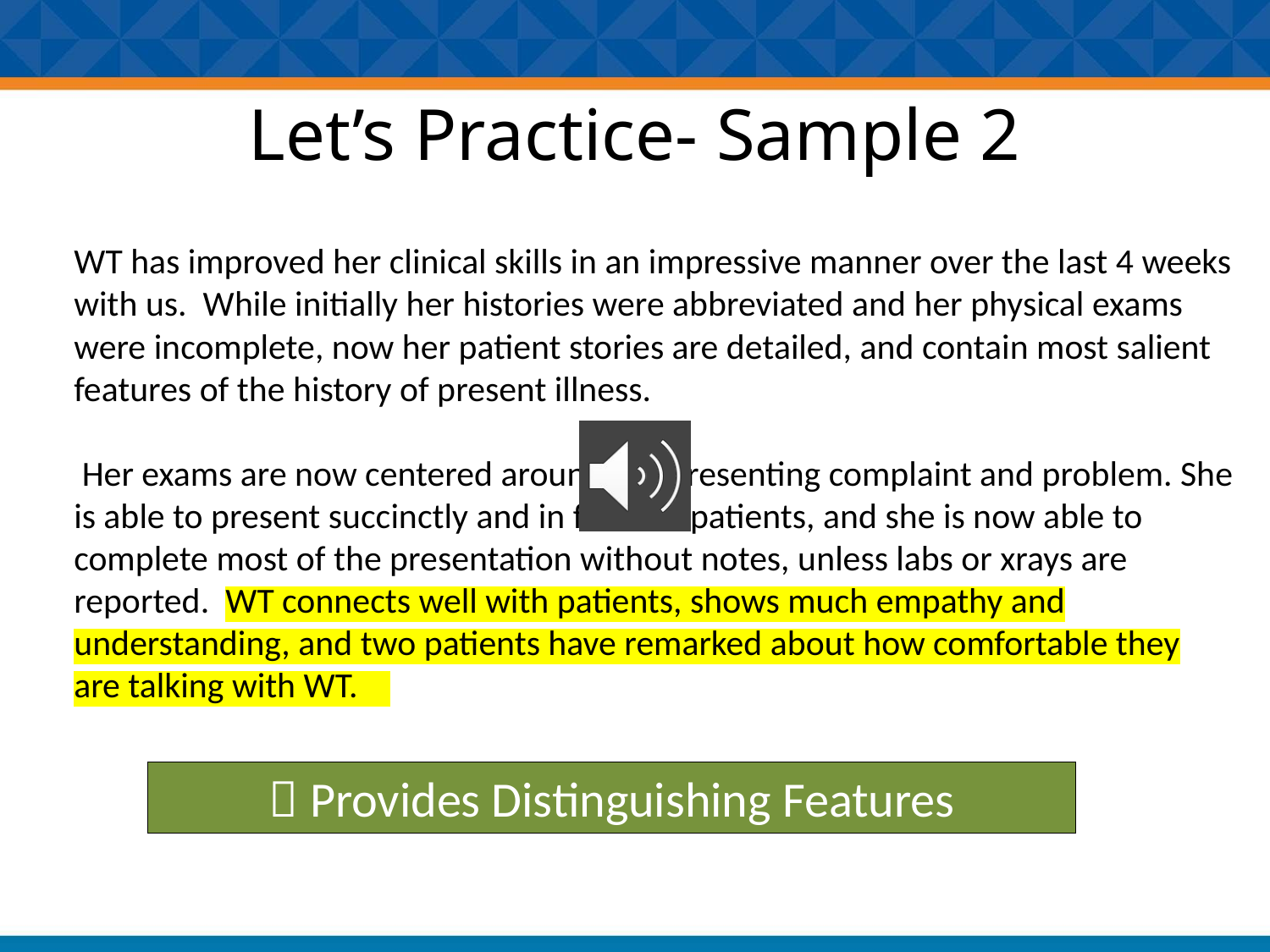

# Let’s Practice- Sample 2
WT has improved her clinical skills in an impressive manner over the last 4 weeks with us.  While initially her histories were abbreviated and her physical exams were incomplete, now her patient stories are detailed, and contain most salient features of the history of present illness.
 Her exams are now centered around the presenting complaint and problem. She is able to present succinctly and in front of patients, and she is now able to complete most of the presentation without notes, unless labs or xrays are reported.  WT connects well with patients, shows much empathy and understanding, and two patients have remarked about how comfortable they are talking with WT.
 Provides Distinguishing Features

## Slide 51
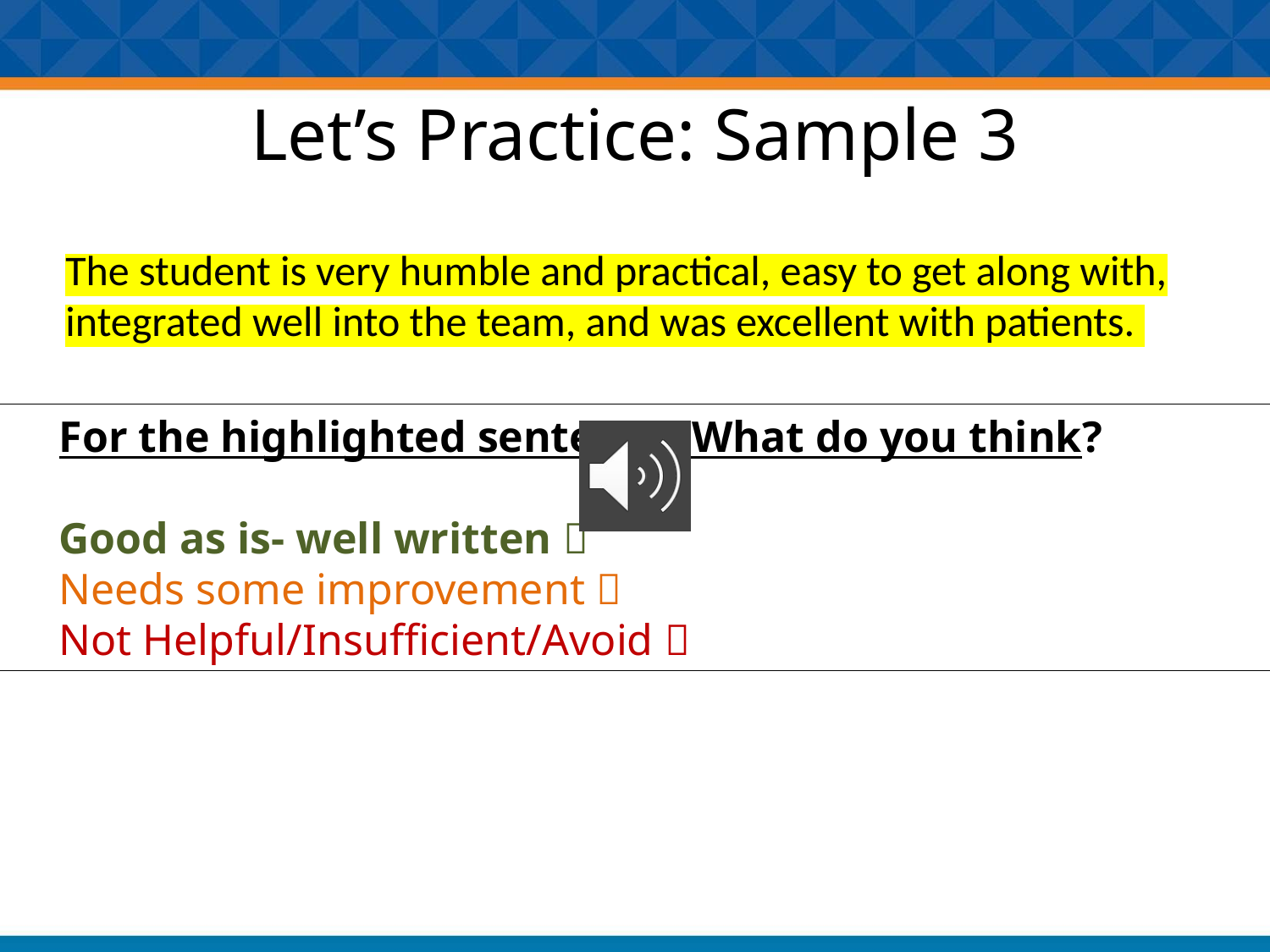

# Let’s Practice: Sample 3
​
The student is very humble and practical, easy to get along with, integrated well into the team, and was excellent with patients.
For the highlighted sentence, What do you think?
Good as is- well written 
Needs some improvement 
Not Helpful/Insufficient/Avoid 

## Slide 52
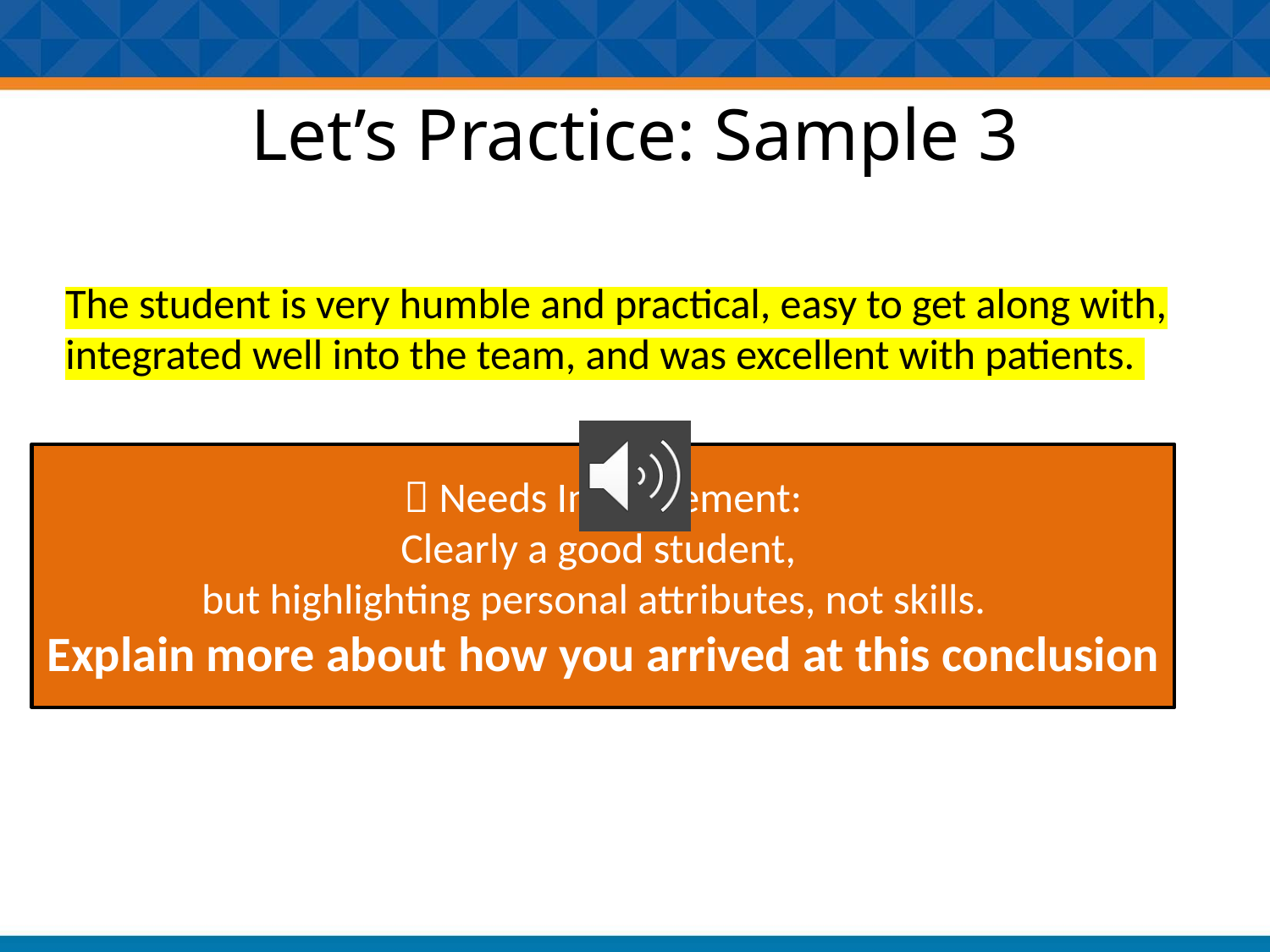

# Let’s Practice: Sample 3
​
The student is very humble and practical, easy to get along with, integrated well into the team, and was excellent with patients.
 Needs Improvement:
Clearly a good student,
but highlighting personal attributes, not skills.  
Explain more about how you arrived at this conclusion

## Slide 53
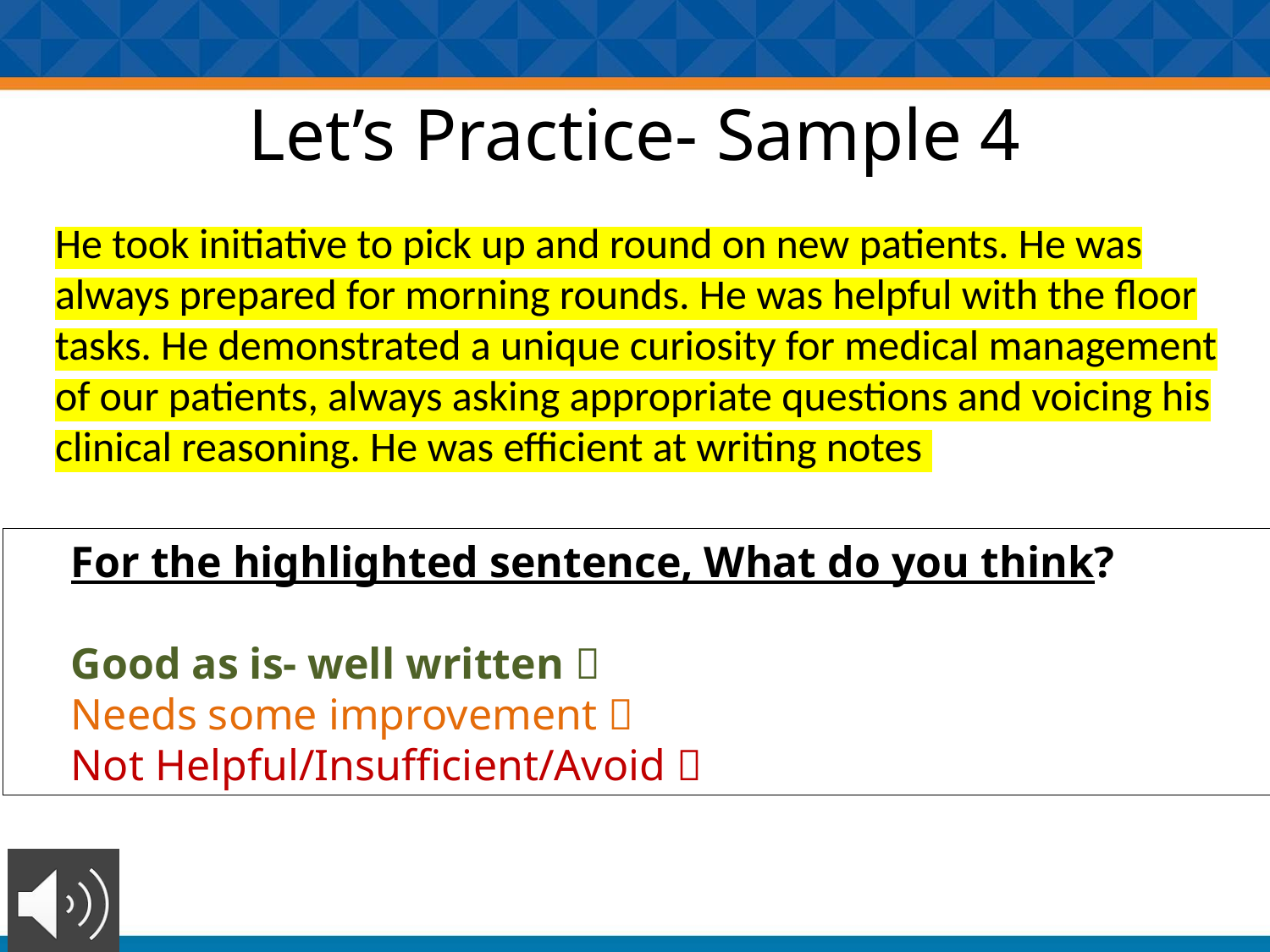

# Let’s Practice- Sample 4
​
He took initiative to pick up and round on new patients. He was always prepared for morning rounds. He was helpful with the floor tasks. He demonstrated a unique curiosity for medical management of our patients, always asking appropriate questions and voicing his clinical reasoning. He was efficient at writing notes
For the highlighted sentence, What do you think?
Good as is- well written 
Needs some improvement 
Not Helpful/Insufficient/Avoid 

## Slide 54
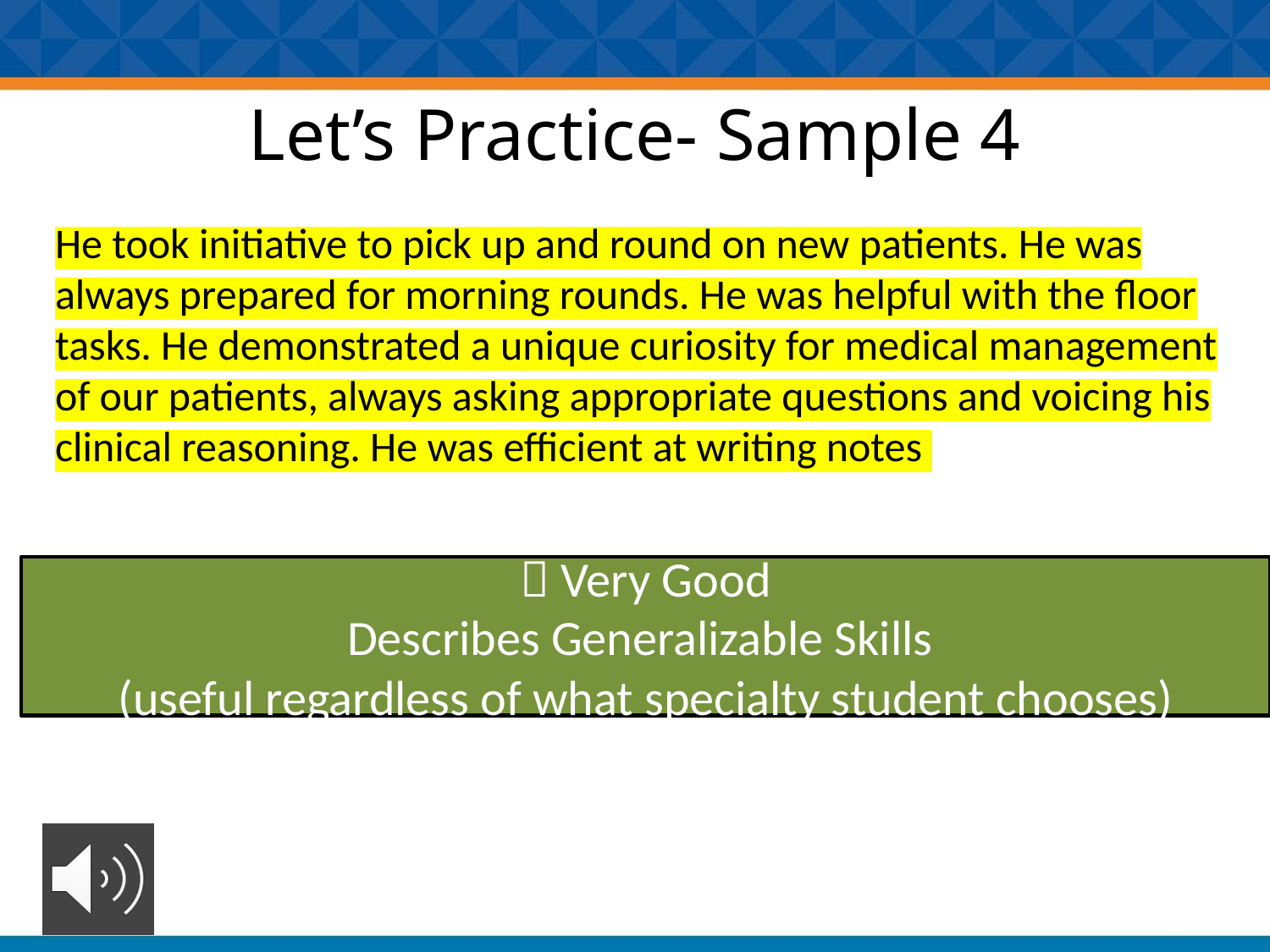

# Let’s Practice- Sample 4
​
He took initiative to pick up and round on new patients. He was always prepared for morning rounds. He was helpful with the floor tasks. He demonstrated a unique curiosity for medical management of our patients, always asking appropriate questions and voicing his clinical reasoning. He was efficient at writing notes
 Very Good
Describes Generalizable Skills
(useful regardless of what specialty student chooses)

## Slide 55
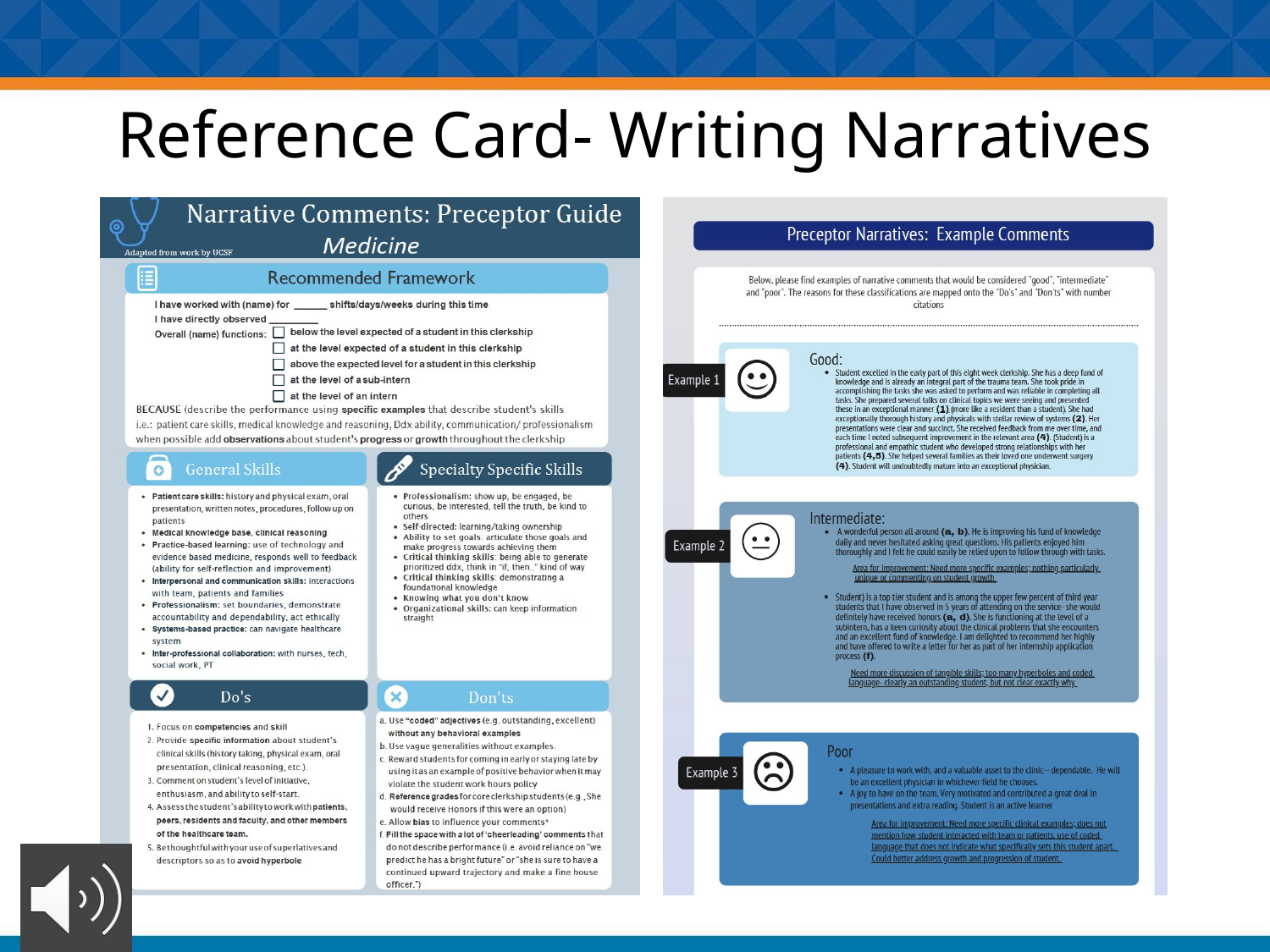

# Reference Card- Writing Narratives

## Slide 56
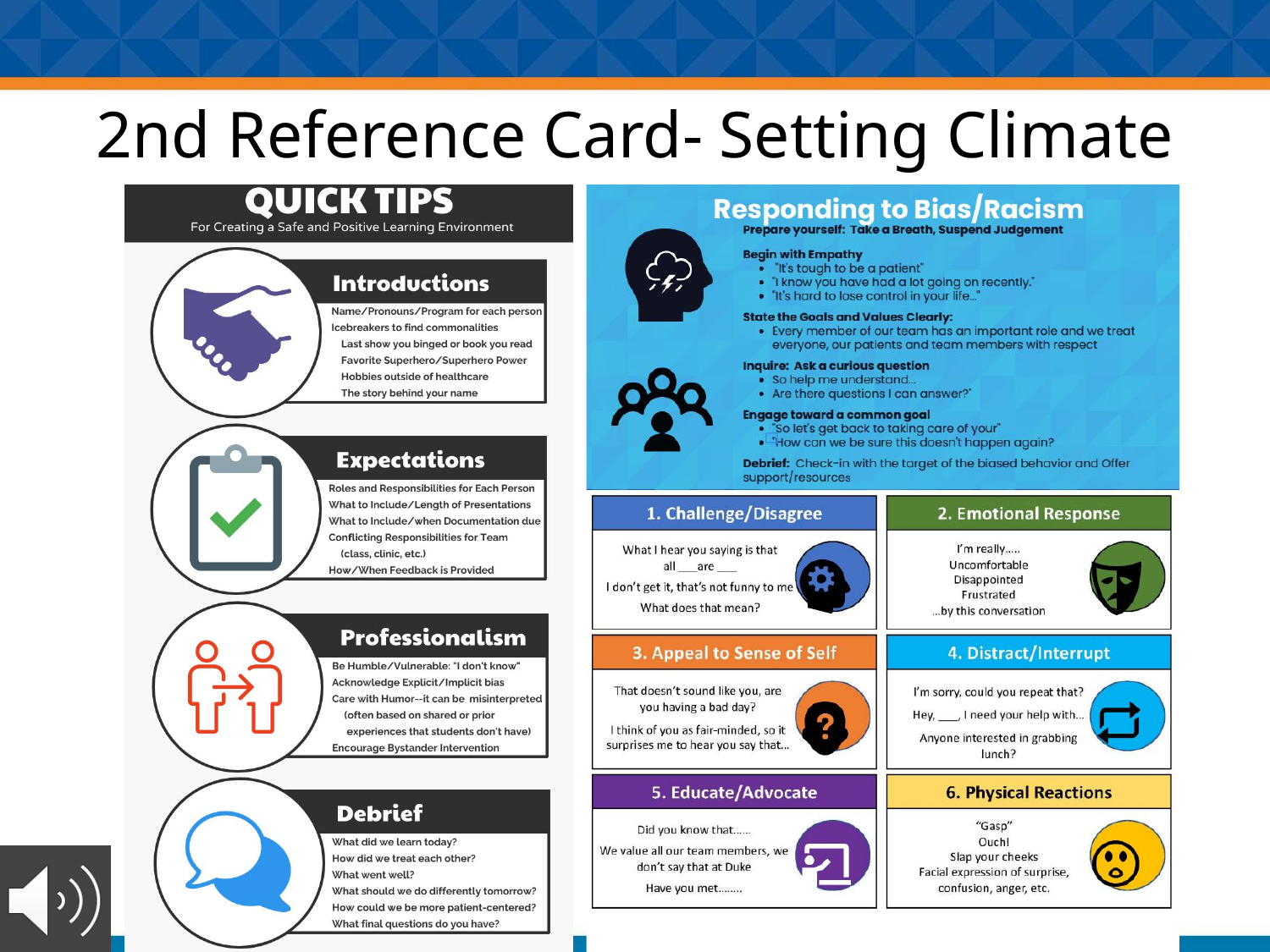

# 2nd Reference Card- Setting Climate

## Slide 57
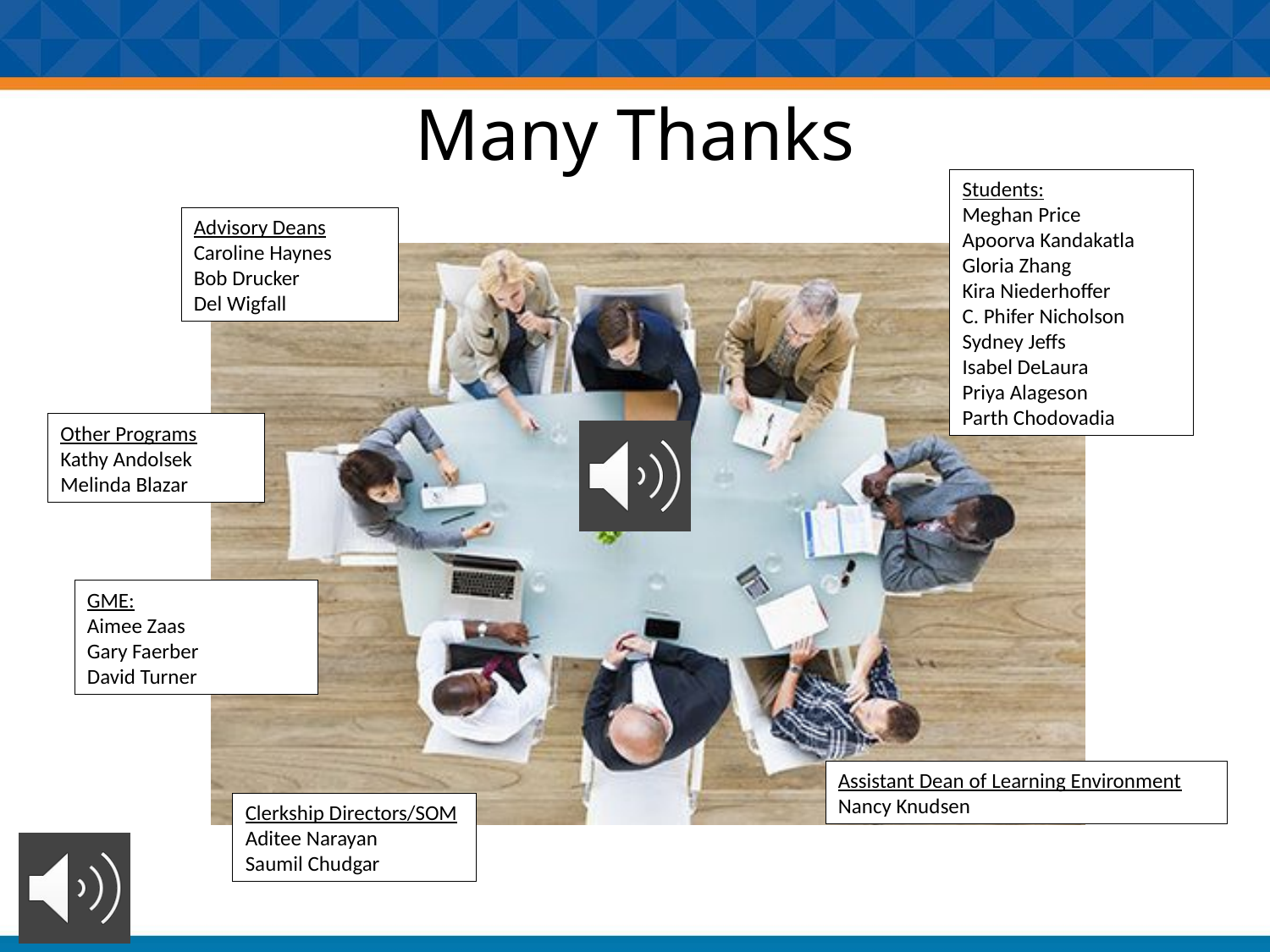

# Many Thanks
Students:
Meghan Price
Apoorva Kandakatla
Gloria Zhang
Kira Niederhoffer
C. Phifer Nicholson
Sydney Jeffs
Isabel DeLaura
Priya Alageson
Parth Chodovadia
Advisory Deans
Caroline Haynes
Bob Drucker
Del Wigfall
Other Programs
Kathy Andolsek
Melinda Blazar
GME:
Aimee Zaas
Gary Faerber
David Turner
Assistant Dean of Learning Environment
Nancy Knudsen
Clerkship Directors/SOM
Aditee Narayan
Saumil Chudgar

## Slide 58
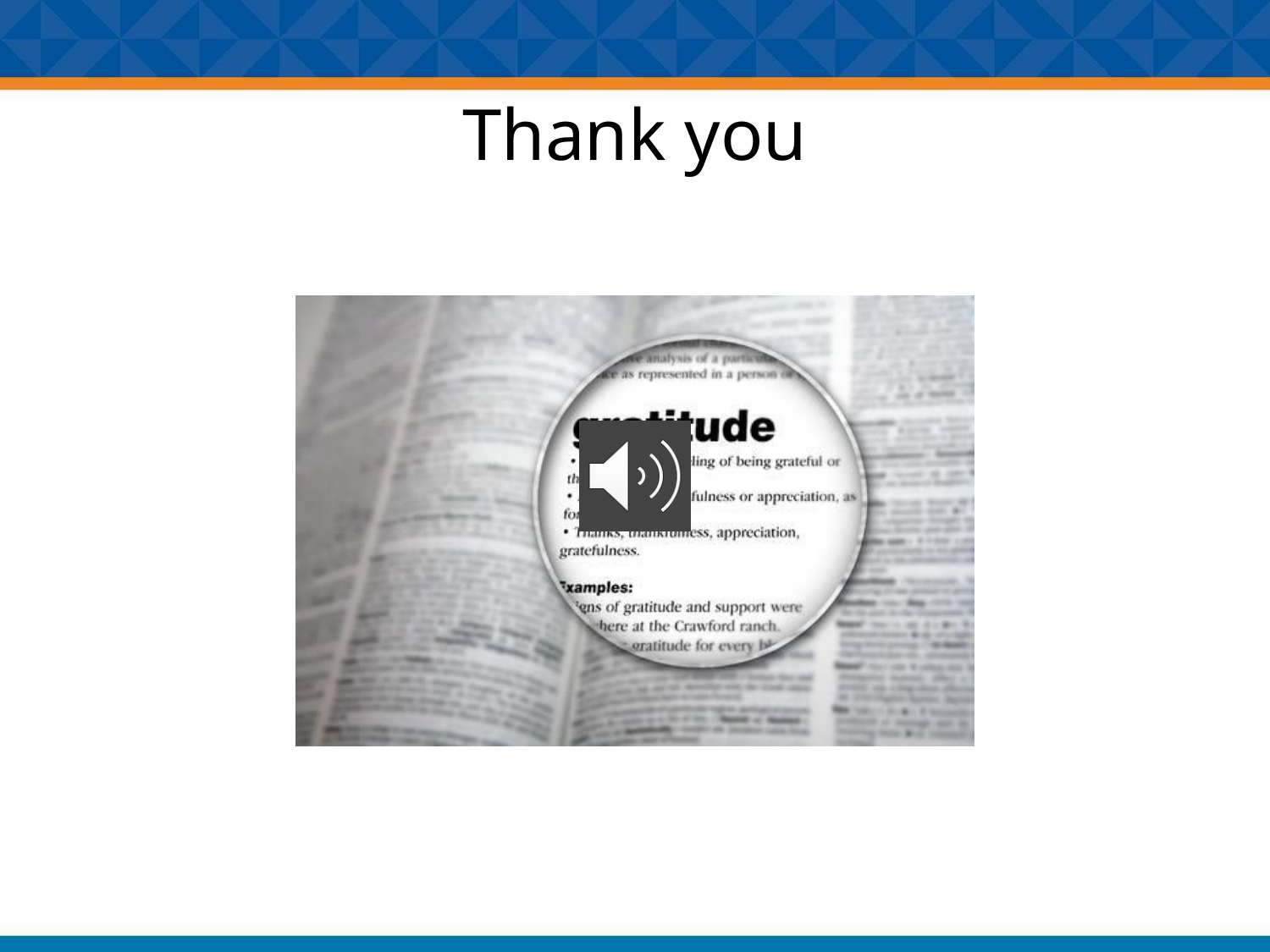

# Thank you
